# Synthesis of a Station‐Less Molecular Daisy Chain

**DOI:** 10.1002/chem.202501369

**Published:** 2025-05-12

**Authors:** Charlotte Kress, Daniel Häussinger, David A. Leigh, Marcel Mayor

**Affiliations:** ^1^ Department of Chemistry University of Basel St. Johanns‐Ring 19 Basel 4056 Switzerland; ^2^ Department of Chemistry The University of Manchester Oxford Road Manchester M13 9PL UK; ^3^ School of Chemistry and Molecular Engineering East China Normal University Shanghai 200062 China; ^4^ Institute for Nanotechnology (INT) Karlsruhe Institute of Technology (KIT) P. O. Box 3640 Karlsruhe 76021 Germany; ^5^ Lehn Institute of Functional Materials (LIFM) School of Chemistry Sun Yat‐Sen University (SYSU) Guangzhou 510275 China

**Keywords:** active metal template, daisy chain, freely gliding, mechanical bond, Π‐stacking

## Abstract

A daisy chain architecture without a preferred low energy arrangement of the mechanically linked components is presented. The molecular design combines a rigid‐rod type oligophenylene ethynylene subunit with an oligoethylene glycol macrocycle that features a bipyridine coordination site. The daisy chain dimer was synthesized via kinetic trapping of the interlocked structure using a Cadiot–Chodkiewicz active metal template reaction. Comparison of the isolated interlocked dimer with its monomeric analogue indicates the presence of a variety of different geometries for the molecular daisy chain. The dynamic sliding motion in the daisy chain is studied by variable temperature UV–vis and nuclear magnetic resonance (NMR) spectroscopy experiments, which point to a highly mobile system even at low temperatures.

## Graphical abstract

A daisy chain compound lacking intercomponent recognition motifs is synthesized and analyzed. As a result of the absence of intercomponent recognition motifs there was also no preferred conformation of the interlocked components observed.

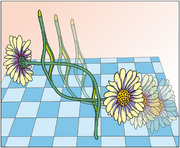

## 1 Introduction

**Figure 1:**
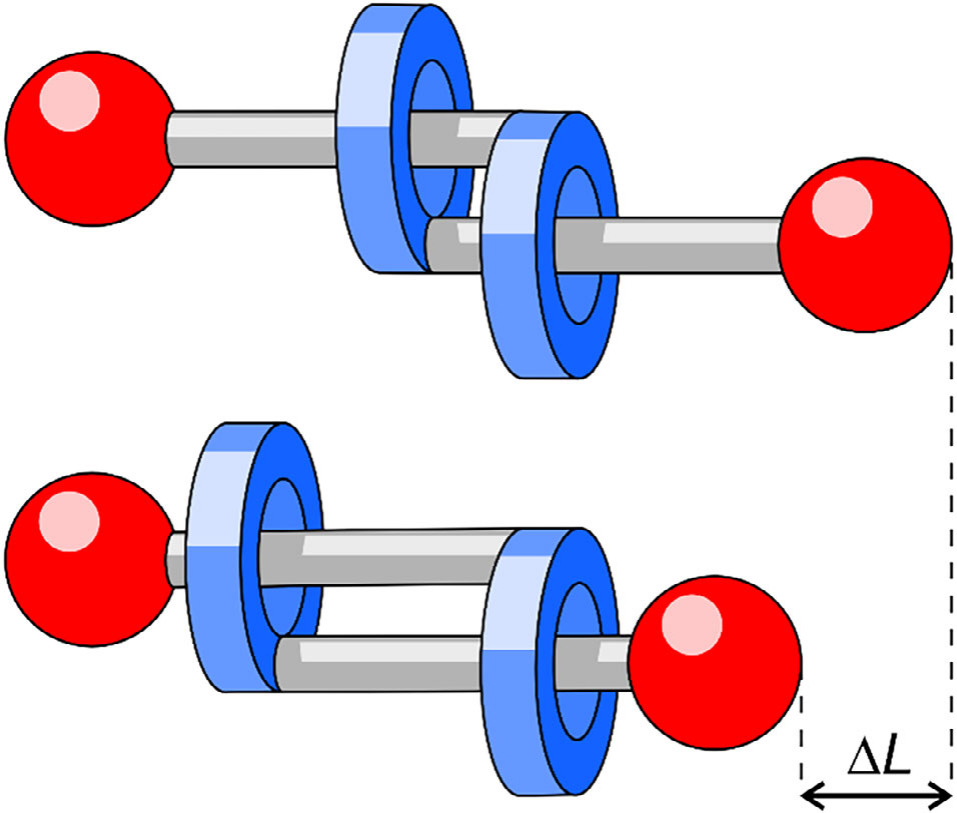
Schematic representation of a molecular daisy chain without strong intercomponent interactions and the possible displacement upon extension and contraction.

Molecular structures featuring components that are linked only mechanically allow the investigation of what are essentially intermolecular interactions within a single molecule.^[^ ^1^, ^2^, ^3^, ^4^ ^]^ The mechanical bond restricts the distance between and orientation of the components, and their position with respect to each other is usually determined by the most favored intra‐ and intercomponent interactions.^[^ ^1^, ^2^, ^3^, ^4^, ^5^, ^6^, ^7^, ^8^, ^9^ ^]^ Such a discrete state is a thermodynamic minimum and is generally referred to as a “station”. The presence of modifiable stations allows the molecule to be switched between different conformations by an external trigger, for example the chemical environment.^[^ ^1^, ^2^, ^3^, ^4^, ^5^, ^6^, ^7^, ^8^, ^9^ ^]^ It is therefore possible to induce and study motion within mechanically interlocked structures by favoring different intercomponent interactions in a controlled manner.^[^ ^2^ ^]^ When the switching between stations is applied to a rotaxane architecture, where a macrocycle is threaded on an axle and stoppered by bulky substituents, the large amplitude motion of the different components with respect to each other can be achieved.^[^ ^1^, ^9^, ^10^, ^11^ ^]^ Applying this shuttling process to a molecular daisy chain (where a singly stoppered axle is terminated by a macrocycle that threads another identical component, as conceptually displayed in Figure 1), the molecular volume can be expanded and contracted by switching between different well defined sites.^[^ ^3^, ^5^, ^6^, ^7^ ^]^ The absence of such stations would allow the linear sliding motion of the different components with respect to each other to have a broad distribution over various geometries and increased conformational freedom.

The synthesis of mechanically interlocked structures, including catenanes, rotaxanes and molecular daisy chains remains challenging. After the pioneering work of Gottfried Schill, synthesizing and isolating a catenane by a covalent template strategy,^[^ ^12^ ^]^ most reported methods rely on the assembly of stable supramolecular complexes that preorganize the precursors and keep them in place during the formation of the mechanical bond.^[^ ^13^, ^14^, ^15^ ^]^ The preorganization is usually achieved using either host‐guest systems,^[^ ^3^, ^4^, ^5^, ^7^, ^9^, ^16^ ^]^ or metal complexes.^[^ ^6^, ^13^, ^17^ ^]^ The assembly of station‐less mechanically interlocked structures lacks these possibilities and is thus more challenging. It has, however, become possible via an active metal template approach,^[^ ^1^, ^2^, ^13^, ^14^, ^15^, ^18^ ^]^ developed in particular for click reactions^[^ ^5^, ^13^, ^14^, ^15^, ^18^ ^]^ and acetylene cross coupling reactions.^[^ ^14^, ^15^, ^19^, ^20^, ^21^ ^]^ This approach relies on the kinetic trapping of the interlocked species.^[^ ^13^, ^14^ ^]^ The successful synthesis of a short and flexible aliphatic daisy chain via active metal template approach was recently reported.^[^ ^2^ ^]^

We are interested in daisy‐chain model compounds as a strategy to mechanically control the proximity of the π‐surfaces of rigid‐rod type subunits. In particular the linear sliding motion of station‐less daisy chain systems as a structural element might enable the observation of π‐stacking in single molecule junctions,^[^ ^22^, ^23^, ^24^, ^25^ ^]^ even with too small π‐overlap to stabilize dimer formation in the junction.

In this study we report the synthesis of a daisy chain dimer consisting of rigid‐rod type axles lacking strong intercomponent binding sites. The synthesis, isolation, and characterization of the mechanically interlocked dimer and the noninterlocked monomer species are discussed. A comparison of both structures allowed features arising from the mechanical architecture to be extracted. In particular, variable temperature UV‐vis and nuclear magnetic resonance (NMR) experiments provided inside into the overlap of the π‐surfaces in the daisy chain dimer.

## 2 Results and Discussion

### 2.1 Design and Retrosynthesis

The synthesis of rotaxanes and catenanes via active metal template acetylene coupling is well established.^[^ ^1^, ^2^, ^14^, ^15^, ^19^, ^20^, ^21^, ^26^ ^]^ The use of this synthetic strategy for the formation of a flexible aliphatic daisy chain was recently reported by Van Raden et al.^[^ ^2^ ^]^ We envisioned our rigid supramolecular structure to include a 1,3‐butadiyne, accessible via active metal template synthesis, which required the macrocycle to include a bipyridine ligand.^[^ ^1^, ^14^ ^]^ To introduce selectivity in the acetylene coupling we relied on the Cadiot–Chodkiewicz reaction, which enables the formation of asymmetric 1,3‐butadiynes. The resulting target structure includes a polyethylene glycol‐based ring including the bipyridine for the active metal template acetylene coupling^[^ ^1^, ^14^ ^]^ and a longer rigid oligo phenylene ethynylene (OPE) backbone.

**Scheme 1:**
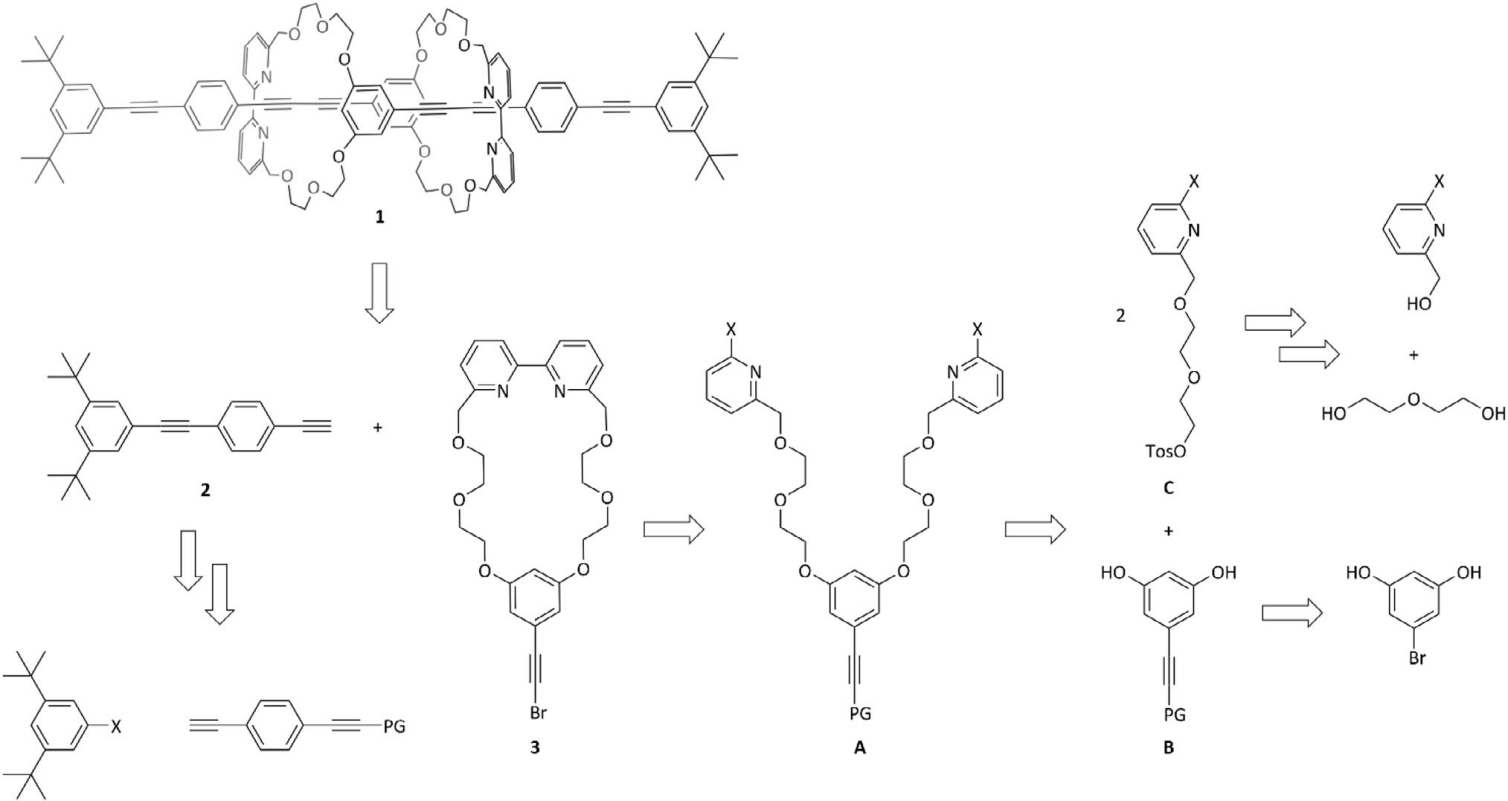
Retrosynthetic analysis of molecular daisy chain **1**.

The retrosynthetic strategy of the molecular daisy chain **1** is reported in Scheme 1. The key step in the synthesis of the designed structure was envisioned to be the active metal templated Cadiot–Chodkiewicz reaction toward **1**.^[^ ^1^, ^2^ ^]^ The resulting precursors are the OPE axle **2**, including two *tert*‐butyl groups serving as stopper units, and the symmetric macrocycle **3**. The retrosynthetic analysis of **2** includes a series of Sonogashira–Hagihara cross‐coupling reactions and acetylene deprotections. The design of the macrocycle includes a bipyridine for the active metal template reaction, a phenylacetylene unit for the rigid backbone and a flexible polyethylene glycol (PEG) linker to connect the two units. To facilitate the synthesis and characterization, the macrocycle was designed to be symmetric. The size of the ring is determined by the length of the PEG chain, for which we decided to use a diethylene glycol spacer. Based on the literature reported high yielding pyridine homocoupling macrocyclization,^[^ ^27^ ^]^ the bipyridine of the macrocycle was envisioned to be a good disconnection point for the synthesis of **3**, resulting in precursor **A**. The symmetric derivative **A** was planned to be accessible via a twofold S_N_2 reaction of **B** with **C**. While **B** was envisioned to be synthesized via Sonogashira–Hagihara cross‐coupling reaction of commercially available 5‐bromoresorcinol, **C** should be accessible via a series of tosylations and Williamson etherifications, requiring the asymmetrization of the diethylene glycol.

### 2.2 Synthesis and Characterization

**Scheme 2:**
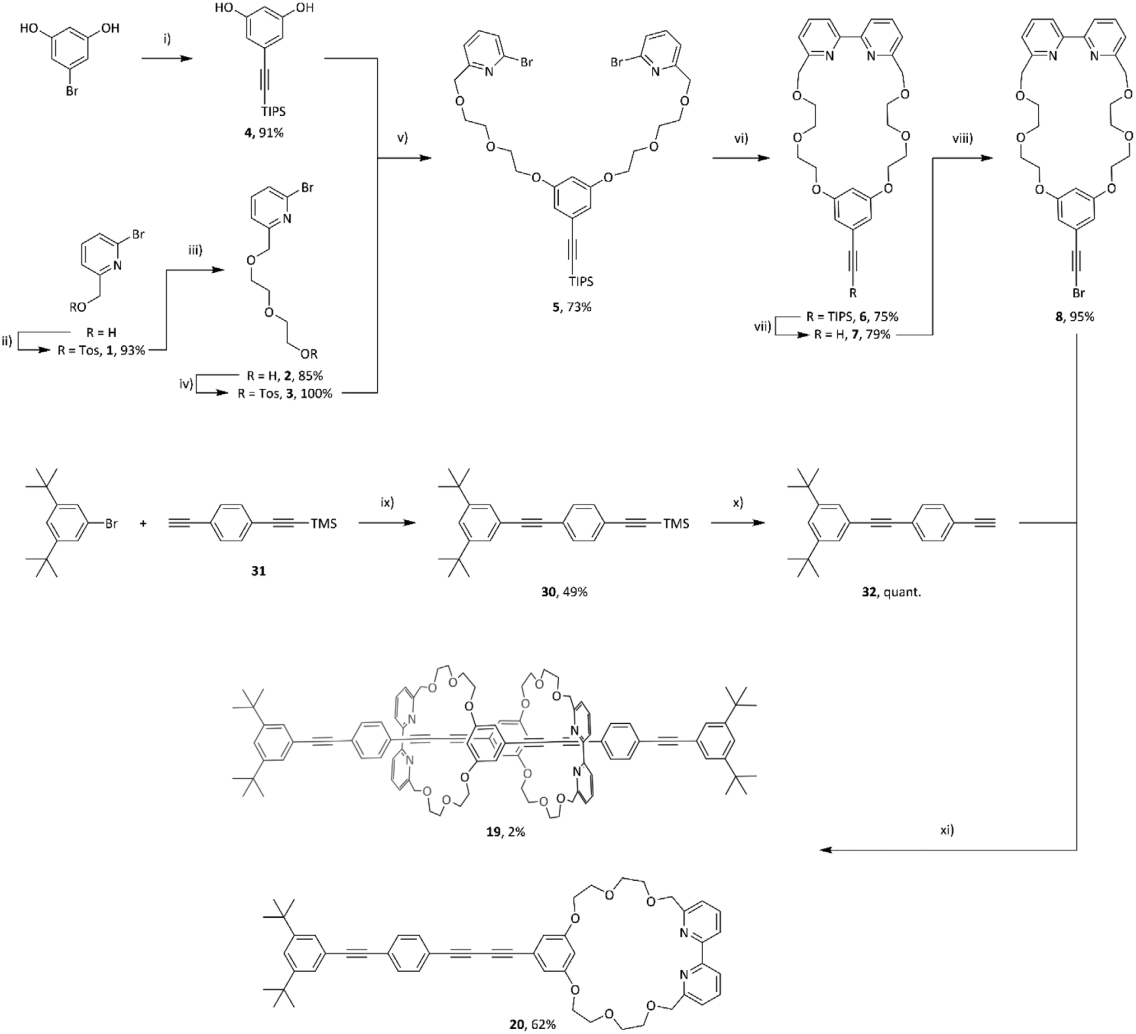
Synthesis of [c2] daisy chain **1**. Reagents and conditions: i) triisopropylsilyl acetylene, Pd(PPh_3_)_2_Cl_2_, CuI, Et_3_N, 70 °C, and 64 hours; ii) TosCl, NaOH, THF, H_2_O, 0 °C to rt, 4 hours; iii) diethylene glycol, NaH, THF, 60 °C, 16 hours; iv) TosCl, Ag_2_O, KI, CH_2_Cl_2_, rt, 16 hours; v) Cs_2_CO_3_, MeCN, reflux, 16 hours; vi) Ni(PPh_3_)_2_Br_2_, PPh_3_, Mn, Et_4_NI, DMF, 50 °C, 4 hours; vii) TBAF, THF, rt, 2 hours; (viii) K_2_CO_3_, 18‐crown‐6, CBr_4_, THF: MeOH (1:1), rt, 3 hours; ix) Pd(PPh_3_)_2_Cl_2_, CuI, THF: Et_3_N (3:1), 70 °C, 16 hours; x) K_2_CO_3_, MeOH: THF (1:1), rt, 4 hours; xi) Cu(MeCN)_4_PF_6_, diisopropylethylamine, CHCl_3_, EtOH (3:2, 7 mM), 40 °C, 72 hours.

The synthesis of the circular daisy chain **1** and the monomer **4** is shown in Scheme 2 and described in detail in the following. The Sonogashira–Hagihara cross coupling reaction on the resorcinol derivative allowed to isolate **5** in a yield of 91% with a reaction time of 64 hours. Literature reports^[^ ^28^ ^]^ allowed to isolate **6** in a yield of 93%. After optimization, the Williamson etherification of diethylene glycol (6.0 equiv) with tosylate **6** (1.0 equiv) and sodium hydride (NaH) in tetrahydrofuran (THF) at 60 °C proceeded in a yield of 85%. The tosylation of **7** was most successful when performed with silver oxide (Ag_2_O) in dichloromethane (CH_2_Cl_2_), resulting in a quantitative yield of **8**. For the following S_N_2 reaction cesium carbonate (Cs_2_CO_3_) was employed as a base and **9** was isolated in a yield of 73%. When subjecting **9** to the literature, reported bipyridine homocoupling conditions^[^ ^27^ ^]^ **10** could be isolated only in a yield of 14%, while the main isolated species was the starting material **9**. We therefore assumed that the amount of activated nickel was not enough to allow for completion. When significantly increasing the amounts of the required reagents dibromo bis(triphenylphosphin)nickel(II) (Ni(PPh_3_)_2_Br_2_, 5.0 equiv), triphenyl phosphine (PPh_3_, 10 equiv), manganese (Mn, 50 equiv), and tetraethyl ammonium iodide (NEt_4_I, 5.0 equiv) **10** was isolated in a yield of 75%. The triisopropylsilyl acetylene deprotection afforded **11** in a yield of 79%. When employing standard acetylene bromination conditions using silver nitrate (AgNO_2_) and *N*‐bromosuccinimide to **11**, overbromination was observed. A yield of 95% for **3** was obtained when mild literature reported conditions with potassium carbonate (K_2_CO_3_), 18‐crown‐6 and carbon tetrabromide (CBr_4_)^[^ ^29^ ^]^ were applied to **11**. The optimized reaction conditions allowed to isolate the macrocycle **3** in an overall yield of 32% over 7 steps. The OPE backbone was synthesized in two steps. The Sonogashira–Hagihara cross‐coupling reaction of 1‐bromo‐3,5‐di‐*tert*‐butylbenzene with literature known **12** ^8^ allowed to isolate **13** in a yield of 49%. While conversion was complete, the purification turned out to be challenging due to the non‐polarity of the desired structure, resulting in a significant loss of crude material upon isolation. Acetylene deprotection of **13** to **2** proceeded quantitatively. For the key active metal templated Cadiot–Chodkiewicz reaction of **2** with **3** toward the formation of the molecular daisy chain **1**, literature reported conditions employing tetrakis(acetonitrile)copper(I) hexafluorophosphate (Cu(MeCN)_6_PF_6_) as a copper source and diisopropylethylamine (DIPEA) were chosen.^[^ ^2^ ^]^ First attempts in THF (5 mM) led to the isolation of **4** in a modest yield of 14%. Based on the reported findings of Berna et al.^[^ ^1^ ^]^ we performed the reaction at higher concentrations (45 mM), which increased the yield of **4** to 22%. The poor solubility of the starting materials resulted in the addition of chloroform (CHCl_3_) to the reaction mixture in the next attempt in order to obtain the desired daisy chain **1**. When employing a solvent mixture of THF and CHCl_3_ (3.5:1, 28 mM) or CHCl_3_ and ethanol (EtOH, 3:2, 17.5 mM) with Cu(MeCN)_6_PF_6_ (0.95 equiv) and DIPEA (2 equiv) the desired molecular daisy chain **1** was isolated in trace amounts, while the nonmechanically interlocked species **4** was the main product. Further decrease in concentration (CHCl_3_:EtOH, 3:2, and 7 mM) with a sub‐equimolar amount of Cu(MeCN)_6_PF_6_ (0.75 equiv) allowed **1** to be isolated in a yield of 2% after purification by silica gel column chromatography followed by precipitation with methanol from dichloromethane. Non‐interlocked monomeric **4** was isolated in 62% yield after the same purification procedure.

**Figure 2:**
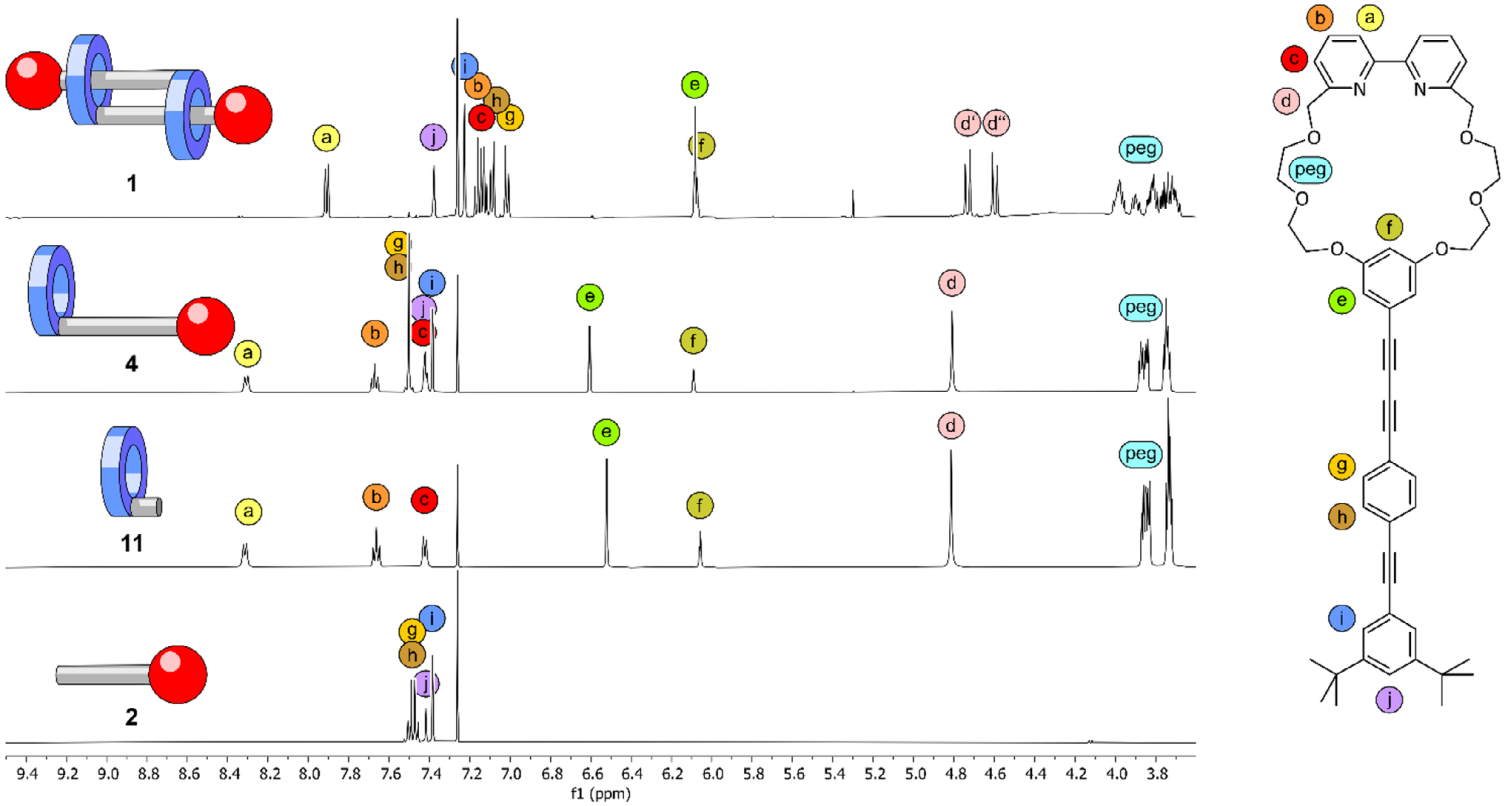
Stack plot of the ^1^H NMR spectra above 3.6 ppm (500 MHz, chloroform‐d, 298 K) of [c2] daisy chain **1** and its structural components **2**, **11**, and **4**.

The identity and purity of the reported structures were corroborated by high‐resolution electrospray ionization mass spectrometry (HRMS), proton nuclear magnetic resonance (^1^H‐NMR), and proton‐decoupled carbon NMR (^13^C{^1^H} NMR) spectroscopy. Detailed experimental procedures and all analytical data can be found in the Supporting Information. The full characterization with state‐of‐the art NMR spectroscopy of **1**, **2**, **3,** and **4** allowed to assign all signals as shown in Figure 2. Furthermore, the optical properties of **1**, **2**, **4,** and **11** were investigated. Due to its reported light sensitivity,^[^ ^30^, ^31^ ^]^ we refrained from investigating the optical properties of the brominated acetylene **3**. The inspection of the optical properties enables some preliminary conclusions concerning the variety of arrangements between both mechanically interlinked subunits of the [c2] daisy chain super‐structure and thus, a more detailed discussion will follow.

The ^1^H‐NMR signals of the daisy chain monomer **4** are more or less the sum of the signals of the macrocycle **3** and the OPE rod with stopper unit **2**, when neglecting the singlet of the free acetylene at 3.17 ppm. The few spectral changes upon formation of **4** are the signals of the protons (g) and (h) of the axle, which merge to one overlapping signal, and the downfield shift of proton (e) of 0.1 ppm compared to the macrocycle **3**. The formation of the interlocked species **1** leads to a ^1^H NMR spectrum with the same number of aromatic signals as the monomer **4**, while showing substantial changes in some chemical shifts. This points at a structure of high symmetry like the targeted interlocked [c2] daisy chain or any other cyclic [cn] daisy chain oligomer. It is noteworthy that linear [an] daisy chain oligomers have lower symmetry and thus, ^1^H‐NMR spectra with more signals would be expected.

The confirmation that indeed the mechanically interlocked [c2] daisy chain **1** has been obtained was provided by high‐resolution mass‐spectrometry. The molecular weight of the isolated species agrees with 1605.8007 m/z perfectly with the protonated mechanically interlocked target structure (1605.8037 m/z for C_104_H_109_N_4_O_12_^+^). The cluster analysis (see Supporting Information page SI–52) further corroborates that exclusively singly charged species were responsible for the MS signal and consequently, the elemental composition of the target structure. The mechanically interlocked [c2] daisy chain **1** is the only dimeric arrangement satisfying both, the analyzed elemental composition and the high symmetry observed in the ^1^H‐NMR spectra.

Closer inspection of the ^1^H‐NMR spectra of **1** reveals a pronounced upfield shifts for almost all signals with only the protons (f) and (j) as exceptions. Those protons (f and j) are located at the end of the rigid backbone and are therefore less influenced by the proximity of the mechanically fixed macrocycle of the neighboring subunit. The significant shift of all other protons (e.g., 0.5 ppm for protons (b), (e), and (h), 0.4 ppm for (g) and (a), 0.3 ppm for (c), and 0.15 ppm for (i)) can be attributed to the formation of the mechanically interlocked species, which results in the spatial proximity of the phenylenes of the two axles in respect to each other and thus, their magnetic anisotropy results in the observed shifts for **1** compared to **4**, **3,** and **2**. Striking evidence for the formation of the interlocked structure is also the diastereotopic splitting of the benzylic protons (d) in **1**. The splitting results from the position of the protons toward either the inside or the outside of the stacked double ring arrangement in the [c2] daisy chain superstructure. 2D ROESY NMR experiments and the resulting cross peaks (see Supporting Information, synthesis and characterization of **1**) allowed to conclude, that (d’) is oriented toward the inside of the stacked double ring formed by the daisy chain dimer, while (d’’) is pointing toward the outside, showing coupling signals to the *tert*‐butyl groups of the stopper unit.

A full assignment of the PEG protons (see Supporting Information, synthesis and characterization of **1**) revealed diastereotopic splitting also for those protons. Furthermore, an increased dispersion of the signals of the PEG chain can be observed upon formation of the [c2] daisy chain. This can be attributed to the difference of the chemical environment inside the PEG macrocycle, changing from solvent molecules in **3** and **4** to the rigid OPE axle in **1**, hindering the rotation of the PEG chain in **1**.

Tandem mass spectrometry experiments not only corroborated the presence of a mechanically interlocked structure, but also enabled statements about its stability. Up to a collision energy of 35 keV exclusively the intact molecular mass of the [c2] daisy chain **1** was observed, pointing at the considerable stability of the mechanically interlocked species. Above that threshold value for the collision energy various fragments were detected. It thus seems that the mechanically interlocked species goes to pieces in a rather uncontrolled manner and that there is no particular labile bond breaking preferentially.

### 2.3 Optical Analyses

**Figure 3:**
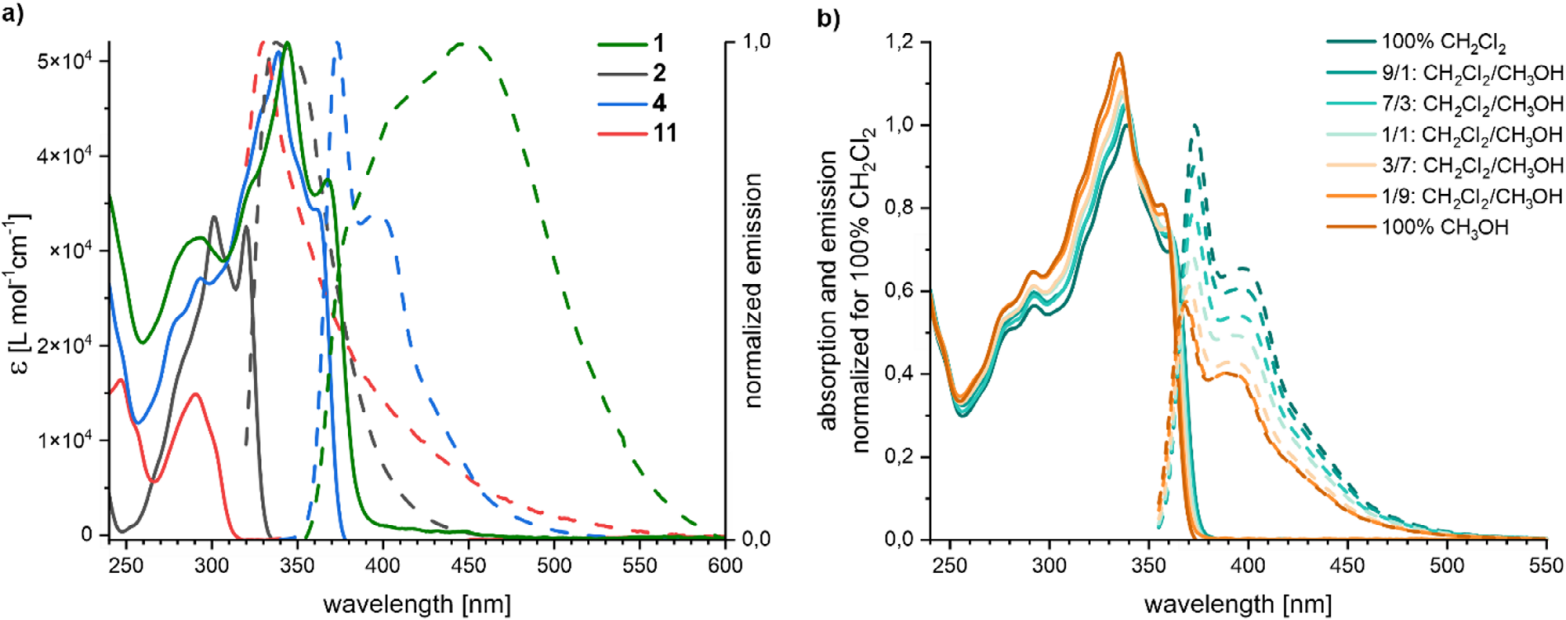
(a) Absorption (solid line) and emission (dashed line) of **1** (green), **4** (blue), **11** (red), and **2** (black) in dichloromethane at 20 °C. The absorption spectrum of **1** is normalized. (b) Absorption (solid line) and emission (dashed line) of **4** in different dichloromethane to methanol ratios measured at 20 °C. The spectra are displayed in relationship to the first measurement performed in dichloromethane, which is normalized.

The absorption and emission spectra of **1**, **2**, **4**, and **11** are displayed in Figure 3a and summarized in Table 1. Compared to the precursors **2** and **11**, a pronounced bathochromic shift is observed upon formation of the rigid rod subunit for both, the monomer **4** and the daisy chain **1**. This shift is rationalized by the increase of the conjugated system. The absorption spectra of **1** and **4** display the signature of both, the bipyridine comprising macrocycle between 260 and 305 nm,^[^ ^32^, ^33^ ^]^ and the conjugated rigid‐rod type structure above 305 nm. Of particular interest is the detailed comparison of the spectra of **1** and **4**. Upon formation of the mechanical bond a red shift for all maxima (see Figure 3a and Table 1) is observed. These bathochromic shifts are indicative for increased conjugation, which may arise from intermolecular π–π stacking interactions in the superstructure **1**. The comparison further reveals a decrease in the vibronic fine structure in the absorption spectra of **1** compared to **4**. The loss of vibronic fine structure in the superstructure points at the presence of a variety of geometries, arising from the continuous mechanical dynamic motion of the interlocked species **1**. The hypothesized sliding motion in **1** is furthermore supported with its emission spectrum, which is significantly red shifted compared to **4** and shows a very broad peak with almost no vibronic resolution and a large *Stokes* shift. A very comparable behavior was recently reported for the emission of a molecular daisy chain.^[^ ^2^ ^]^

**Table 1:** Optical properties of **11**, **2**, **4**, and **1** in dichloromethane at 20 °C.

Compound	*λ*_max_ [nm] (ε_λmax_ [L mol^−1^ cm^−1^])	*λ*_em_ (excitation) [nm]	Φ_f_ (excitation [nm])
**11**	291 (14.9·10^3^)	330 (300)	Too low to be measured (300)
**2**	301 (33.6·10^3^) 320 (32.6·10^3^)	335, 337 (300)	81.4% (300)
**4**	293 (27.1·10^3^) 339 (51.0·10^3^) 361 (34.4·10^3^)	373, 396 (320)	10.4% (335)
**1**	294, 324(shoulder), 344, 368	375 (shoulder), 410 (shoulder), 450 (335)	7.5% (335)

To what extent the mechanical fixation in **1** impels the π‐stacking between the rigid‐rod type subunits in the superstructure was investigated by aggregation studies with **4**. During its isolation, the monomer **4** was dissolved in dichloromethane and aggregated or, depending on its concentration, even precipitated upon addition of methanol. Absorption and emission spectra of **4** were thus recorded in different volume ratios of dichloromethane and methanol as displayed in Figure 3b. With an increasing fraction of methanol, the absorption peak increases while the emission signal decreases. The vibronic fine structure is maintained and a slight blue shift of the absorption maxima is observed. Overall, the spectral changes can be rationalized as solvophobic effects and no signs of intermolecular interactions like aggregation significantly altering the optical properties of the monomer **4** were observed. The bathochromic shift of the absorption maxima as well as the substantial broadening of the emission signal was thus exclusively recorded for the mechanically fixed dimer in the superstructure **1** and could not be mimicked by aggregation of **4**.

The dynamic of the superstructure **1** was investigated by variable temperature UV–vis experiments between 10 °C and 80 °C in 1,2‐dichloroethane (see Supporting Information page SI–56). At all temperatures, spectra with comparable poor vibronic resolution were recorded pointing at a large variety of populated states at all investigated temperatures. Interestingly, the largest variety in intensity is observed for the spectral region attributed to the rigid backbone (above 310 nm), suggesting that thermal energy impacts the relative populations of different geometries and positions of the OPE axles in the mechanically fixed dimer. Similar aggregation studies as above described for **4** were performed with the daisy chain **1** (see Supporting Information page SI–57). Only very minor changes in the absorption spectra of **1** were recorded. However, a similar blue shift with an increasing volume ratio of methanol as for **4** was observed. In summary, it seems that the solvent polarity has no detectable influence on the distribution of the arrangements of both subunits in the superstructure.

In summary, the optical analyses suggest that both subunits of the daisy chain **1** have a large distribution over different geometries and that there is no favored arrangement in the super‐structure. The large variety of different geometries of **1** is interpreted as being indicative for free sliding of the rigid‐rods’ π‐surfaces on each other, which was found to be more sensitive to temperature than to solvent polarity.

### 2.4 Analysis of the Free Sliding with NMR Experiments

**Figure 4:**
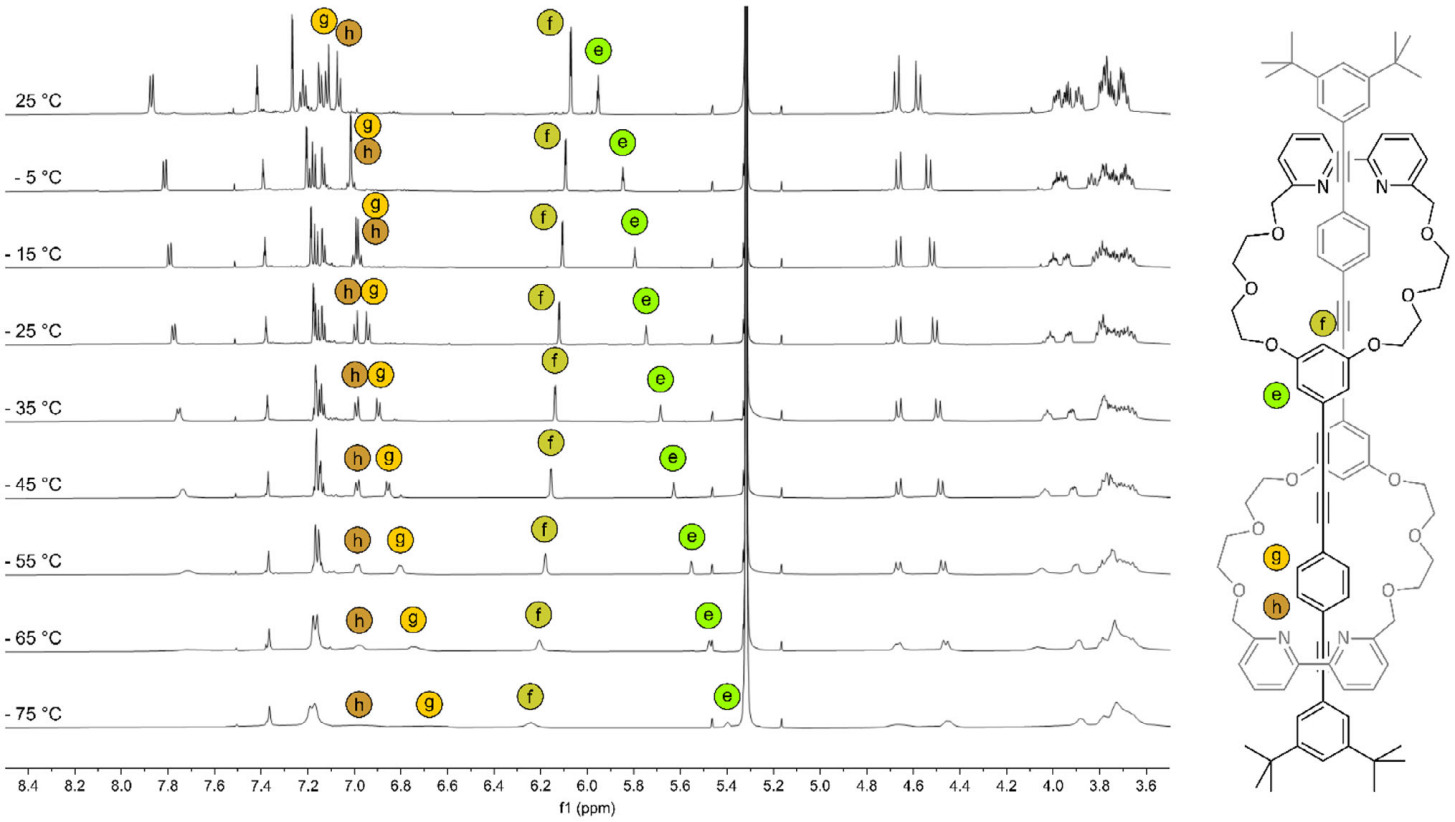
Variable temperature ^1^H NMR (600 MHz, dichlorometane‐d2) of **1**.

Variable temperature NMR experiments of **1** in CD_2_Cl_2_ were performed to further study the sliding of the two π surfaces with respect to each other. ^1^H NMR spectra were recorded every 10 °C from −75 °C to −5 °C, and one spectrum was recorded at 25 °C, as shown in Figure 4. All signals shift upon decreasing the temperature, however, the most pronounced up‐field shift can be observed for proton (e). The signals of protons (g) and (h) overlap at 5 °C, while two clear signals, with inverted order, can be observed upon further cooling (starting from −25 °C). Overall, the variable temperature ^1^H‐NMR experiments reveal that one sharp set of signals can be observed until −75 °C, indicating that no predominant conformation was observed upon cooling but rather a fast exchange between the extended and the contracted form (see below). The faster broadening of the bipyridine signals upon decreasing the temperature (between −35 °C and −55 °C) compared to other aromatic protons of the rigid OPE axle suggests that the motion of the bipyridine is frozen into different noninterconvertible geometries before the sliding motion of the daisy chain is restricted.

**Figure 5:**
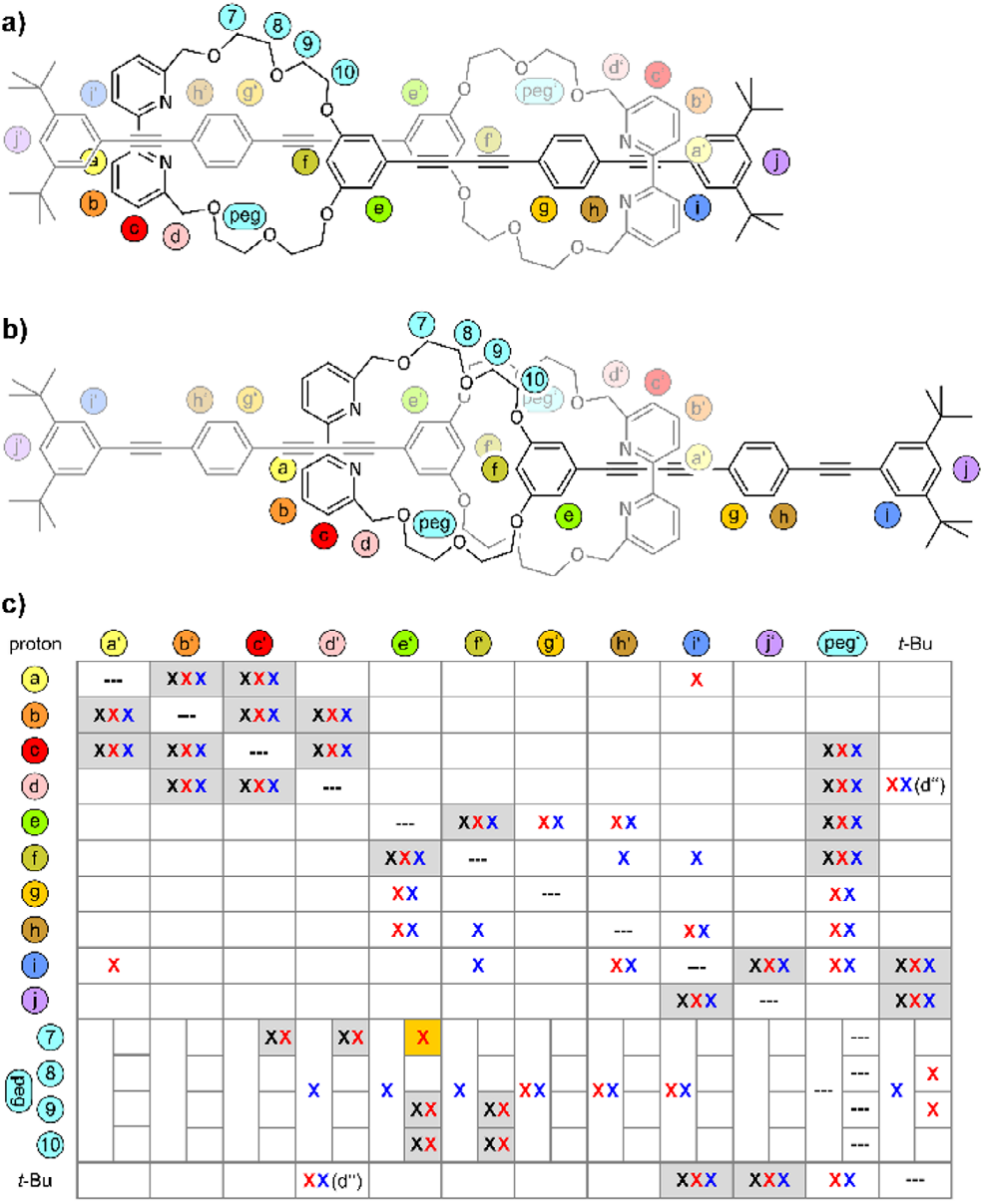
Representation of the (5a) contracted and (5b) extended molecular daisy chain with letters and numbers labelling the positions used in the table. Table (5c) of the ROESY cross peaks between different protons. The black crosses belong to the measured monomer **4** in CD_2_Cl_2_ at 25 °C (note: the apostrophes of the labels only make sense for **1** and should be ignored in the case of **4**). The red crosses belong to the daisy chain **1** in CD_2_Cl_2_ at 25 °C. The blue crosses belong to the daisy chain **1** in CD_2_Cl_2_ at −35 °C.

To further confirm the presence of the mechanically interlocked species and to analyze the presence of both, the contracted and the extended form of the molecular daisy chain **1**, ROESY NMR experiments of **1** were recorded and compared with the signals observed for the monomer **4** (see Supporting Information Figure 1). The detected ROE cross peaks are summarized in the table of Figure 5c. The ROESY spectrum of **4** displays all signals arising from the intramolecular proximity of subunits and are marked with black crosses in the table. As these intramolecular ROESY signals are not helpful to analyze dynamic features of the mechanically interlocked daisy chain **1**, these cells are additionally indicated by a grey background. In addition to these intramolecular signals, the ROESY spectrum of **1** displayed inter‐molecular signals corroborating the proximity of both molecular rods in the mechanically fixed daisy chain. The signals detected in the experiment at room temperature are indicated with red crosses, while the ones recorded at −35 °C are shown as blue crosses in the table of Figure 5c. Particularly intense are the cross peaks between the *tert*‐butyl group and the PEG‐chain as well as proton (d’’), which was previously attributed to the proton facing toward the outside of the mechanically interlocked ring. These signals can be attributed to a contracted form of the molecular daisy chain (see Figure 5a).

The PEG protons show cross peaks to a large number of signals in **1**. While some of them arise from intramolecular couplings, the ones to protons (g–i), and the *tert*‐butyl group can be attributed to the through‐space interaction of the daisy chain. Interestingly, the protons (g–i) are distributed along the rigid‐rod structure suggesting that the PEG macrocycle is sliding along the rigid‐rod axle of the pairing molecules in the daisy chain. Particularly interesting is the cross peak between protons (e) and 7 of the PEG‐chain, which is absent in **4**, as this signal confirms the presence of the extended daisy chain form (see Figure 5b). The signal is highlighted by a dark yellow background in Figure 5c. Further ROESY peaks between the aromatic protons can be observed. These signals detected at room temperature and at −35 °C were attributed to the contracted daisy chain form. Interestingly, some minor peaks were only observed at one temperature, such as the cross peak of (a) with (i) at room temperature or the ones of (f) with (h) and (i) at −35 °C. At first glance it seems that there are more cross peaks which can be attributed to the contracted form. However, most of the expected peaks for the extended form can already be observed as intramolecular ROESY signals with the monomer **4**, and thus these signals are not only ignored as unambiguous proof for the mechanically interlocked species but also are missing to estimate the presence of the extended daisy chain.

Overall, the NMR analyses suggest a dynamic daisy chain present in a variety of degrees of extension in CD_2_Cl_2_ at the measured temperatures (25 °C and −35 °C). The observation of a single set of sharp signals in the ^1^H‐NMR spectrum points at fast interconversion between the differently extended forms quicker than the NMR timescale. Albite CH···O interaction being possible, the variable temperature NMR experiments show that these interactions are either neglectable or do not lead to a preferred conformation of the mechanically interlocked species. These findings agree with the UV‐vis analyses discussed before and support the hypothesized free sliding of the rigid‐rod π‐surfaces paired in the mechanically fixed daisy chain. In other words, in case there is a favored position of both mechanically interlocked molecules, its thermodynamic stabilization is so little that it can be overcome at temperatures above −75 °C.

## 3 Conclusion

In spite of the lack of stabilizing intercomponent interactions, a daisy chain dimer with rigid‐rod type axles and oligoethylene glycol macrocycles was realized. The successful assembly is based on an active metal template Cadiot–Chodkiewicz reaction, with bipyridine subunits in the macrocycle coordinating the copper ion during the assembly of the axles. The identity of the [c2] daisy chain **1** is unambiguously demonstrated by its analytical data, in particular by the combination of its ^1^H‐NMR spectrum and mass recorded by atmospheric‐pressure chemical ionization high‐resolution mass‐spectrometry. Noteworthy in the ^1^H‐NMR spectrum is the low number of signals, as expected for the high symmetry structure, and the diastereotopic splitting of the benzylic protons which become fixed inside or outside in the threaded superstructure. The intention to arrange two rigid‐rod type π‐systems freely gliding on top of each other in the daisy‐chain architecture appears to be successful, as the low vibronic resolution of **1** in absorption and emission result from a large number of different arrangements of both molecules paired in the [c2] daisy chain. These spectral features arise from the close proximity of both π‐surfaces and are only observed for the daisy chain; they cannot be simulated by aggregation of monomer **4**. Cross peaks in the ROESY NMR spectrum of **1** further corroborate the rich variety of geometries present, with variable temperature NMR experiments indicating dynamic sliding even at low temperature (−75 °C) where the torsional motion of the bipyridine subunits in the macrocycles are frozen.

The realization of a [c2] daisy chain with freely gliding rigid axles is appealing as a mechano‐sensitive subunit for mechanically controlled single molecule experiments. We are currently working toward the integration of the subunit in such experiments.

## Supporting Information

The Supporting Information comprises ROESY NMR spectra, the synthetic protocols, the characterization data and the spectra of all new compounds, variable temperature and aggregation UV–vis studies of the [c2] daisy chain **1**. The authors have cited references from the main manuscript within the Supporting Information.^[^ ^8^, ^27^, ^28^ ^]^

## Conflict of Interests

The authors declare no conflict of interest.

## Supporting information



Supporting Information

## Data Availability

The data that support the findings of this study are available in the supplementary material of this article.
