# Supplementary material for: Synthesis of a Station‐Less Molecular Daisy Chain
Source: Chemistry. 2025 May 12;31(33):e202501369. doi: 10.1002/chem.202501369 (PMC12160958; doi:10.1002/chem.202501369)
Supplement: Supplementary file 1 — Supporting Information [file CHEM-31-e202501369-s001.pdf]

Supporting Information

Synthesis of a Station-less Molecular Daisy Chain

Charlotte Kress,[a,b] Daniel Häussinger,[a] David A. Leigh,[b,c] Marcel Mayor[a,d,e]

[a] C. Kress, Prof. Dr. D. Häussinger, Prof. Dr. M. Mayor
Department of Chemistry
University of Basel
St. Johanns-Ring 19, 4056 Basel, Switzerland
E-mail: marcel.mayor@unibas.ch

[b] C. Kress, Prof. Dr. D. A. Leigh
Department of Chemistry
The University of Manchester
Oxford Road, Manchester, M13 9PL, United Kingdom
E-mail: david.leigh@manchester.ac.uk

[c] Prof. Dr. D. A. Leigh
School of Chemistry and Molecular Engineering
East China Normal University
Shanghai, 200062, China

[d] Prof. Dr. M. Mayor
Institute for Nanotechnology (INT)
Karlsruhe Institute of Technology (KIT)
P. O. Box 3640, 76021 Karlsruhe, Germany

[e] Prof. Dr. M. Mayor
Lehn Institute of Functional Materials (LIFM)
School of Chemistry
Sun Yat-Sen University (SYSU), 510275 Guangzhou, China

Table of Contents

Figures of the Supporting Information 3

General 4

Synthesis - Overview 5

Synthesis - Experimental Procedures and Characterization 6

Variable Temperature UV-Vis Study of the Daisy Chain **1** 56

Aggregation Studies with the Daisy Chain **1** 57

References 58

Figures of the Supporting Information

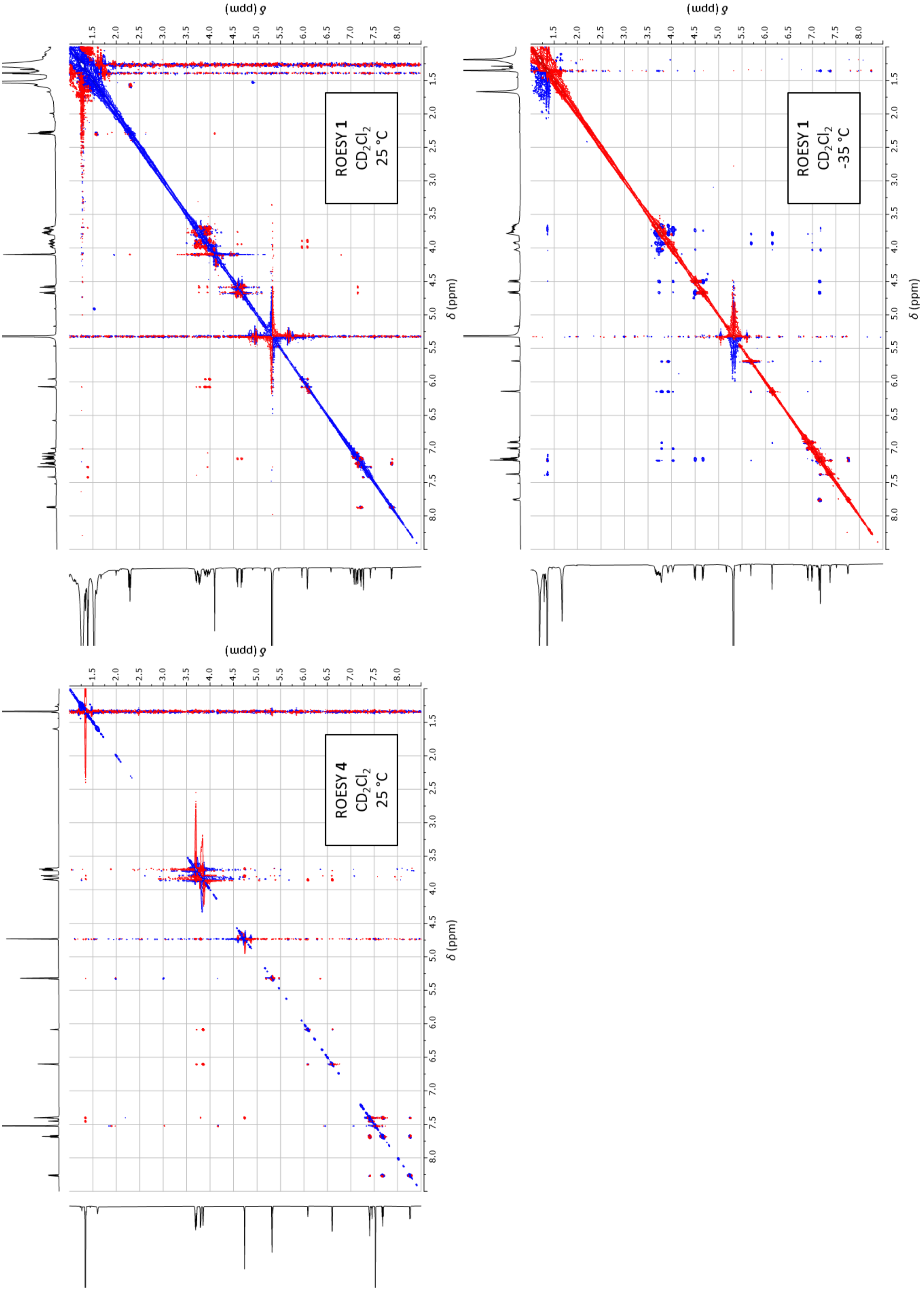

Figure 1 ROESY NMR spectra (600 MHz) of **1** and **4** in dichlorometane-d_2 and different temperatures for **1** as stated in the graphs.

General

Reagents and Solvents

All commercially available compounds were purchased from Sigma-Aldrich, Acros, Apollo Scientific, Alfa Aesar, Fluorochem and TCI and used without further purification. Anhydrous solvents were purchased from Sigma- Aldrich and stored over moleculear sieves (4 Å) or were obtained by passing the solvent through an activated alumina column on a Phoenix SDS (solvent drying system; JC Meyer Solvent Systems, CA, USA).

Analytics and Instruments

TLC was performed with on silica gel plates (0.25 mm thick, 60 F254, Merck, Germany) or glass plates purchased from Merk. A UV-lamp at 254 nm was used for observation.

Column chromatography was performed on silica gel P60 (40−63 µm) from Silicycle or Sigma Aldrich, and the solvents were technical or HPLC grade.

NMR experiments were performed on Bruker Avance III NMR spectrometers operating at 250, 400, 500, or 600 MHz proton frequencies. The chemical shifts (δ) are reported in parts per million (ppm) relative to tetramethylsilane (TMS) and were calibrated using TMS or a residual solvent peak, the J values are given in Hertz (Hz). To describe the proton signals and their pattern, the following abbreviations are used: singlet (s), doublet (d), triplet (t), multiplet (m), apparent triplet (app. t), doublet of doublet (dd).

High resolution electron spray ionization time-of-flight mass spectrometry (HRMS) was performed on a maX-isTM4G instrument from Bruker or was carried out at the Mass Spectrometry Service, Department of Chemistry, University of Manchester.

UV/vis spectroscopy was performed on a JASCO V-770 Spectrophotometer at the stated temperatures. Fluorescence measurements were performed on a Jasco FP-8600 spectrometer (nitrogen purged system). Quantum yield measurements were performed with an absolute photoluminescence quantum yield spectrometer C11347 Quantaurus-QY (Hamamatsu).

IR spectroscopy was performed on a IRTracer-100 Fourier Transform Infrared Spectrometer, Shimadzu.

Synthesis - Overview

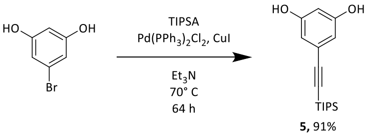

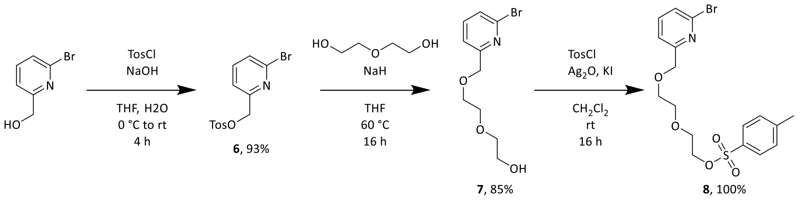

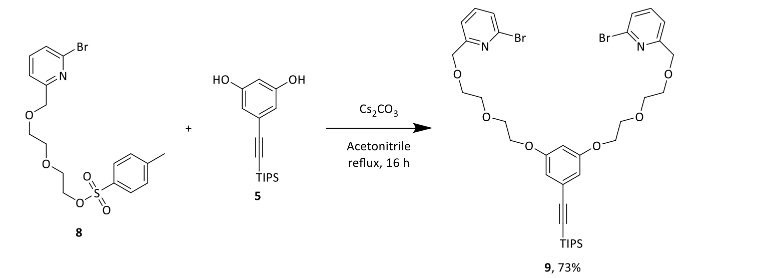

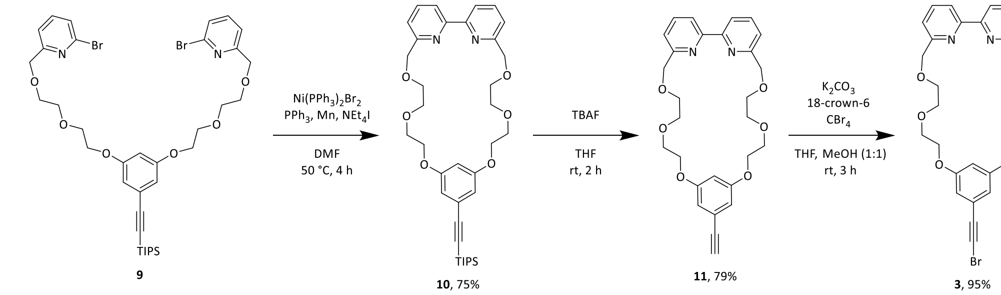

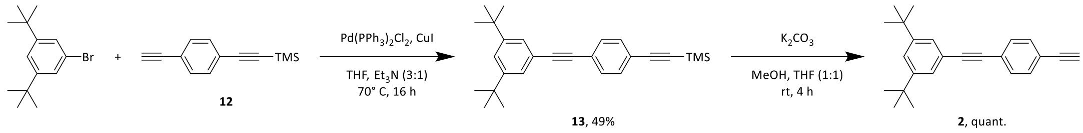

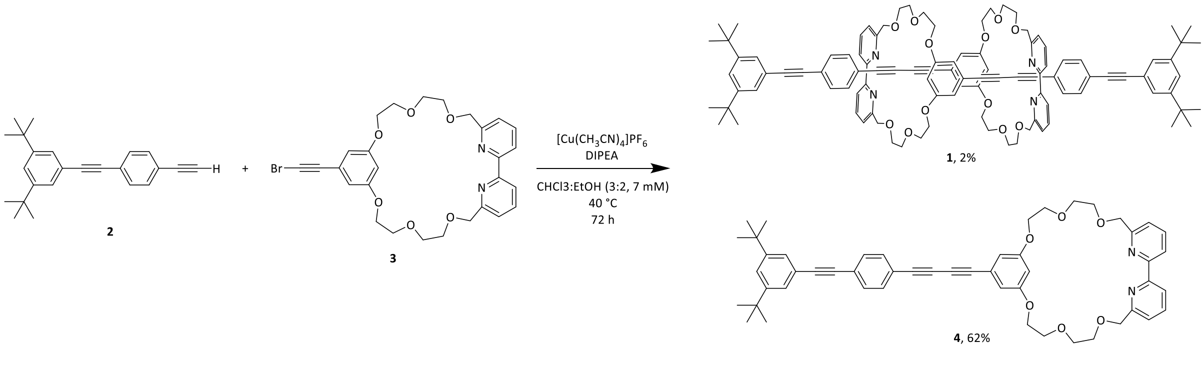

Synthesis - Experimental Procedures and Characterization

Synthesis of **5**

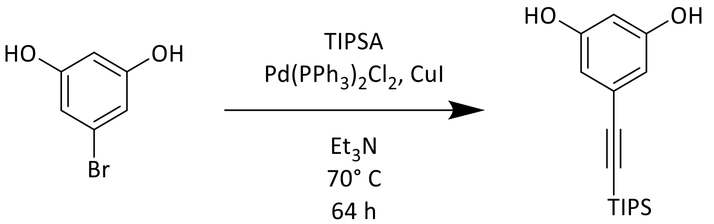

5, 91%

5-Bromoresorcinol (1000 mg, 5.29 mmol, 1.0 equiv.) was added to a pressure tube and dissolved in Et_3N (20 mL). The mixture was degassed by purging with argon for 10 minutes and then copper(I) iodide (50.4 mg, 265 µmol, 0.05 equiv.) and bis(triphenylphosphine) palladium(II) dichloride (186 mg, 265 µmol, 0.05 equiv.) were added. After purging with argon for 5 more minutes (triisopropylsilyl)acetylene (2890 mg, 15.9 mmol, 3.0 equiv.) was added and the reaction mixture was placed into the preheated heating bath at 70°C for 64 h. The crude reaction mixture was filtered over celite and eluted with EtOAc and concentrated *in vacuo*. **5** (1396 mg, 5.29 mmol, 91%) was obtained as a pale orange solid after purification by column chromatography (SiO_2, petroleum ether (40 – 60 °C):acetone (3:1)).

^{1}H-NMR (500 MHz, Chloroform-*d*, 298 K, δ/ppm 6.54 (d, $^4J_{HH}$ = 2.3 Hz, 2H), 6.34 (t, $^4J_{HH}$ = 2.3 Hz, 1H), 5.17 (s, 2H), 1.26 (t, $^4J_{HH}$ = 7.1 Hz, 3H), 1.11 (s, 18H).

^{13}C{^{1}H}-NMR (126 MHz, Chloroform-*d*, 298 K, δ/ppm): 156.7, 125.4, 111.8, 106.5, 103.8, 90.9, 18.8, 11.4.

HRMS (ESI, +): *m/z* calc. [M-H]$^+$: 289.1629, found: 289.1632.

^{1}H-NMR (500 MHz, Chloroform-*d*, 298 K)

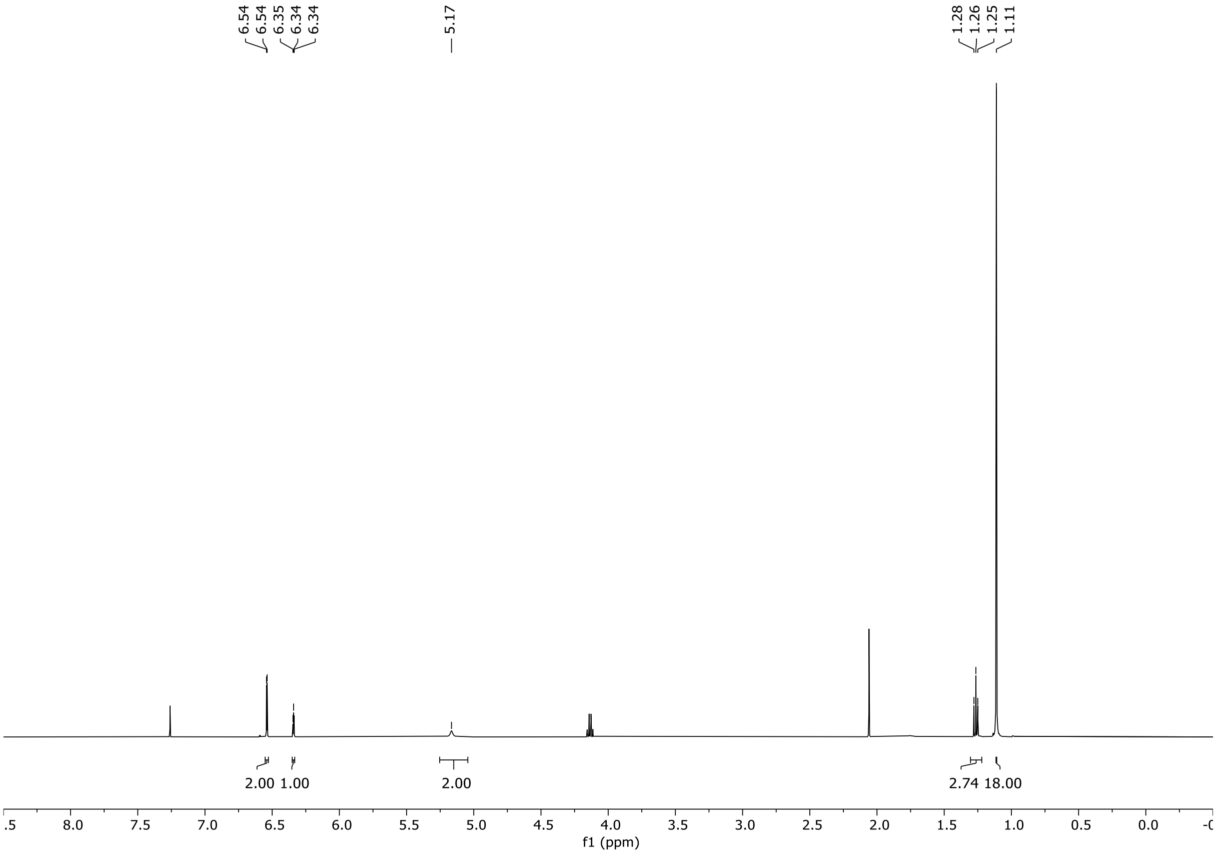

^{13}C{^{1}H}-NMR (126 MHz, Chloroform-*d*, 298 K)

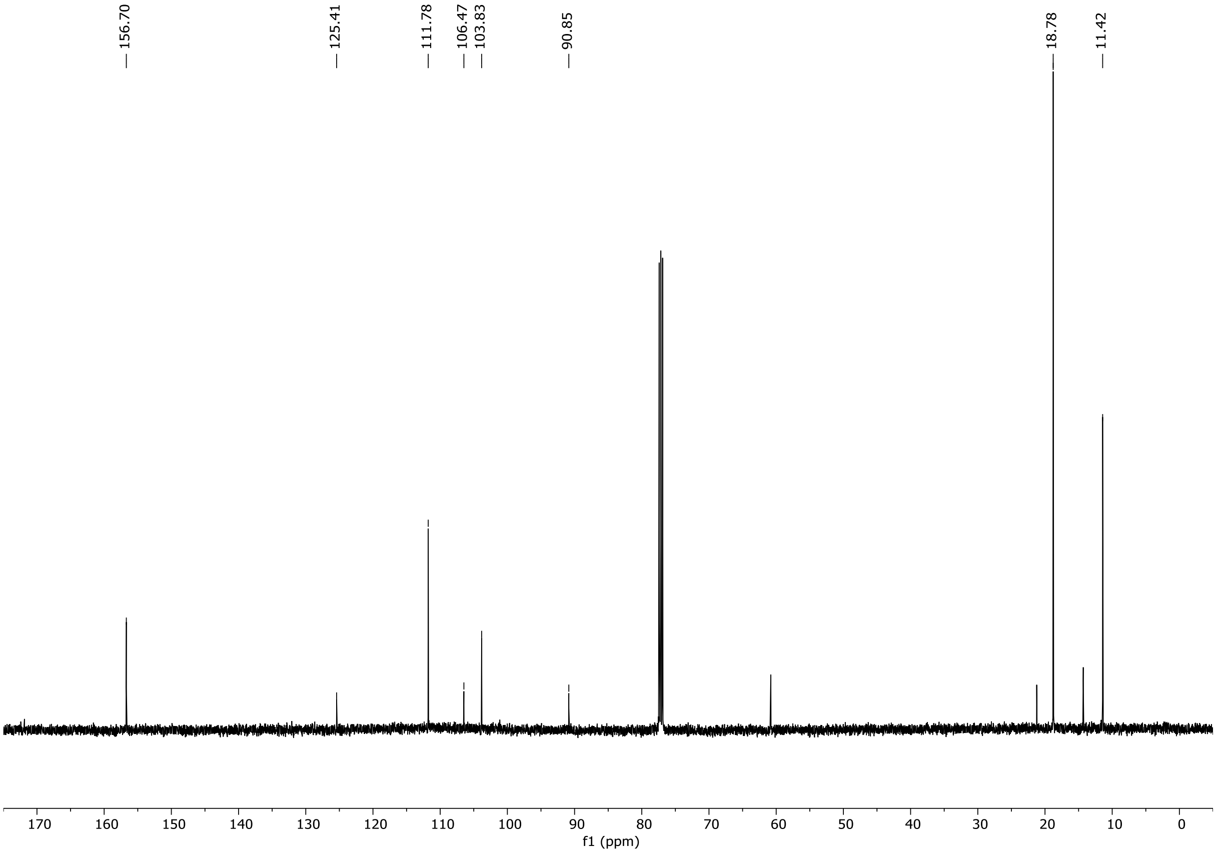

HRMS (ESI, +)

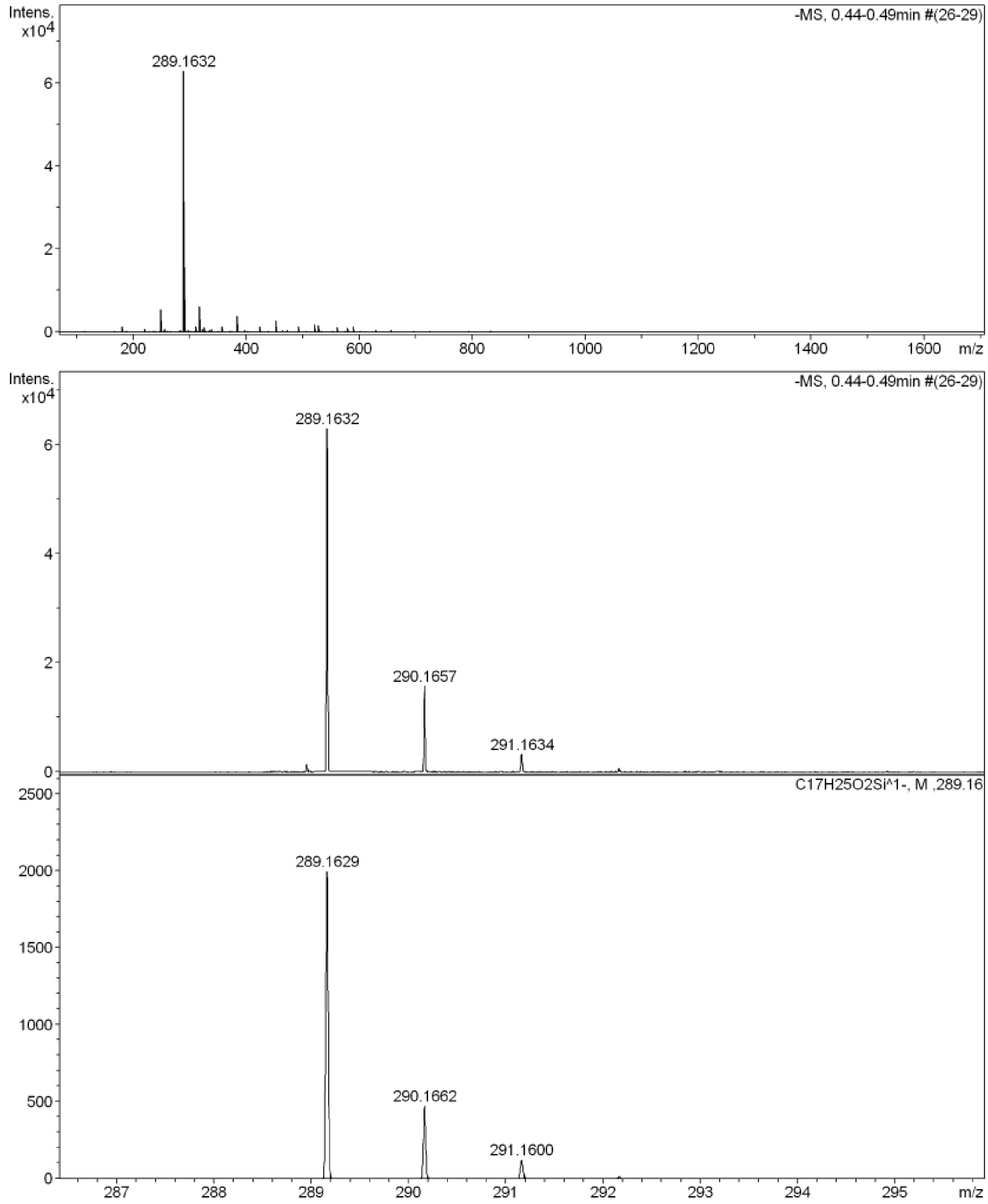

Intens.
x10^4
-MS, 0.44-0.49min #(26-29)
289.1632
6
4
2
0
200
400
600
800
1000
1200
1400
1600
m/z
Intens.
x10^4
-MS, 0.44-0.49min #(26-29)
289.1632
290.1657
291.1634
C17H25O2Si^1-, M ,289.16
2500
2000
1500
1000
500
0
289.1629
290.1662
291.1600
287
288
289
290
291
292
293
294
295
m/z

Synthesis of **6**

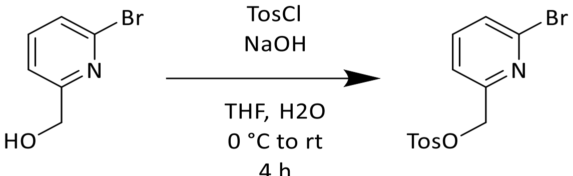

6, 93%

2-bromo-6-hydroxymethylpyridine (5.00 g, 25.6 mmol, 1.0 equiv.) was dissolved in THF (100 mL) and cooled to 0 °C. To the solution NaOH (3.19 g, 79.8 mmol, 3.0 equiv.) dissolved in water (25 mL) was added, followed by the addition of p-Toluenesulfonyl chloride (5.58 g, 29.3 mmol, 1.1 equiv.) dissolved in THF (20 mL). The reaction mixture was allowed to warm to rt and after 4 h water and Et_2O were added. The resulting mixture was washed with NH_4Cl, water and brine once each, dried over $MgSO_4$ and the solvent was removed *in vacuo* to afford **6** (8.49 g, 24.8 mmol, 93%) as a white solid. The spectroscopic data is in agreement with literature reports.[28]

^{1}H-NMR (600 MHz, Chloroform-*d*, 298 K, δ/ppm): 7.88 – 7.77 (m, 2H), 7.56 (app. t, $^{3}J_{HH}$ =7.8 Hz, 1H), 7.45 – 7.38 (m, 2H), 7.38 – 7.32 (m, 2H), 5.09 (s, 2H), 2.45 (s, 3H).

^{13}C{^{1}H}-NMR (151 MHz, Chloroform-*d*, 298 K, δ/ppm): 155.37, 145.45, 141.58, 139.40, 132.58, 130.14, 128.26, 127.87, 120.60, 70.79, 21.84.

HRMS (APCI, +): *m/z* calc. $[M+H]^{+}$: 341.9794, found: 341.9792.

^{1}H-NMR (600 MHz, Chloroform-*d*, 298 K)

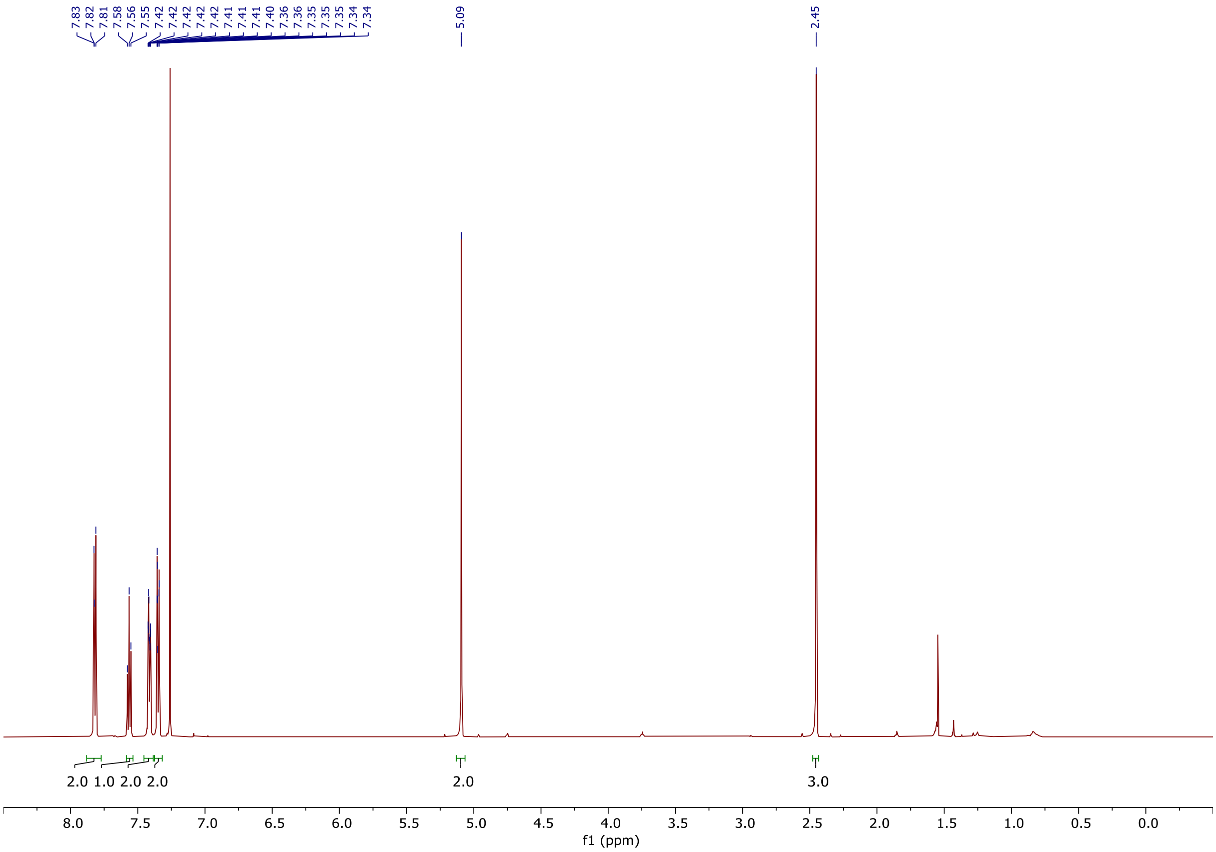

^{13}C{^{1}H}-NMR (151 MHz, Chloroform-*d*, 298 K)

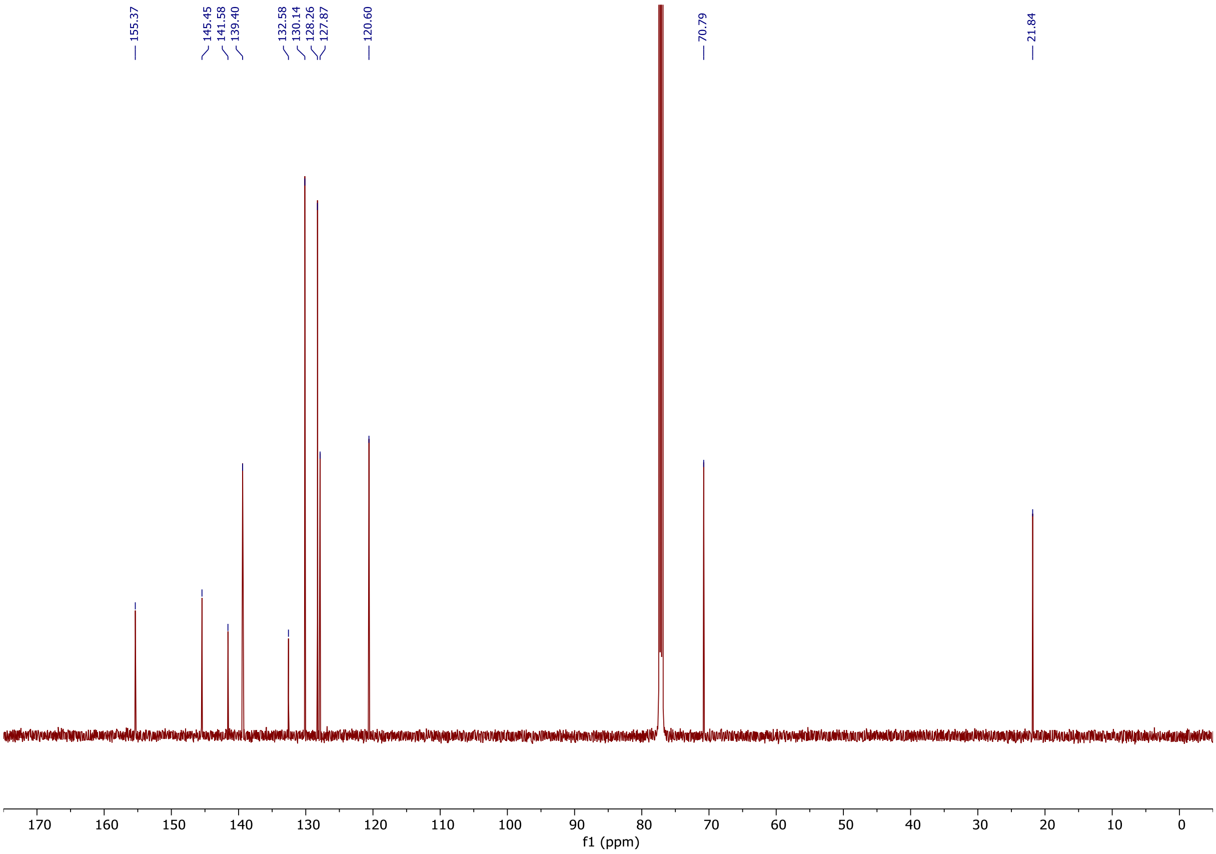

HRMS (APCI, +)

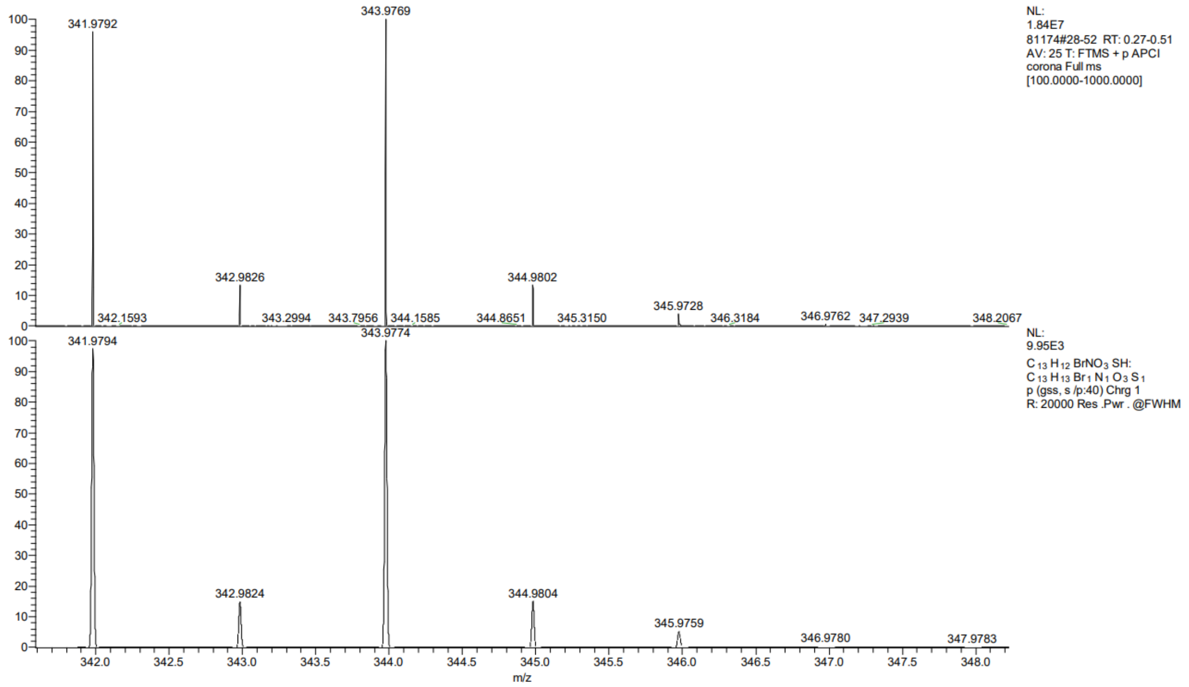
341.9792
343.9769
342.9826
344.9802
345.9728
342.1593
343.2994
343.7956
344.1585
344.8651
345.3150
346.3184
346.9762
347.2939
348.2067
NL:
1.84E7
81174#28-52 RT: 0.27-0.51
AV: 25 T: FTMS + p APCI
corona Full ms
[100.0000-1000.0000]
341.9794
343.9774
342.9824
344.9804
345.9759
346.9780
347.9783
NL:
9.95E3
C 13 H 12 BrNO3 SH:
C 13 H 13 Br 1 N 1 O 3 S 1
p (gss, s /p:40) Chrg 1
R: 20000 Res .Pwr . @FWHM
m/z

Synthesis of **7**

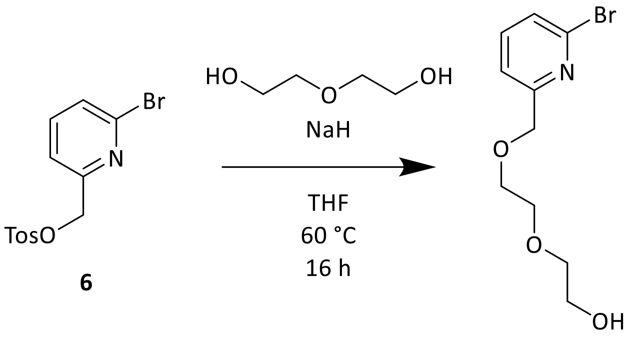

7, 85%

In a flame dried reaction flask sodium hydrate (60% in mineral oil, 777 mg, 19.4 mmol, 1.5 equiv.) was suspended in dry THF (80 mL) and diethylene glycol (8250 mg, 77.7 mmol, 7.38 mL, 6.0 equiv.) was added dropwise while stirring. After 5 min **6** (4434 mg, 12.9 mmol, 1.0 equiv.) dissolved in dry THF (50 mL) was added and the reaction mixture was heated to 60 °C for 16 h. The reaction was then cooled to 0 °C and water was added. The resulting mixture was diluted with EtOAc and washed with NH_4Cl once. The aqueous phase was extracted with EtOAc tree times and the combined organic layers were dried over $MgSO_4$ and the solvent was removed *in vacuo*. The crude product was purified by column chromatography (SiO_2, petroleum ether (40 – 60 °C):acetone (2:1 to 1:1)) to afford **7** (3050 mg, 11.0 mmol, 85%) as a colourless oil.

^{1}H-NMR (600 MHz, Chloroform-*d*, 298 K, *δ*/ppm): 7.57 (app. t, $^3J_{HH}$ =7.7 Hz, 1H), 7.48 – 7.43 (m, 1H), 7.41 – 7.36 (m, 1H), 4.67 (s, 2H), 3.77 – 3.73 (m, 6H), 3.65 – 3.62 (m, 2H).

^{13}C{^{1}H}-NMR (151 MHz, Chloroform-*d*, 298 K, *δ*/ppm): 160.26, 141.48, 139.20, 126.85, 120.13, 73.40, 72.60, 70.55, 70.48, 61.94.

HRMS (APCI, +): *m/z* calc. $[M+H]^+$: 276.0230, found: 276.0228.

^{1}H-NMR (600 MHz, Chloroform-*d*, 298 K)

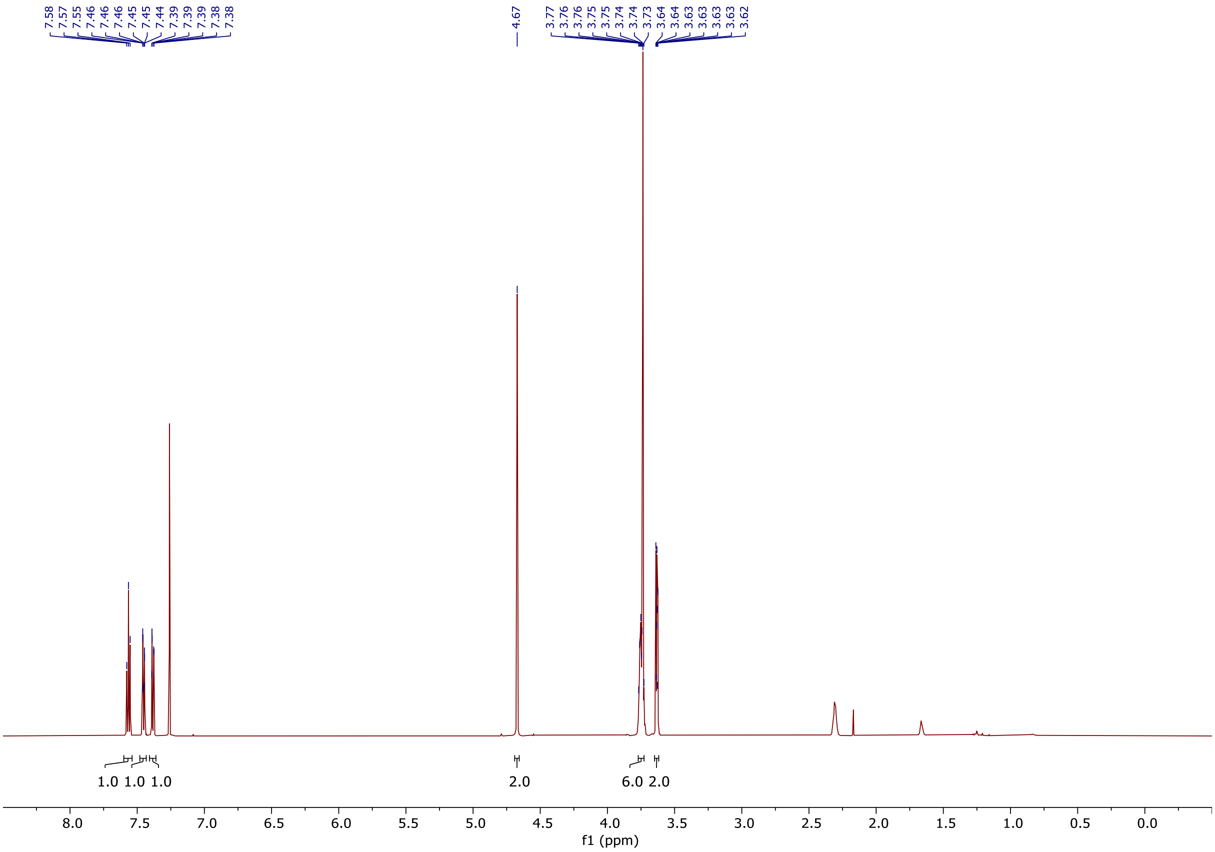

^{13}C{^{1}H}-NMR (151 MHz, Chloroform-*d*, 298 K)

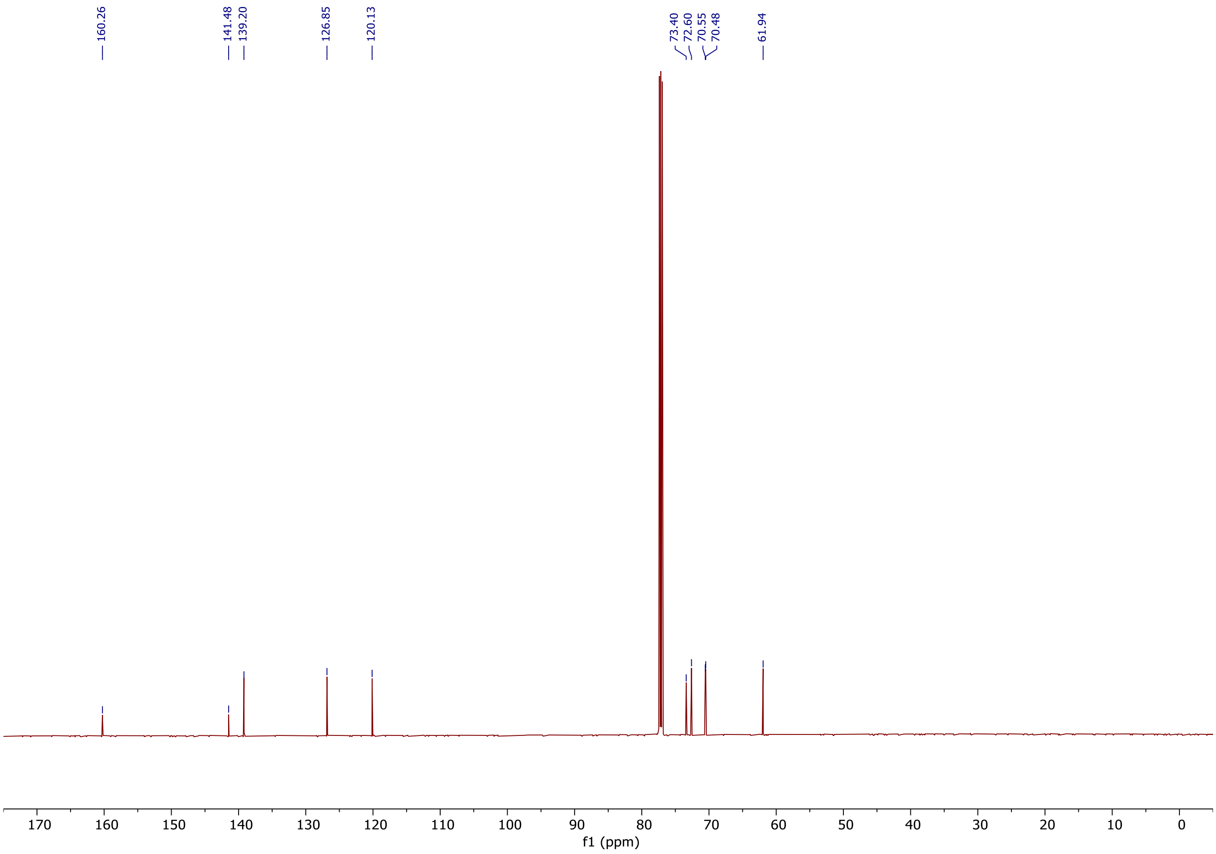

HRMS (APCI, +)

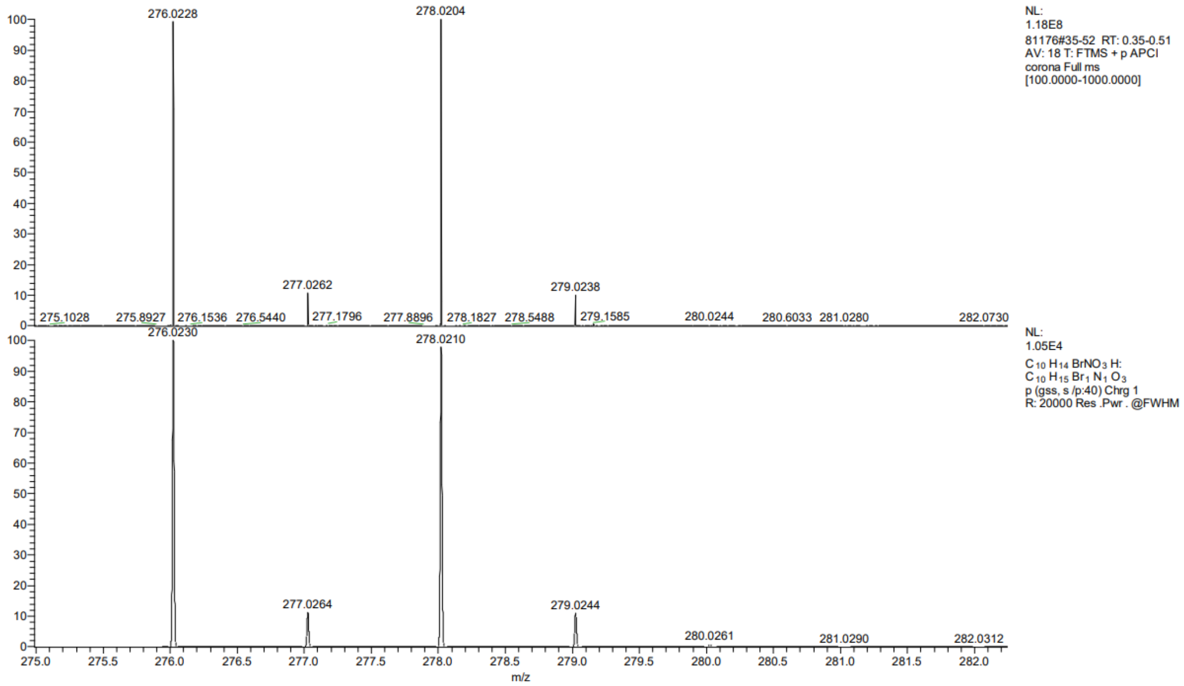
276.0228
278.0204
277.0262
279.0238
NL:
1.18E8
81176#35-52 RT: 0.35-0.51
AV: 18 T: FTMS + p APCI
corona Full ms
[100.0000-1000.0000]
276.0230
278.0210
277.0264
279.0244
NL:
1.05E4
C 10 H 14 BrNO 3 H:
C 10 H 15 Br 1 N 1 O 3
p (gss, s /p:40) Chrg 1
R: 20000 Res .Pwr . @FWHM
m/z

Synthesis of **8**

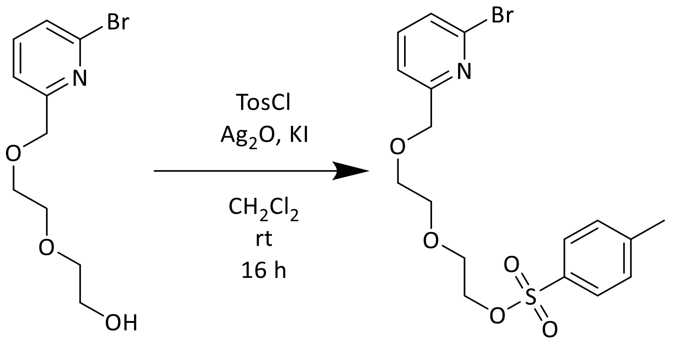

7 **8**, 100%

7 (3050 mg, 11.0 mmol, 1.0 equiv.) was dissolved in CH_2Cl_2 (100 mL) and p-Toluenesulfonyl chloride (4317 mg, 22.6 mmol, 2.05 equiv.), silver(I) oxide (5430 mg, 23.2 mmol, 2.1 equiv.) and potassium iodide (3760 mg, 22.6 mmol, 2.05 equiv.) were added. The reaction was protected from light and stirred for 16 h at rt. Afterwards the reaction mixture was filtered over silica gel (SiO_2), eluted with acetone and the solvent was removed *in vacuo*. **8** (4750 mg, 11.0 mmol, 100%) was isolated as a colorless oil after purification by column chromatography (SiO_2, , petroleum ether (40 – 60 °C):acetone (2:1)).

^{1}H-NMR (500 MHz, Chloroform-*d*, 298 K, δ/ppm): 7.81 – 7.77 (m, 2H), 7.58 (app. t, $^3J_{HH}$ = 7.7 Hz, 1H), 7.46 (dd, $^3J_{HH}$ = 7.7, $^4J_{HH}$ = 0.8 Hz, 1H), 7.38 (dd, $^3J_{HH}$ = 7.7, $^4J_{HH}$ = 0.8 Hz, 1H), 7.35 – 7.31 (m, 2H), 4.62 (s, 2H), 4.19 – 4.16 (m, 2H), 3.73 – 3.70 (m, 2H), 3.66 (hept, $^3J_{HH}$ = 3.7 Hz, 4H), 2.44 (s, 3H).

^{13}C{^{1}H}-NMR (151 MHz, Chloroform-*d*, 298 K, δ/ppm): 160.30, 145.03, 141.37, 139.33, 132.88, 129.98, 128.10, 126.76, 120.10, 73.33, 70.77, 70.38, 69.38, 68.87, 21.82.

HRMS (APCI, +): *m/z* calc. $[M+H]^+$: 430.0318, found: 430.0320.

^{1}H-NMR (600 MHz, Chloroform-*d*, 298 K)

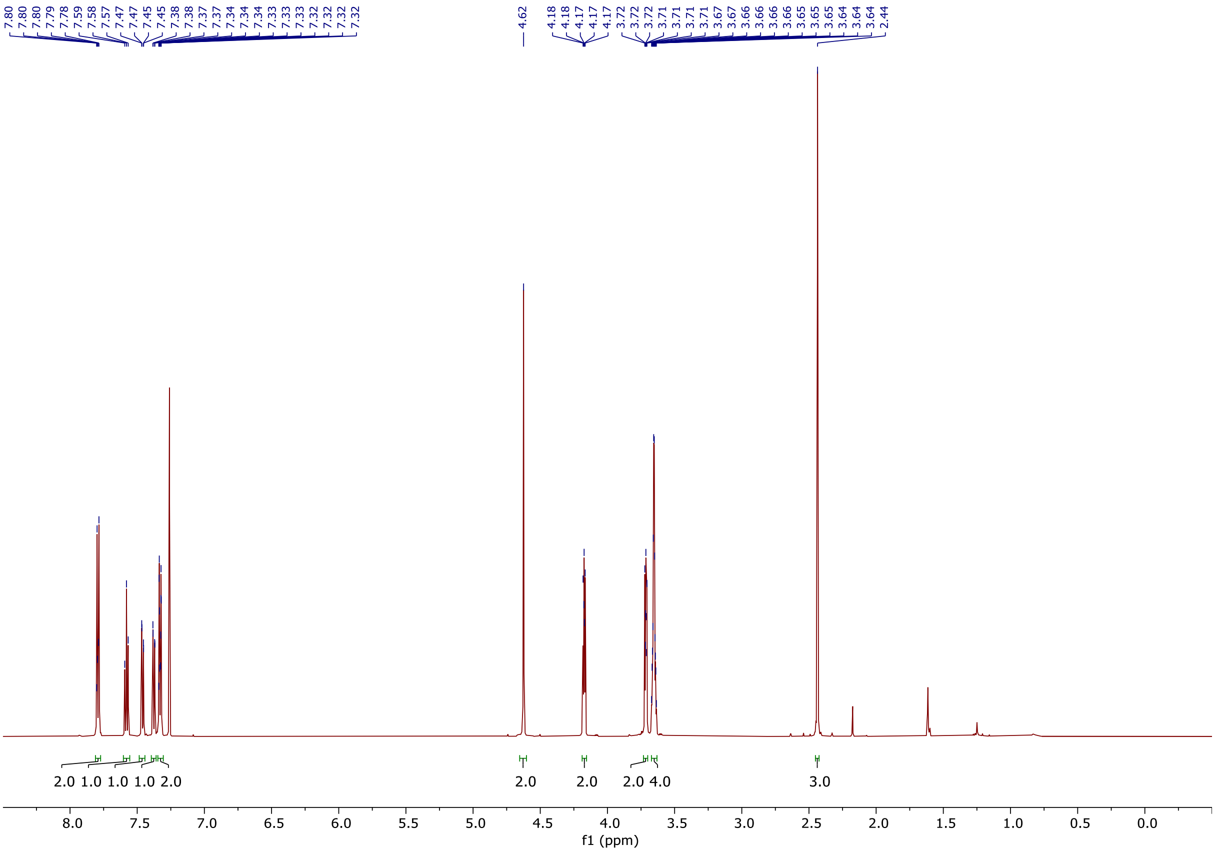

^{13}C{^{1}H}-NMR (151 MHz, Chloroform-*d*, 298 K)

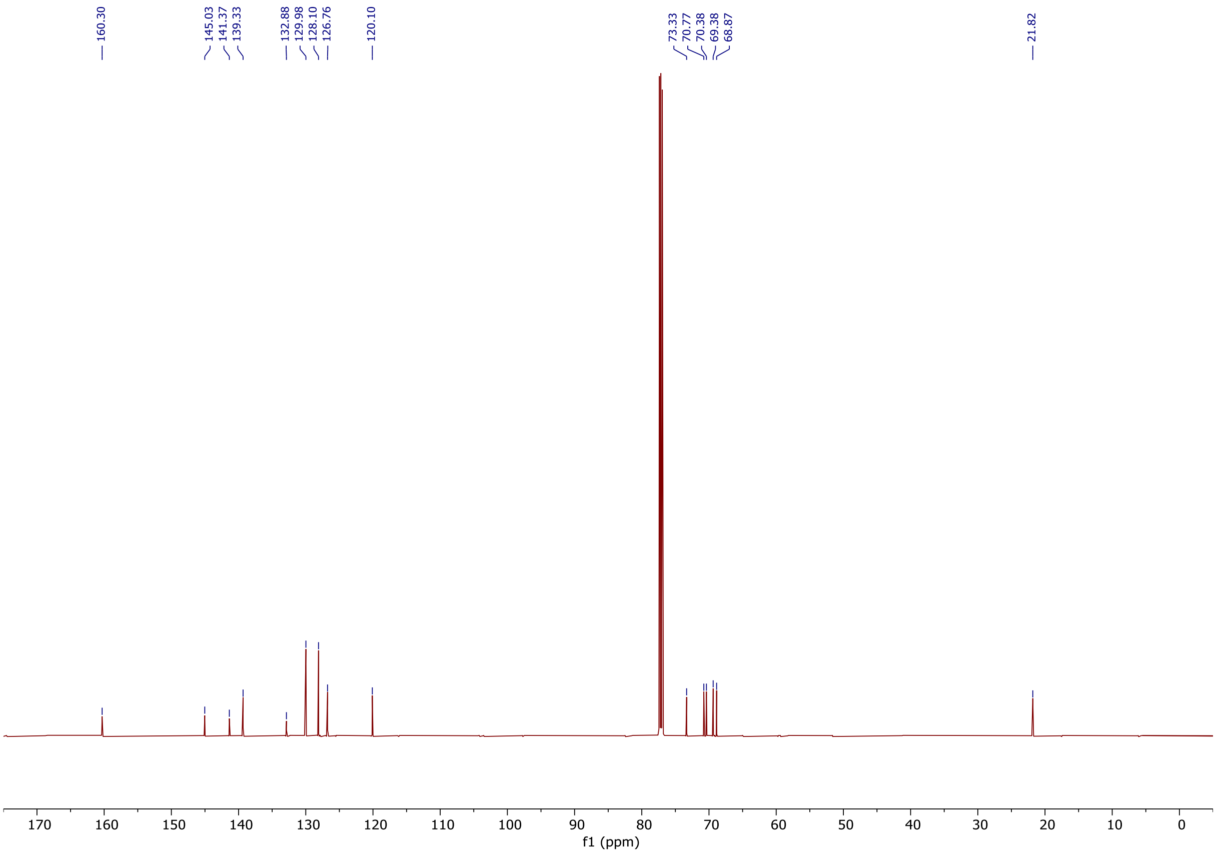

HRMS (APCI, +)

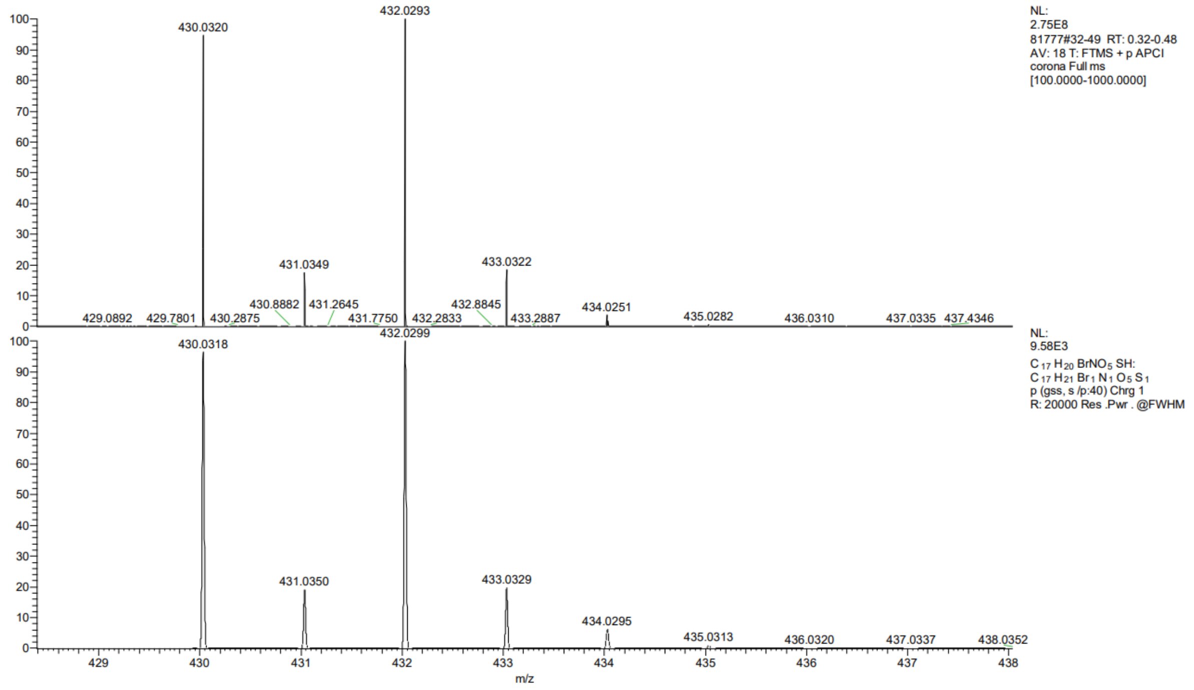

NL:
2.75E8
81777#32-49 RT: 0.32-0.48
AV: 18 T: FTMS + p APCI
corona Full ms
[100.0000-1000.0000]
430.0320
432.0293
431.0349
433.0322
434.0251
NL:
9.58E3
C 17 H 20 BrNO 5 SH:
C 17 H 21 Br 1 N 1 O 5 S 1
p (gss, s /p:40) Chrg 1
R: 20000 Res .Pwr . @FWHM
430.0318
432.0299
431.0350
433.0329
434.0295
m/z

Synthesis of **9**:

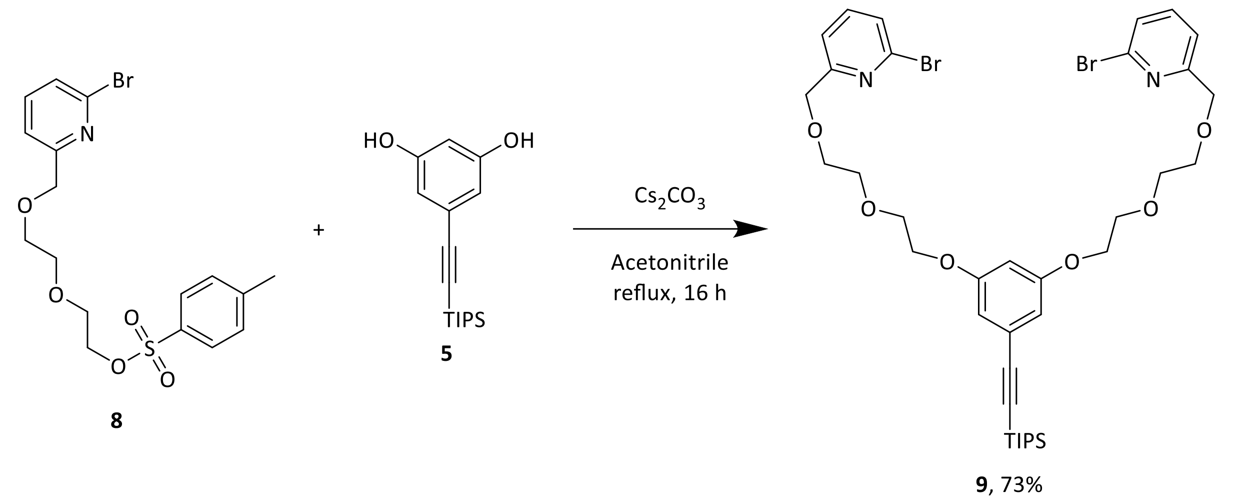

8 (2241 mg, 5.21 mmol, 2.5 equiv), **5** (605 mg, 2.08 mmol, 1.0 equiv) and cesium carbonate (4072 mg, 12.5 mmol, 6.0 equiv.) were placed into a round bottom flask and suspended in acetonitrile (60 mL). The reaction flask was equipped with a reflux condenser and the reaction mixture was refluxed for 16 h. Afterwards water and EtOAc were added. The two phases were separated and the aqueous phase was extracted tree times with EtOAc. The combined organic layers were dried over $MgSO_4$ and the solvent was removed *in vacuo*. **9** (1239 mg, 1.536 mmol, 73%) was isolated as a colorless oil after purification by column chromatography (SiO_2, petroleum ether (40 – 60 °C):acetone (3:1 to 2:1)).

^{1}H-NMR (600 MHz, Chloroform-*d*, 298 K, δ/ppm): 7.52 (app. t, $^3J_{HH}$ = 7.7 Hz, 2H), 7.47 – 7.44 (m, 2H), 7.37 – 7.33 (m, 2H), 6.66 – 6.62 (m, 2H), 6.48 (t, $^4J_{HH}$ = 1.2 Hz, 1H), 4.66 (s, 4H), 4.10 (dt, $^3J_{HH}$ = 4.5, $^4J_{HH}$ = 2.1 Hz, 4H), 3.85 (dt, $^3J_{HH}$ = 4.9, $^4J_{HH}$ = 2.3 Hz, 4H), 3.78 – 3.73 (m, 8H), 1.11 (s, 21H).

^{13}C{^{1}H}-NMR (151 MHz, Chloroform-*d*, 298 K, δ/ppm): 160.50, 159.67, 141.38, 139.18, 126.70, 124.91, 120.10, 110.90, 106.99, 102.99, 90.41, 73.40, 70.85, 70.57, 69.83, 67.71, 18.81, 11.42.

HRMS (APCI, +): *m/z* calc. $[M+H]^+$: 805.1878, found: 805.1874.

^{1}H-NMR (600 MHz, Chloroform-*d*, 298 K)

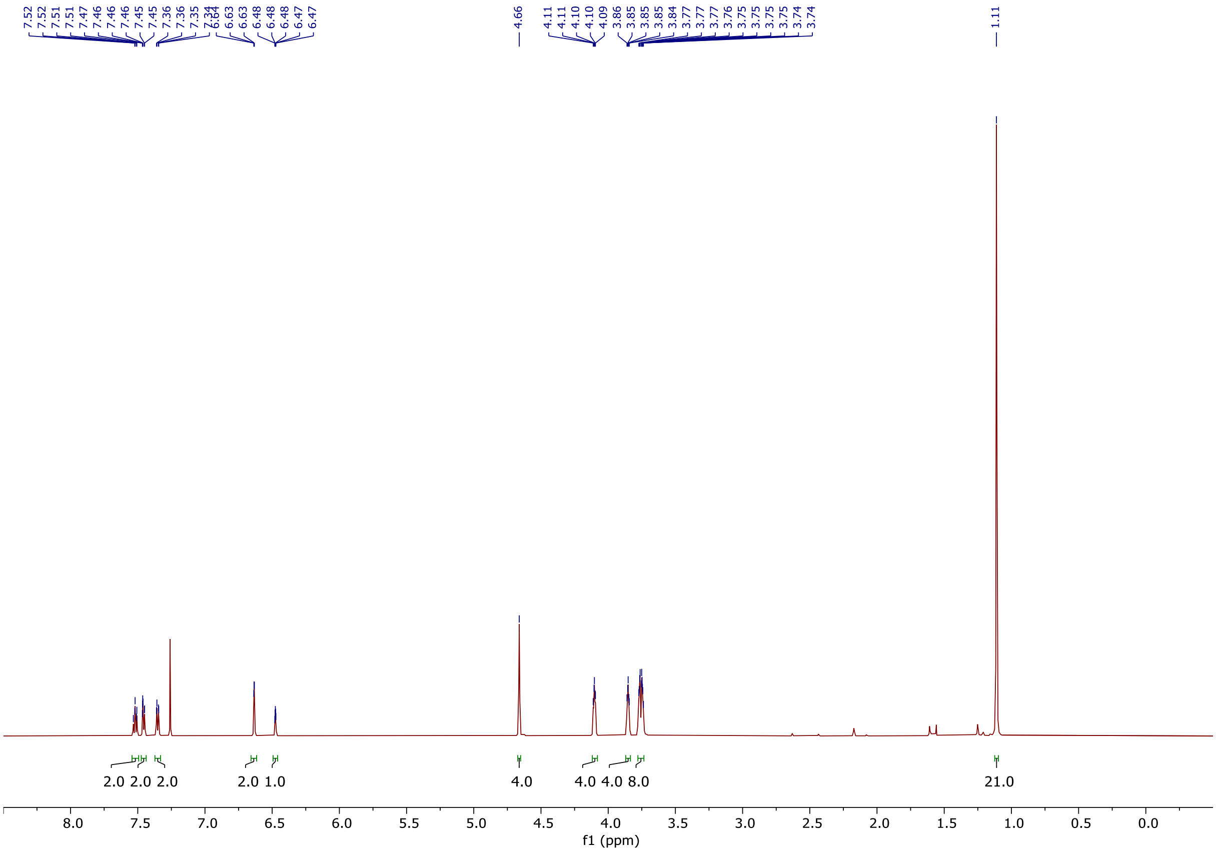

^{13}C{^{1}H}-NMR (151 MHz, Chloroform-*d*, 298 K)

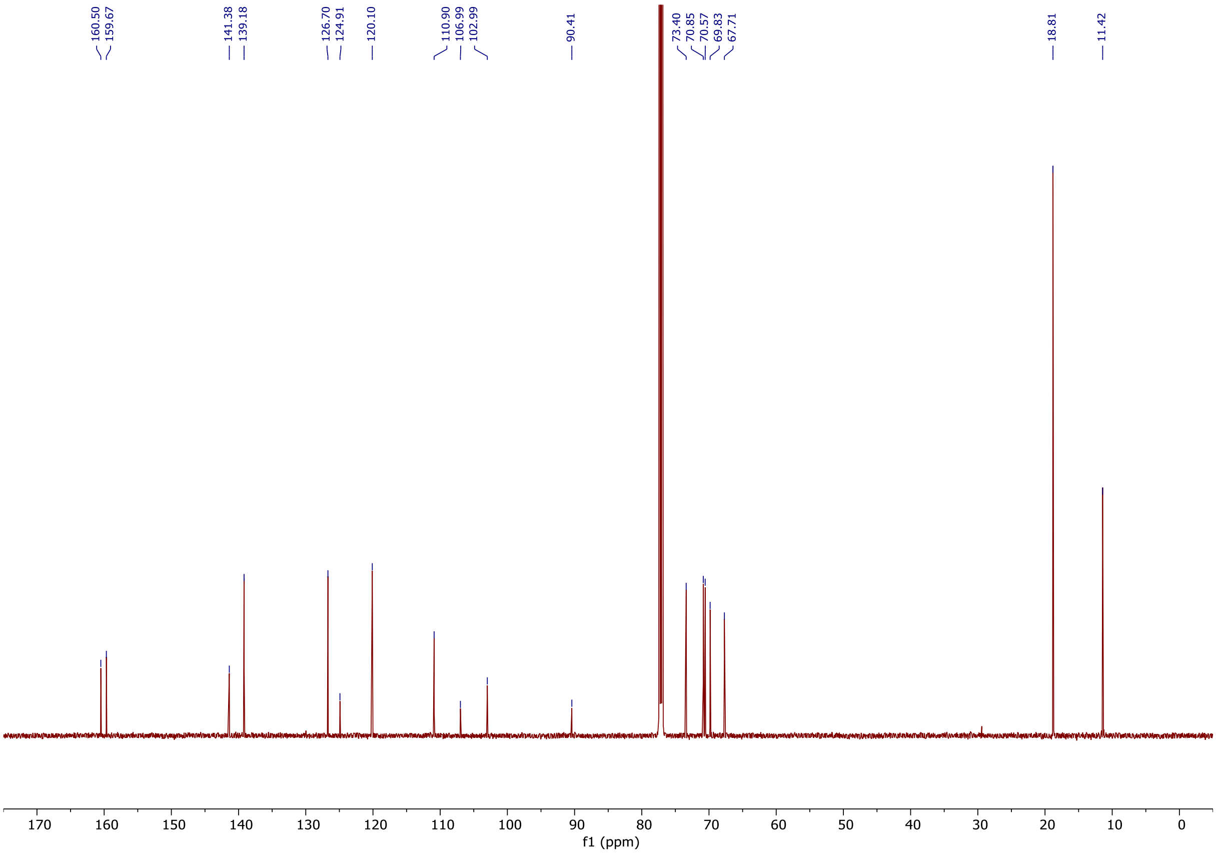

HRMS (APCI, +)

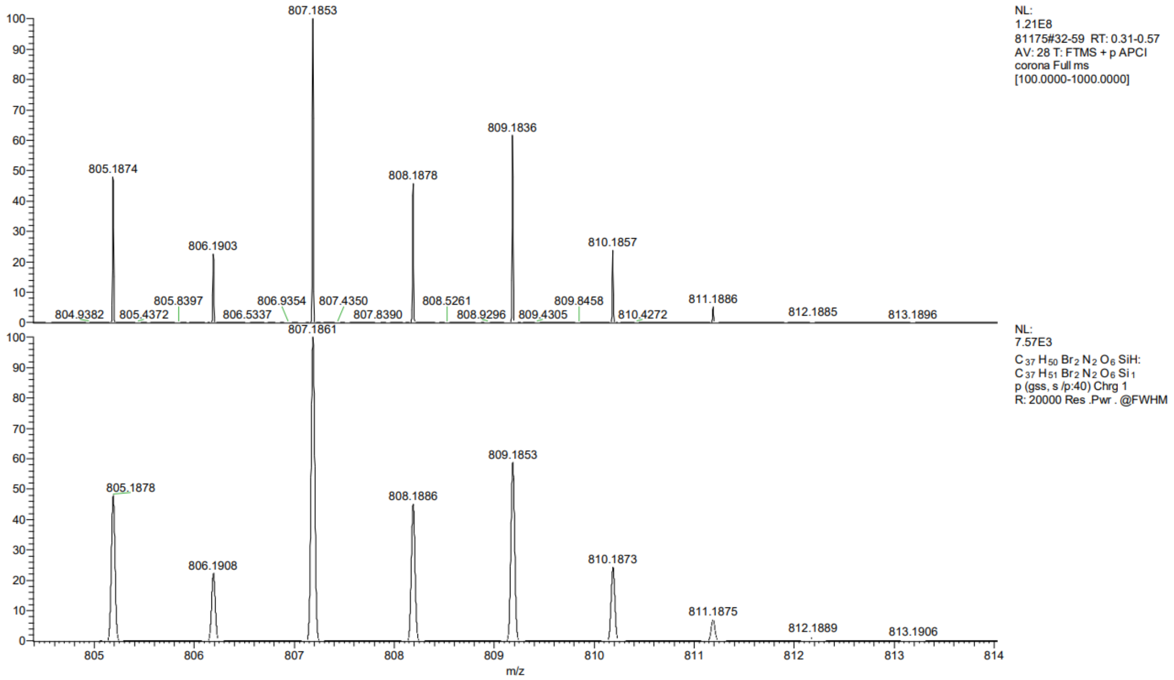
NL:
1.21E8
81175#32-59 RT: 0.31-0.57
AV: 28 T: FTMS + p APCI
corona Full ms
[100.0000-1000.0000]
NL:
7.57E3
C 37 H50 Br2 N2 O6 SiH:
C 37 H51 Br2 N2 O6 Si 1
p (gss, s /p:40) Chrg 1
R: 20000 Res .Pwr . @FWHM
807.1853
809.1836
805.1874
808.1878
806.1903
810.1857
811.1886
812.1885
813.1896
807.1861
809.1853
805.1878
808.1886
806.1908
810.1873
811.1875
812.1889
813.1906
m/z

Synthesis of **10**

The procedure was adapted from literature.[27]

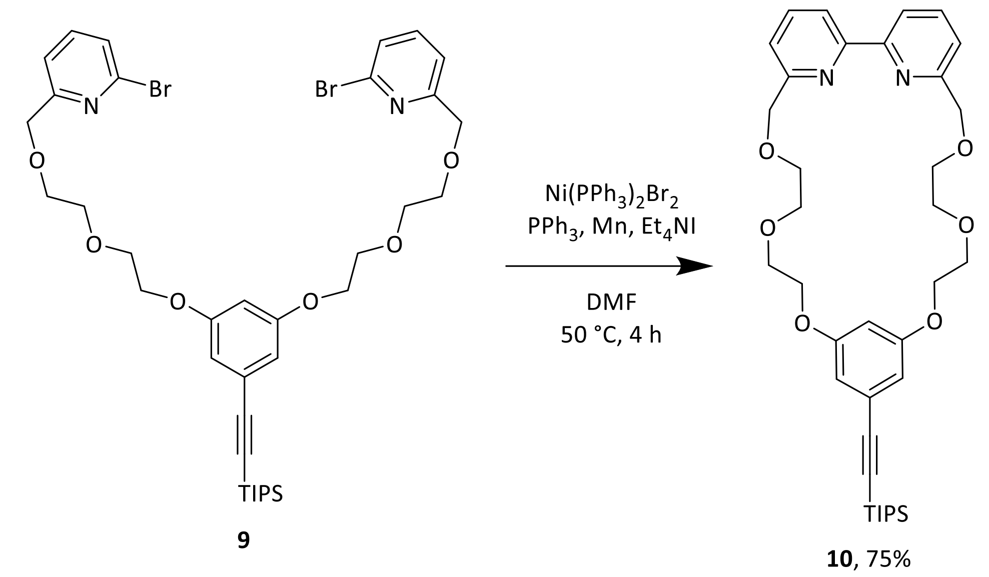

To a flame dried reaction flask $Ni(PPh_3)_2Br_2$ (2710 mg, 3.64 mmol, 5.0 equiv.), PPh_3 (1930 mg, 7.29 mmol, 10 equiv.), Mn (2000 mg, 36.4 mmol, 50 equiv.) and NEt_4I (937 mg, 3.64 mmol, 5.0 equiv.) were added and suspended in dry DMF (25 mL). The suspension was degassed by purging with argon and sonicated for 10 min, followed by stirring at 50 °C for 1 h. **9** (588 mg, 729 µmol, 1.0 equiv.) was dissolved in dry DMF (20 mL) and degassed by purging with argon for 15 min. The solution of **9** was then added dropwise via syringe over 4 h. After the addition the suspension was stirred for one hour at 50 °C. To the cooled reaction mixture CH_2Cl_2 and EDTA-NH_3 solution were added and the combined phases were filtered through a pad of Celite. The two phases were separated and the combined organic layers were then washed once with LiCl solution (5% aqueous solution), tree times with water and once with brine, dried over $MgSO_4$ and the solvent was removed *in vacuo*. **10** (355 mg, 549 µmol, 75%) was isolated as a colorless oil after purification by column chromatography (SiO_2, petroleum ether (40 – 60 °C):acetone (3:1 to 2:1)).

^{1}H-NMR (600 MHz, Chloroform-*d*, 298 K, δ/ppm): 8.28 (d, $^3J_{HH}$ = 7.7 Hz, 2H), 7.65 (app. t, $^3J_{HH}$ = 7.7 Hz, 2H), 7.40 (dd, $^3J_{HH}$ = 7.7, $^4J_{HH}$ = 1.0 Hz, 2H), 6.56 (d, $^4J_{HH}$ = 2.3 Hz, 2H), 6.02 (t, $^4J_{HH}$ = 2.3 Hz, 1H), 4.78 (s, 4H), 3.86 (dd, $^3J_{HH}$ = 5.8, $^4J_{HH}$ = 3.8 Hz, 4H), 3.84 – 3.82 (m, 4H), 3.75 – 3.72 (m, 8H), 1.12 (s, 21H)

^{13}C{^{1}H}-NMR (151 MHz, Chloroform-*d*, 298 K, δ/ppm): 159.59, 158.16, 155.69, 137.44, 124.62, 121.97, 119.94, 110.54, 107.18, 103.12, 90.12, 74.22, 71.14, 70.39, 69.53, 67.49, 18.81, 11.43

HRMS (APCI, +): *m/z* calc. $[M+H]^+$: 647.3511, found: 647.3509.

^{1}H-NMR (600 MHz, Chloroform-*d*, 298 K)

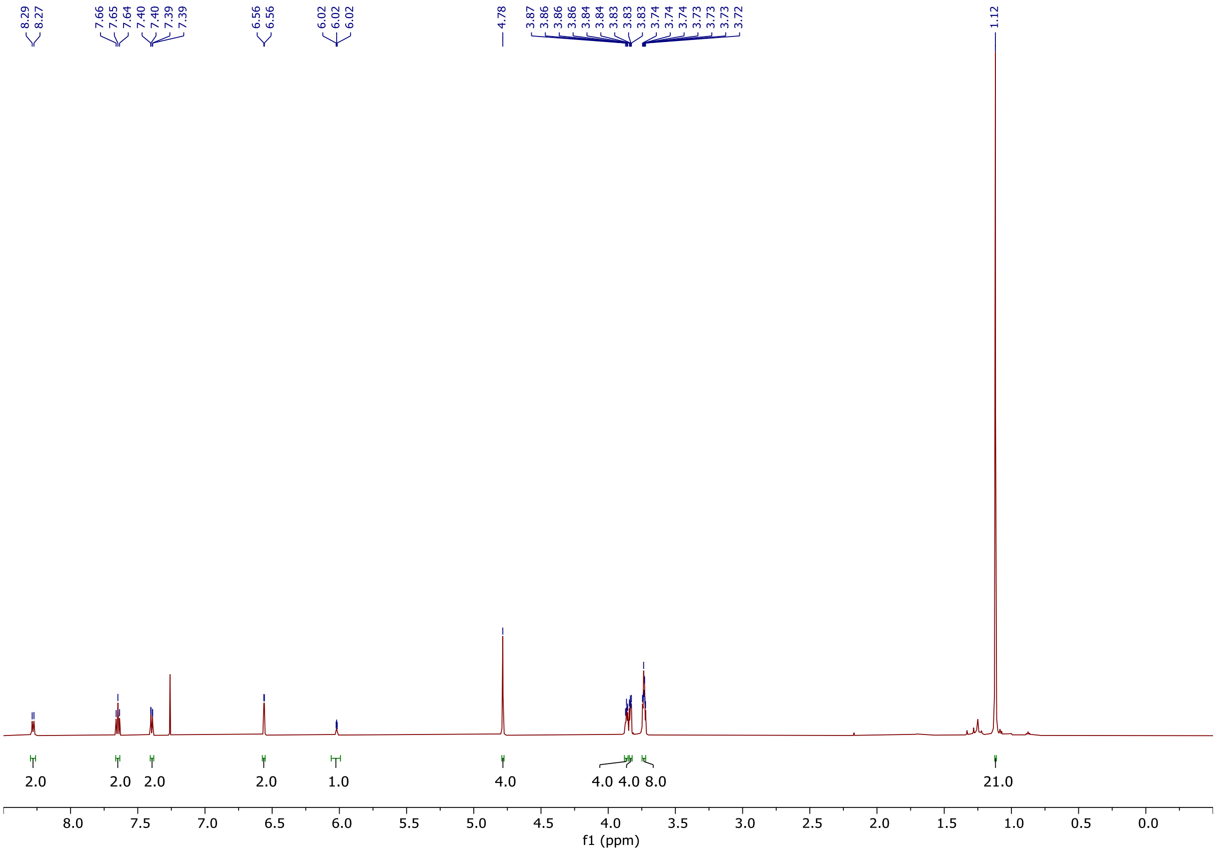

^{13}C{^{1}H}-NMR (151 MHz, Chloroform-*d*, 298 K)

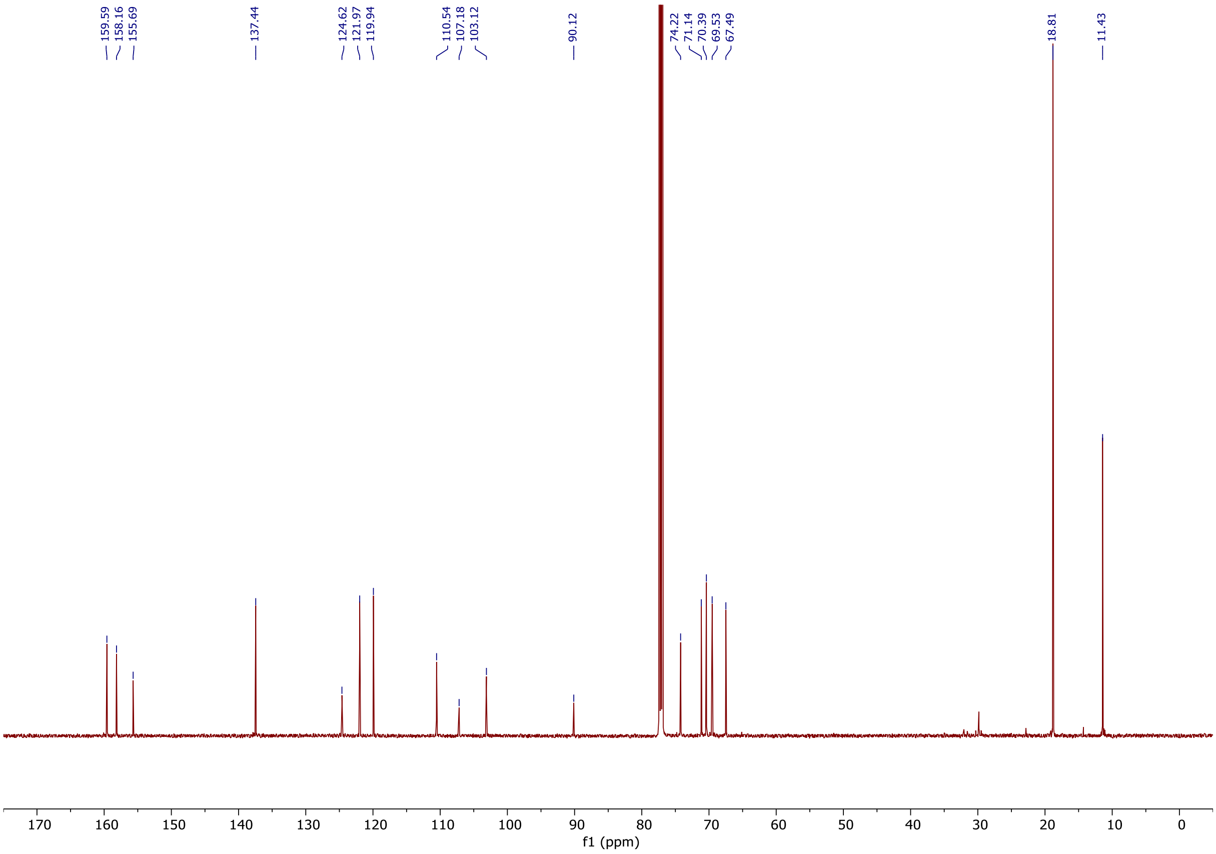

HRMS (APCI, +)

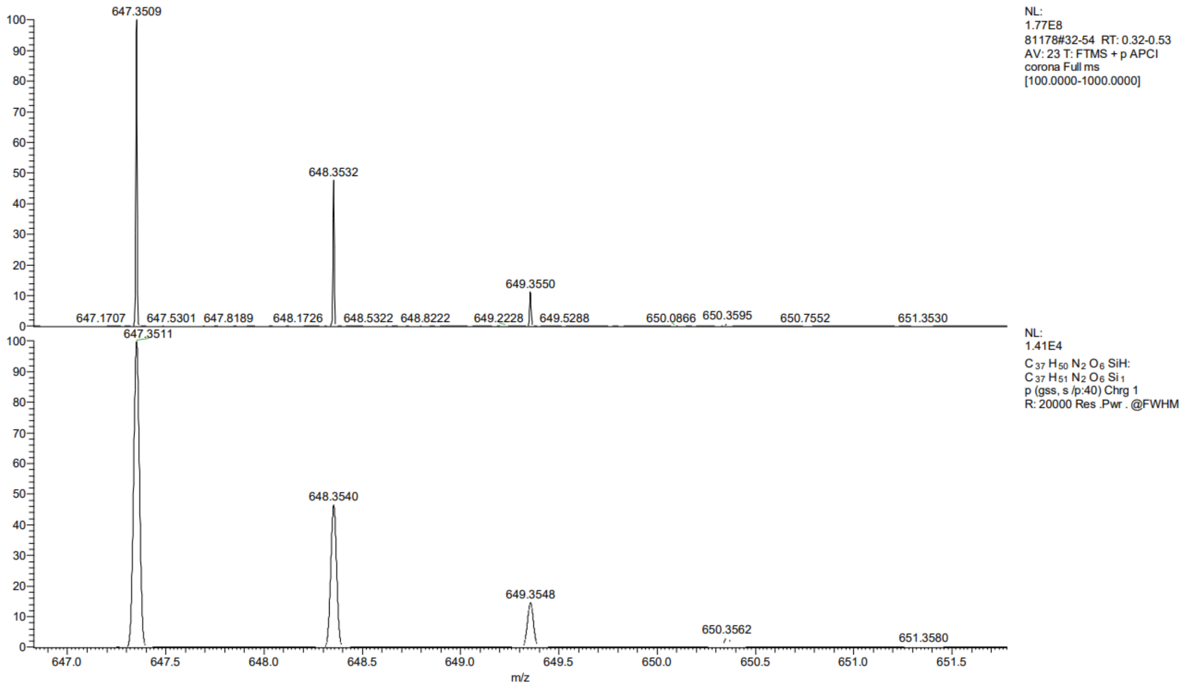

647.3509
648.3532
649.3550
647.1707
647.5301
647.8189
648.1726
648.5322
648.8222
649.2228
649.5288
650.0866
650.3595
650.7552
651.3530
NL:
1.77E8
81178#32-54 RT: 0.32-0.53
AV: 23 T: FTMS + p APCI
corona Full ms
[100.0000-1000.0000]
647.3511
648.3540
649.3548
650.3562
651.3580
NL:
1.41E4
C 37 H 50 N 2 O 6 SiH:
C 37 H 51 N 2 O 6 Si 1
p (gss, s /p:40) Chrg 1
R: 20000 Res .Pwr . @FWHM
m/z

Synthesis of **11**

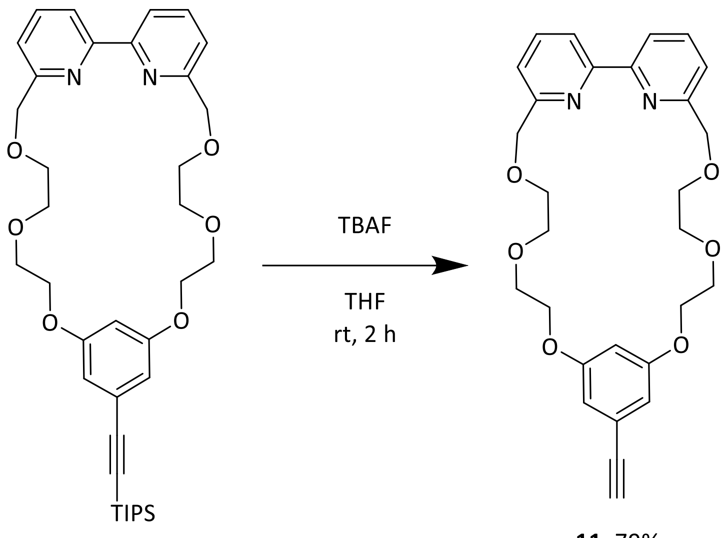

10

11, 79%

10 (110 mg, 170 µmol, 1.0 equiv.) was dissolved in THF (4 mL) and the solution was degassed by purging with argon for 10 min. Then TBAF (1 M in THF, 599 mg, 680 µmol, 4.0 equiv.) was added and the reaction mixture was degassed by purging with argon for 5 more minutes. The reaction mixture was then stirred for 2 h at rt. Afterwards NH_4Cl (sat.) was added and the mixture was diluted with EtOAc. The organic layer was washed with NH_4Cl, water and brine once each, dried over $MgSO_4$ and the solvent was removed *in vacuo* to obtain **11** (66 mg, 135 µmol, 79%) as a colorless solid.

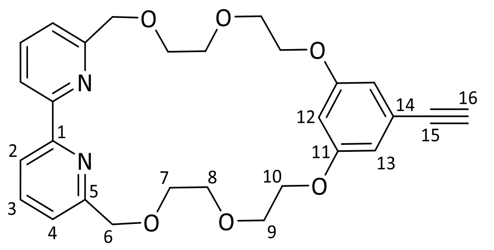

^{1}H-NMR (600 MHz, Chloroform-*d*, 298 K, *δ*/ppm): 8.28 (d, $^3J_{HH}$ = 7.7 Hz, 2H, H2), 7.64 (app. t, $^3J_{HH}$ = 7.7 Hz, 2H, H3), 7.39 (dd, $^3J_{HH}$ = 7.7, $^4J_{HH}$ = 1.1 Hz, 2H, H4), 6.57 (d, $^4J_{HH}$ = 2.3 Hz, 2H, H13), 6.05 (t, $^4J_{HH}$ = 2.4 Hz, 1H, H12), 4.78 (s, 4H, H6), 3.87 – 3.85 (m, 4H, H10), 3.84 – 3.82 (m, 4H, H7), 3.75 – 3.72 (m, 8H, H8, H9), 3.02 (s, 1H, H16).

^{13}C{^{1}H}-NMR (151 MHz, Chloroform-*d*, 298 K, *δ*/ppm): 159.68 (C11), 158.15 (C5), 155.68 (C1), 137.41 (C3), 123.14 (C14), 121.96 (C4), 119.91 (C2), 110.55 (C13), 103.62 (C12), 83.78 (C15), 76.75 (C16), 74.22 (C6), 71.15 (C8), 70.36 (C7), 69.50 (C9), 67.52 (C10).

HRMS (APCI, -): *m/z* calc. $[M+H]^+$: 491.2182, found: 491.2163.

UV/VIS (CH_2Cl_2, 20 °C) λ_{max} [nm] (ε [$L\cdot cm^{-1}\cdot mol^{-1}$]) : 291 ($14.9\cdot 10^3$).

Emission (CH_2Cl_2, 20 °C) λ_{max} [nm] (excitation [nm]) : 330 (300).

Quantum Yield ϕ_f (CH_2Cl_2, 20 °C) (excitation [nm]) : <1% , too low to be measured (300).

^{1}H-NMR (600 MHz, Chloroform-*d*, 298 K)

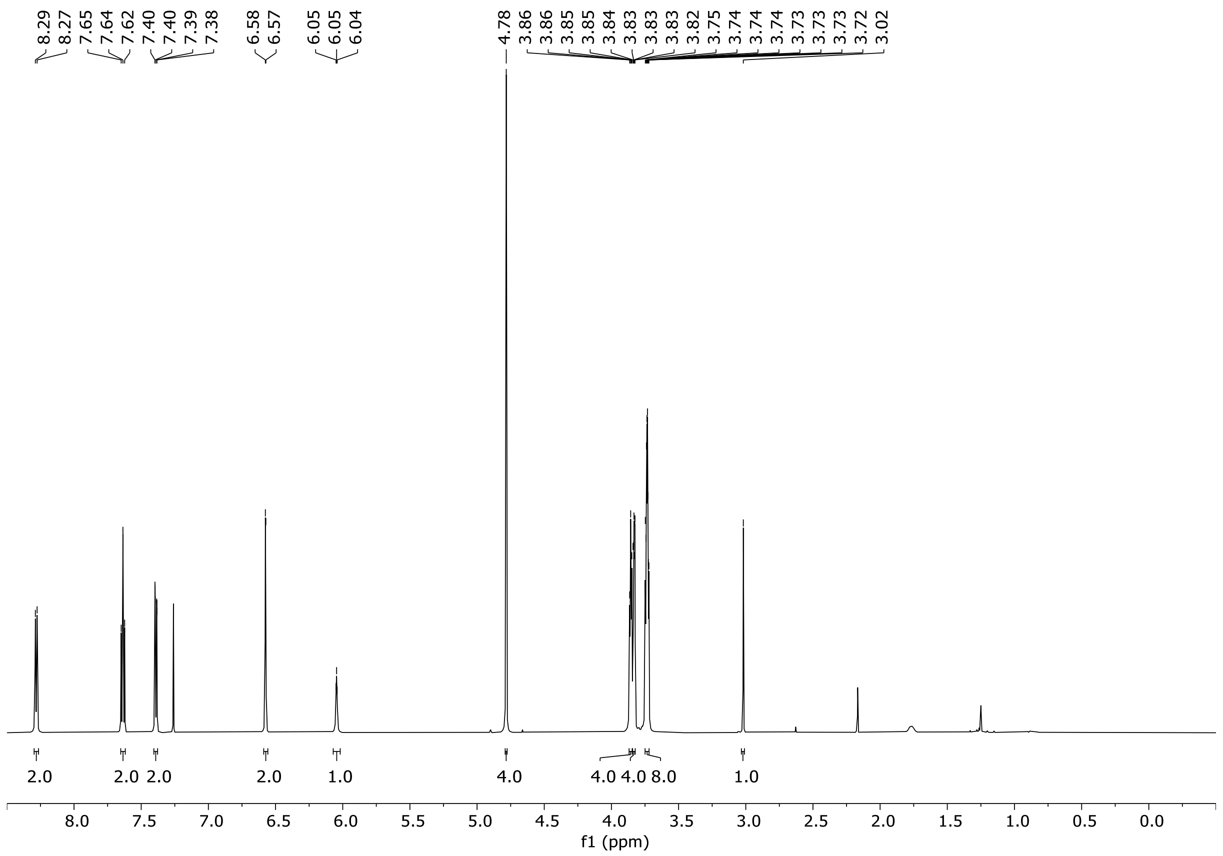

^{13}C{^{1}H}-NMR (151 MHz, Chloroform-*d*, 298 K)

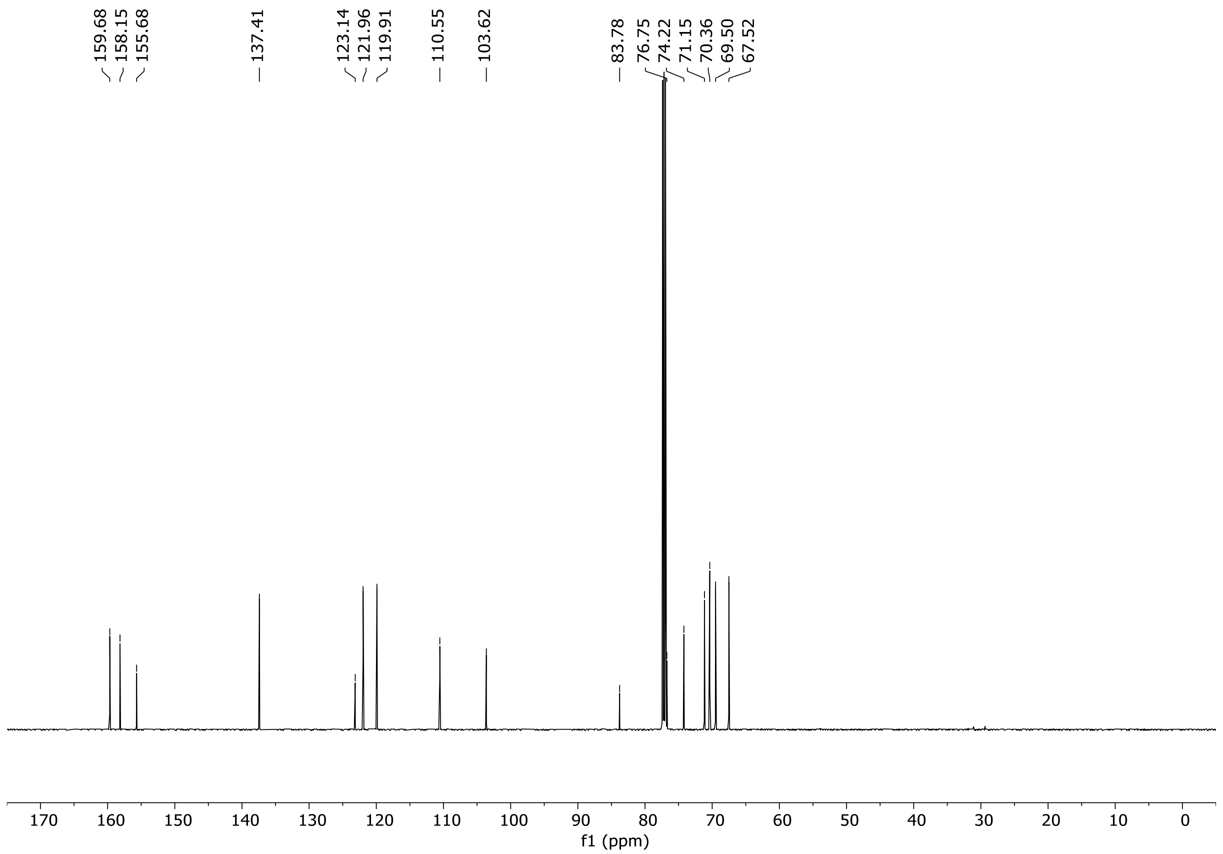

DEPTQ (151 MHz, Chloroform-*d*, 298 K)

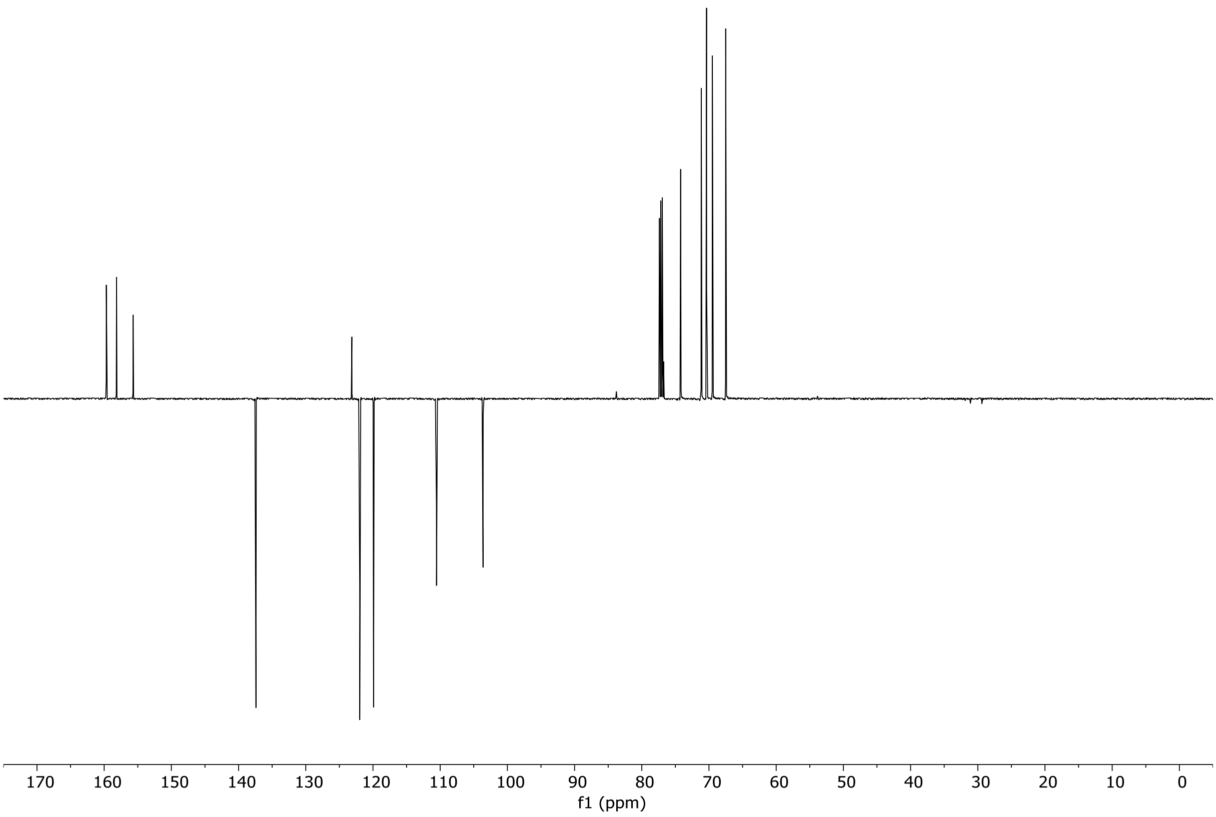

HSQC (600 Hz, Chloroform-*d*, 298 K)

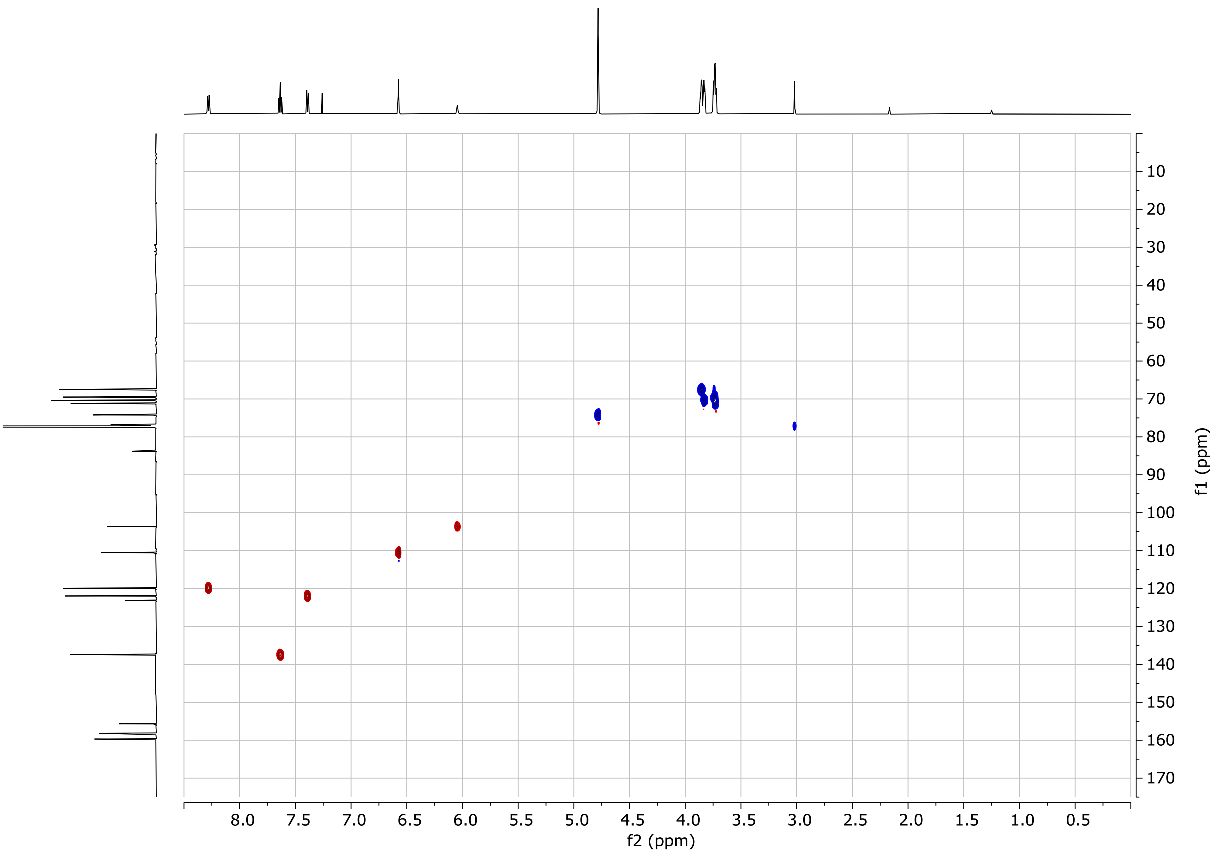

HMBC (600 Hz, Chloroform-*d*, 298 K)

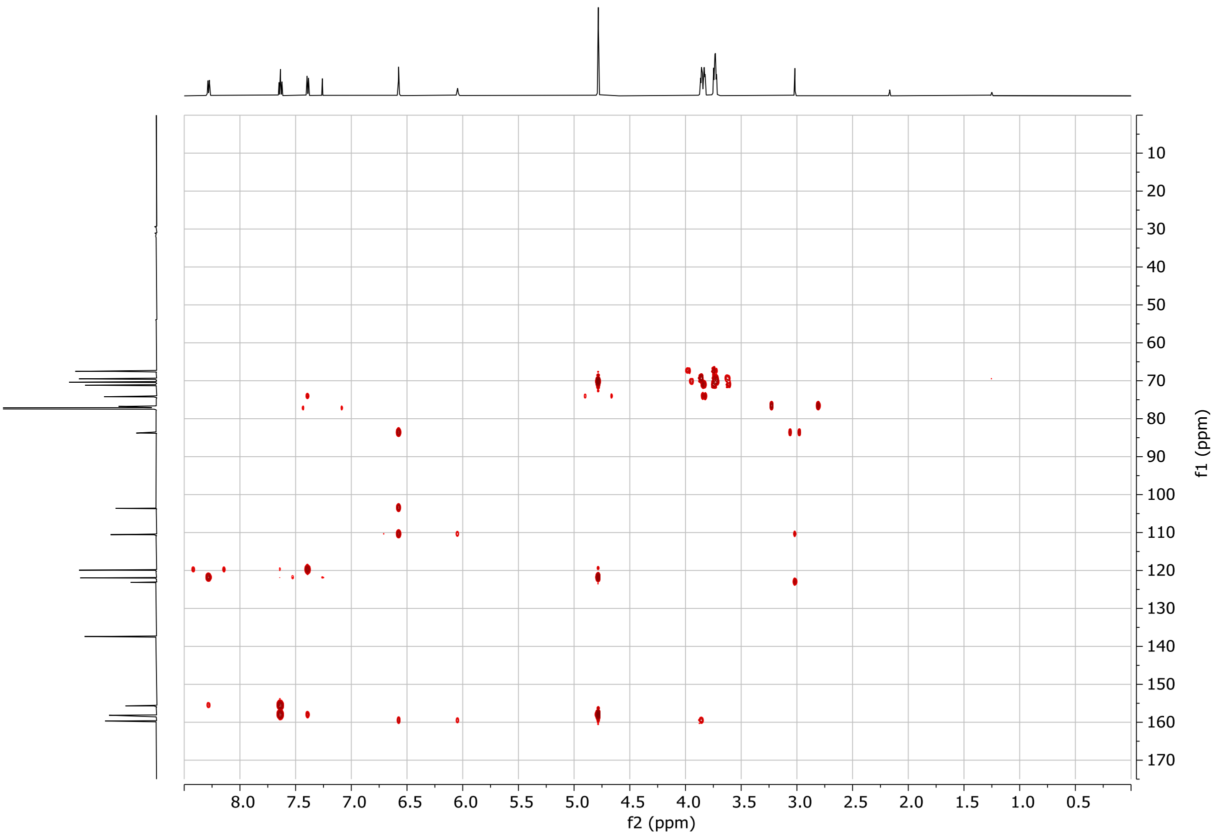

COSY (600 Hz, Chloroform-*d*, 298 K)

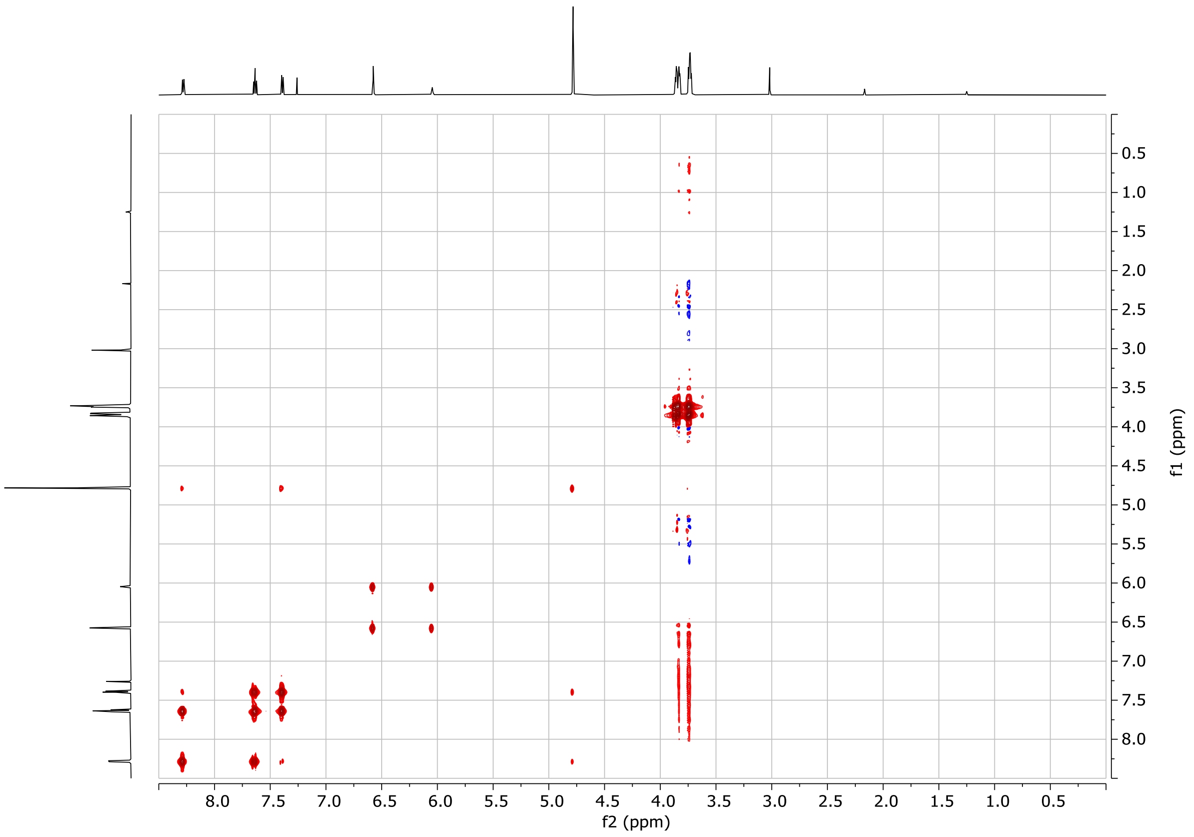

HRMS (APCI, +)

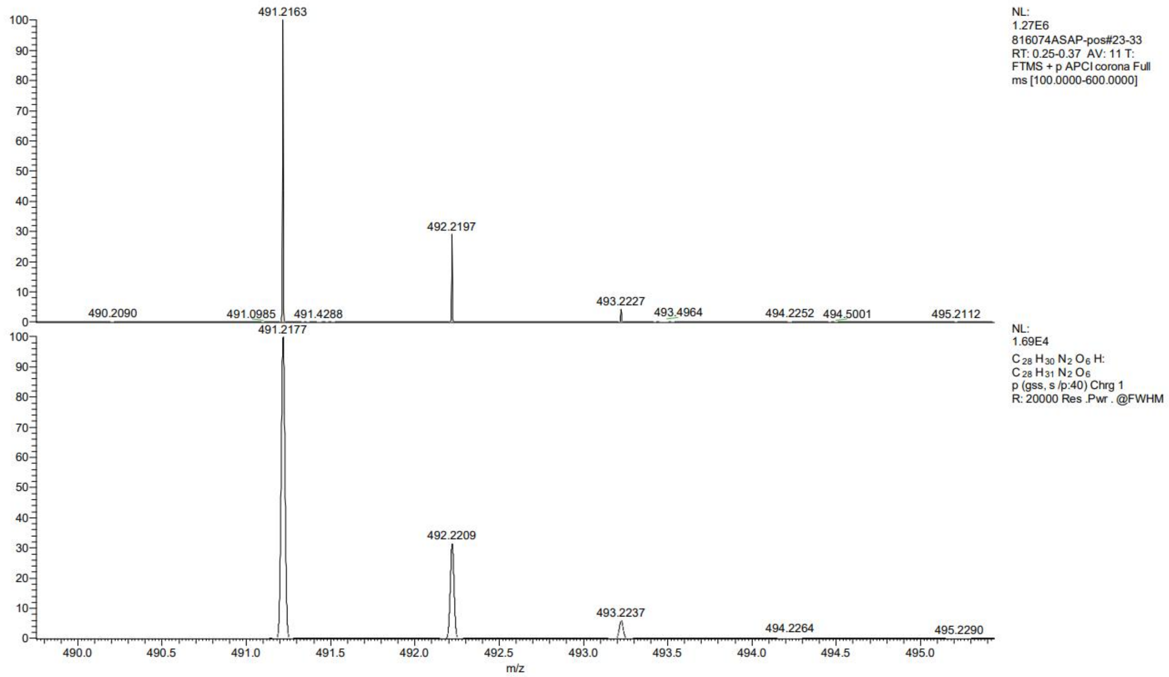

UV/VIS and Emission (CH_2Cl_2, 20 °C)

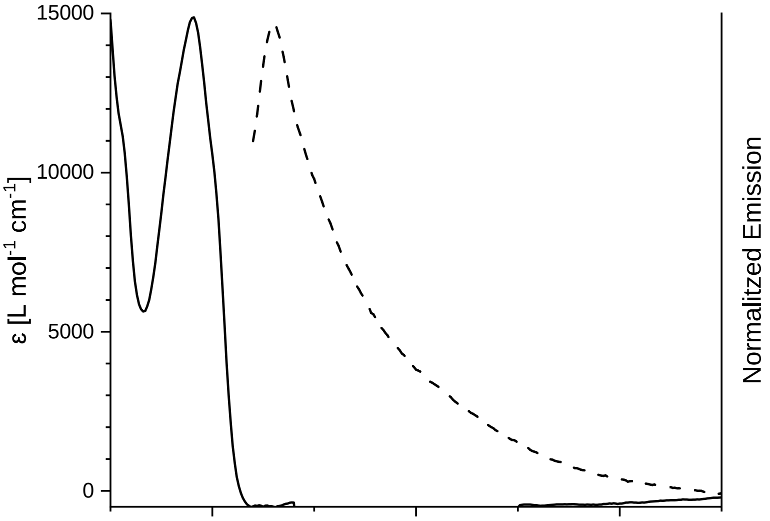

IR

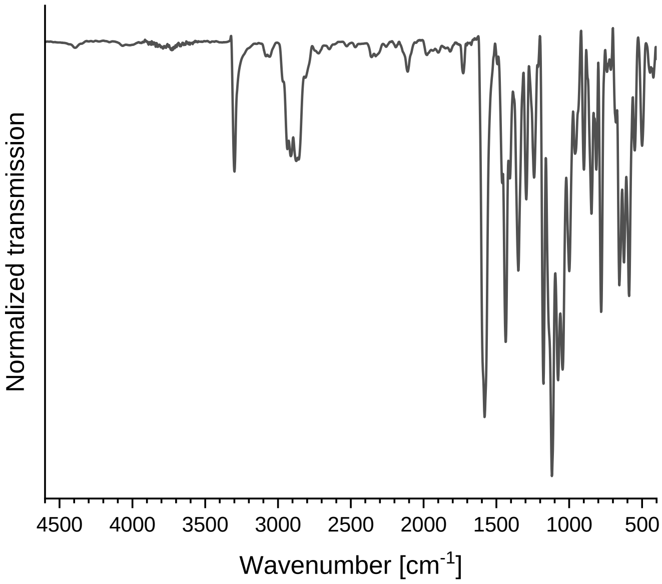
Normalized transmission
4500
4000
3500
3000
2500
2000
1500
1000
500
Wavenumber [cm-1]

Synthesis of **3**

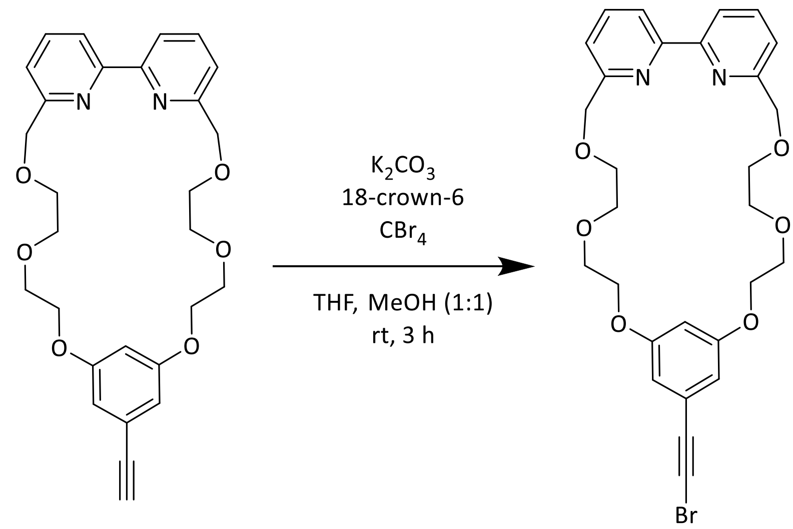

11 **3**, 95%

11 (66 mg, 135 µmol, 1.0 equiv) was dissolved in THF (3 mL) and MeOH (3 mL) and the solution was degassed by purging with argon for 10 min. Then K_2CO_3 (93 mg, 673 µmol, 5.0 equiv) and 18-Crown-6 (107 mg, 404 µmol, 3.0 equiv) were added and the reaction mixture was degassed by purging with argon for 5 min. Afterwards CBr_4 (89.2 mg, 269 µmol, 2.0 equiv) was added and the reaction mixture was stirred for 3 h at room temperature. The crude reaction mixture was then plugged over SiO_2, eluted with acetone and the solvent was removed *in vacuo*. **3** (73 mg, 128 µmol, 95%) was isolated as a white solid after purification by column chromatography (SiO_2, petroleum ether (40 – 60 °C):acetone (2:1 to 1:1)).

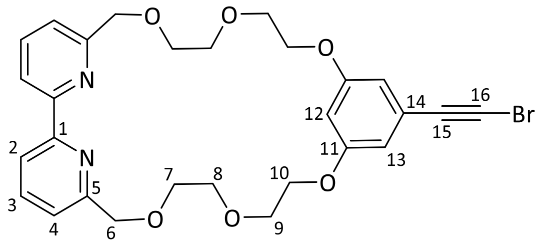

^{1}H-NMR (600 MHz, Chloroform-*d*, 298 K, δ/ppm): 8.28 (d, $^3J_{HH}$ = 7.7 Hz, 2H, H2), 7.63 (app. t, $^3J_{HH}$ =7.7 Hz, 2H, H3), 7.39 (d, $^3J_{HH}$ = 7.7 Hz, 2H, H4), 6.52 (d, $^4J_{HH}$ = 2.3 Hz, 2H, H13), 6.03 (app. s, 1H, H12), 4.78 (s, 4H, H6), 3.86 – 3.83 (m, 8H, H7, H10), 3.75 – 3.72 (m, 8H, H8, H9).

^{13}C{^{1}H}-NMR (151 MHz, Chloroform-*d*, 298 K, δ/ppm): 159.67 (C11), 158.16 (C5), 155.69 (C1), 137.41 (C3), 123.70 (C14), 121.96 (C4), 119.92 (C2), 110.38 (C13), 103.64 (C12), 80.18 (C15), 74.23 (C6), 71.17 (C8 or C9), 70.37 (C7 or C10), 69.51 (C8 or C9), 67.55 (C7 or C10), 49.43 (C16).

HRMS (APCI, +): *m/z* calc. $[M+H]^+$: 569.1282, found: 569.1277.

^{1}H-NMR (600 MHz, Chloroform-*d*, 298 K)

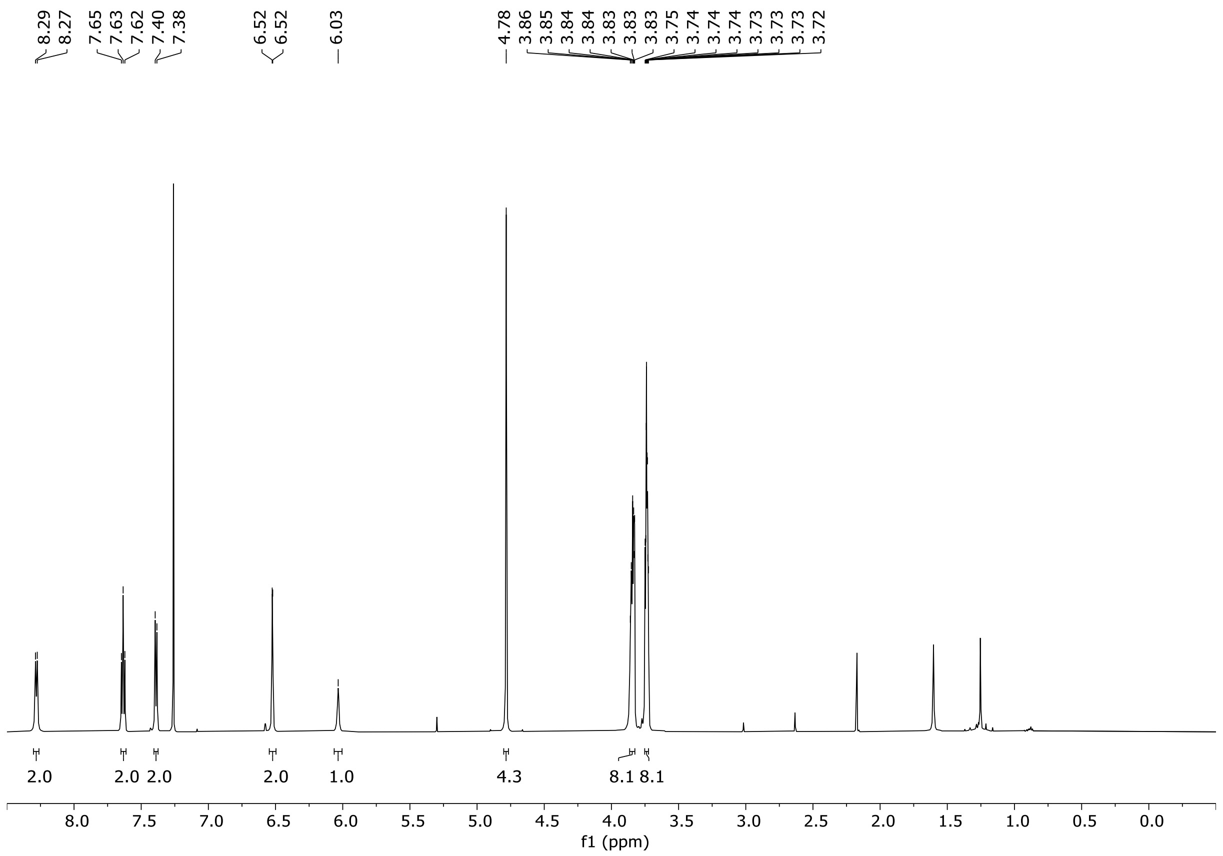

^{13}C{^{1}H}-NMR (151 MHz, Chloroform-*d*, 298 K)

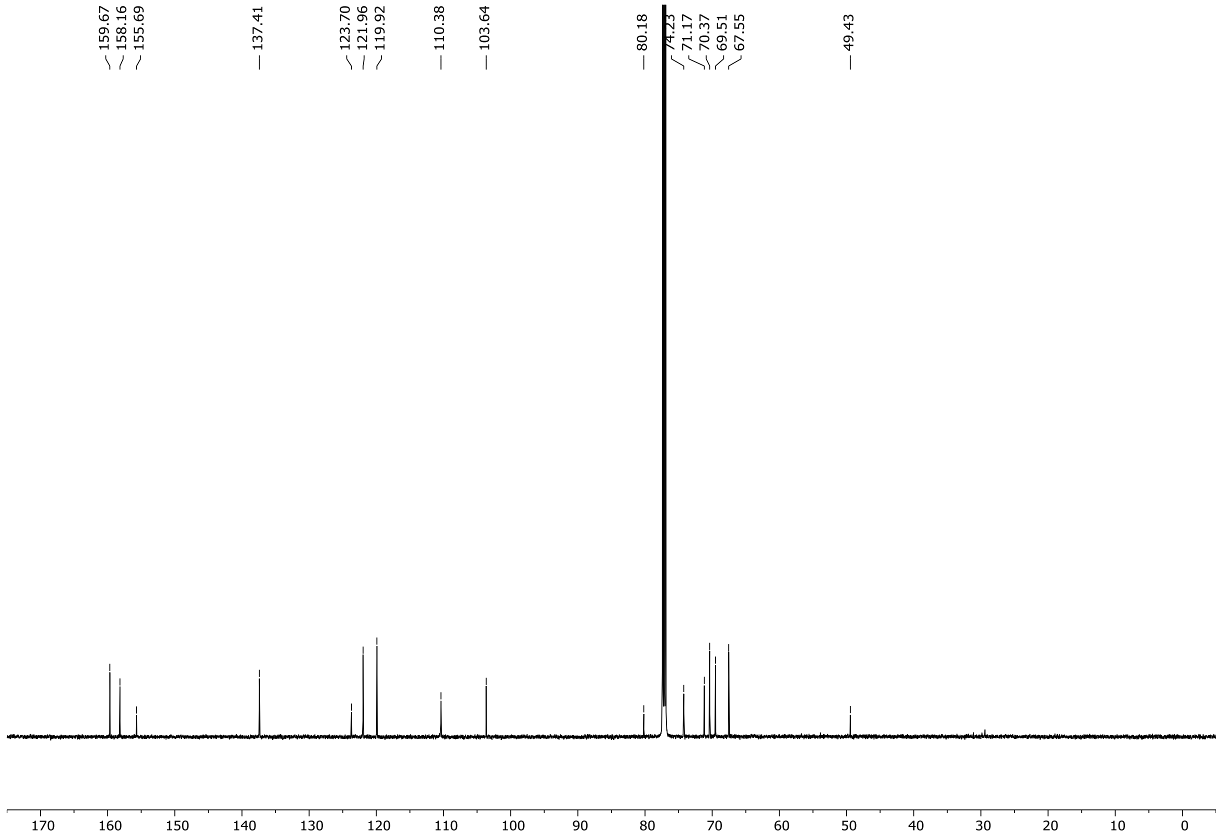

DEPTQ (151 MHz, Chloroform-*d*, 298 K)

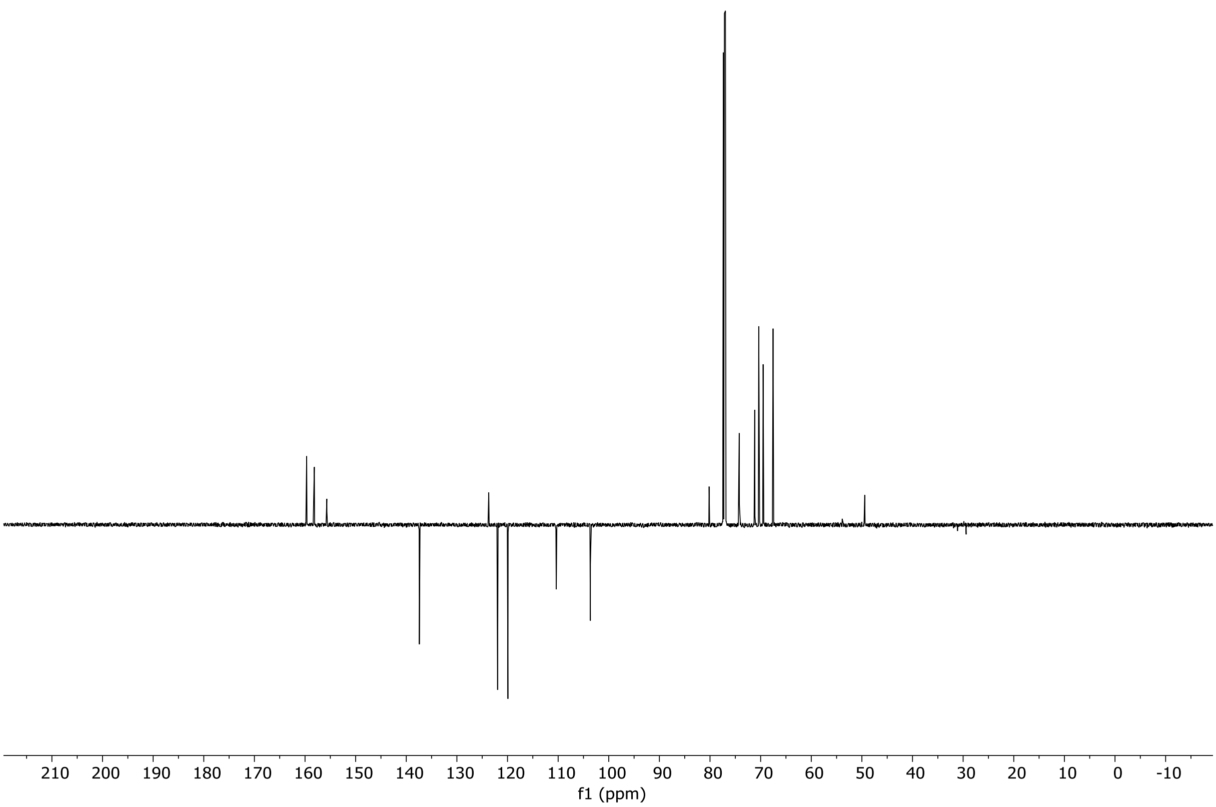

HSQC (600 Hz, Chloroform-*d*, 298 K)

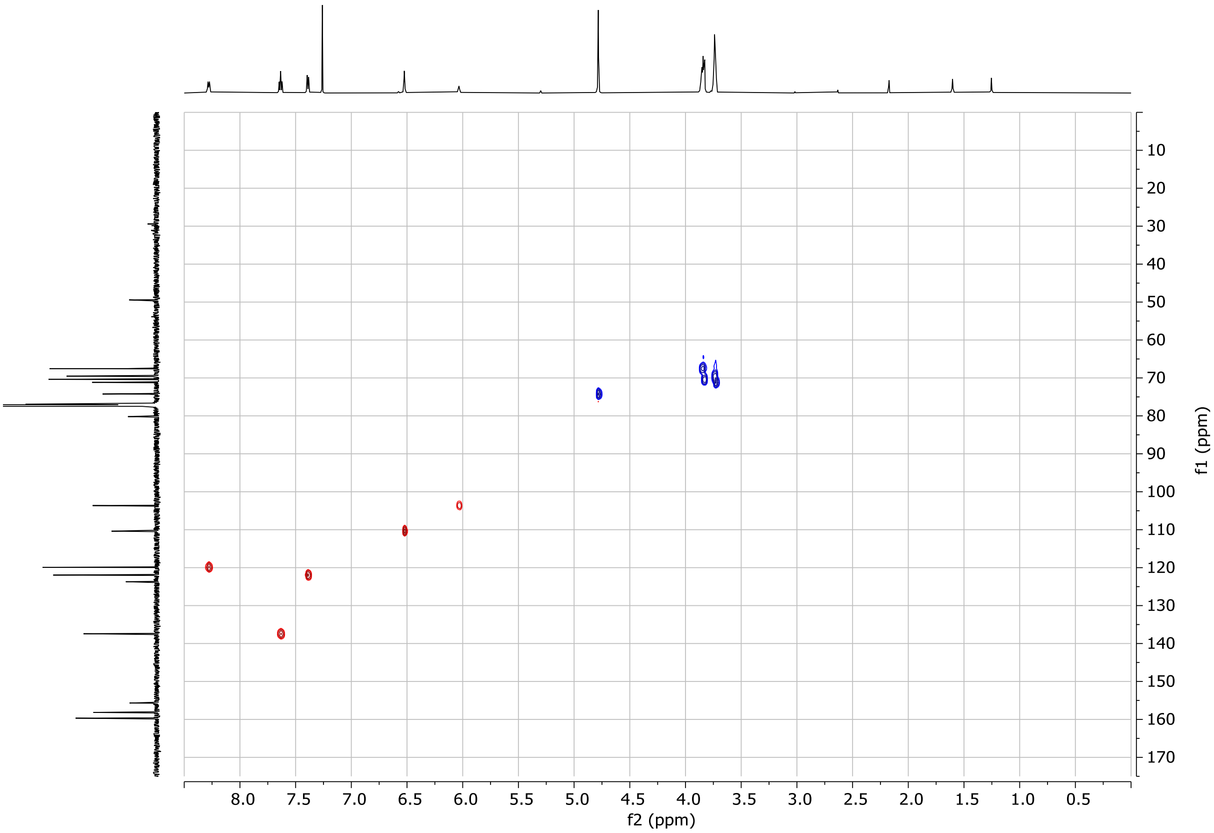

HMBC (600 Hz, Chloroform-*d*, 298 K)

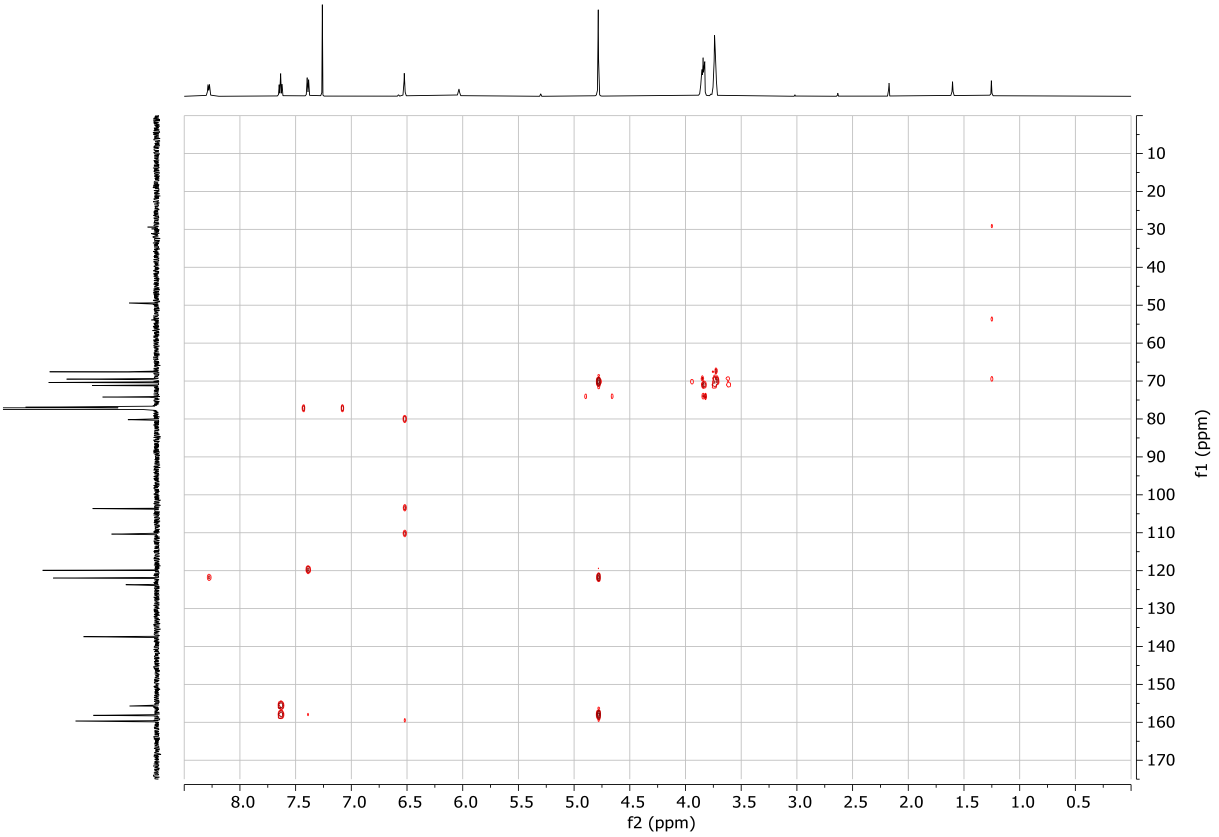

COSY (600 Hz, Chloroform-*d*, 298 K)

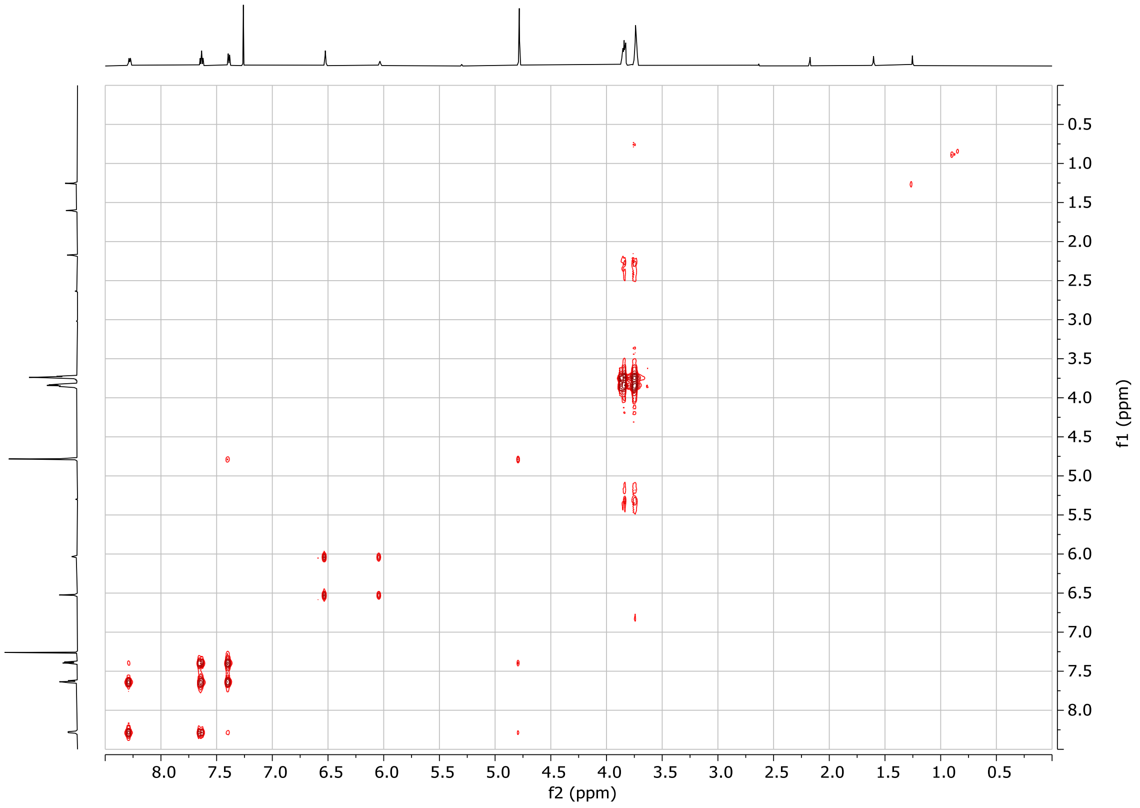

HRMS (APCI, +)

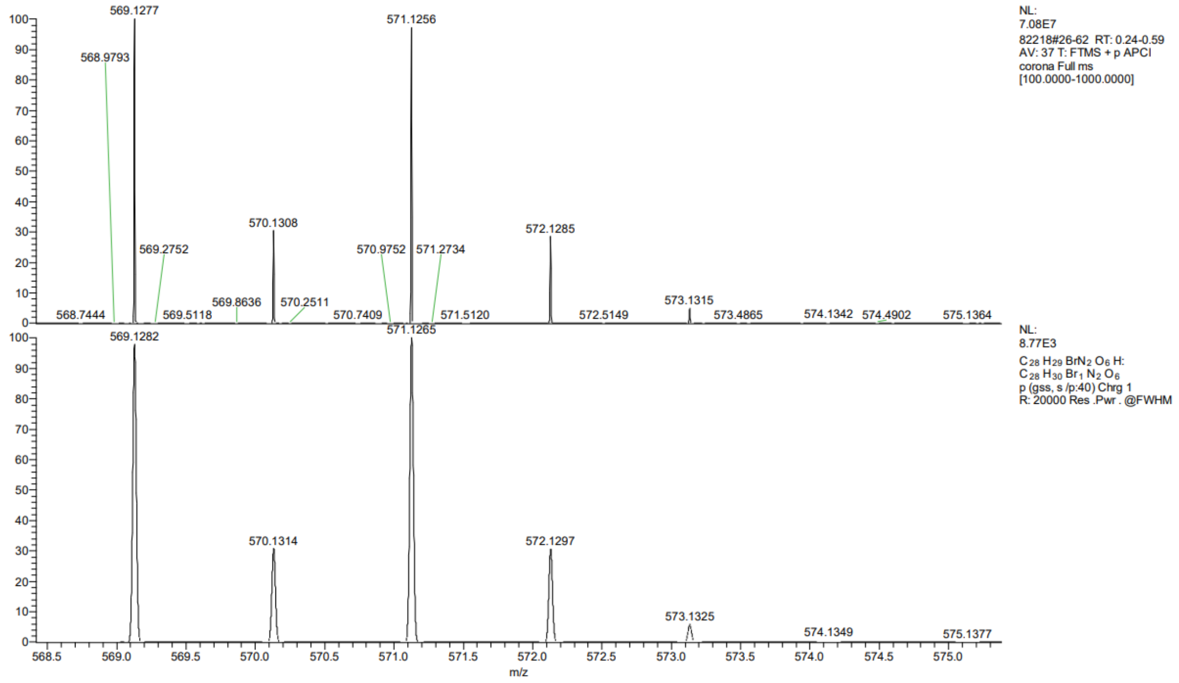

NL:
7.08E7
82218#26-62 RT: 0.24-0.59
AV: 37 T: FTMS + p APCI
corona Full ms
[100.0000-1000.0000]
NL:
8.77E3
$C_{28}H_{29}BrN_2O_6$ H:
$C_{28}H_{30}Br_1N_2O_6$
p (gss, s /p:40) Chrg 1
R: 20000 Res .Pwr . @FWHM
569.1277
571.1256
568.9793
570.1308
572.1285
569.2752
570.9752
571.2734
569.8636
570.2511
573.1315
568.7444
569.5118
570.7409
571.5120
572.5149
573.4865
574.1342
574.4902
575.1364
569.1282
571.1265
570.1314
572.1297
573.1325
574.1349
575.1377
m/z

IR

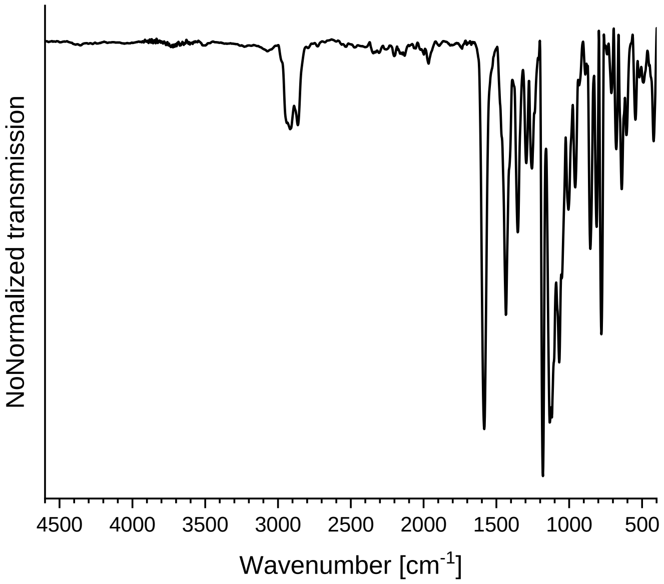

NoNormalized transmission
Wavenumber [cm^{-1}]

Synthesis of **13**.

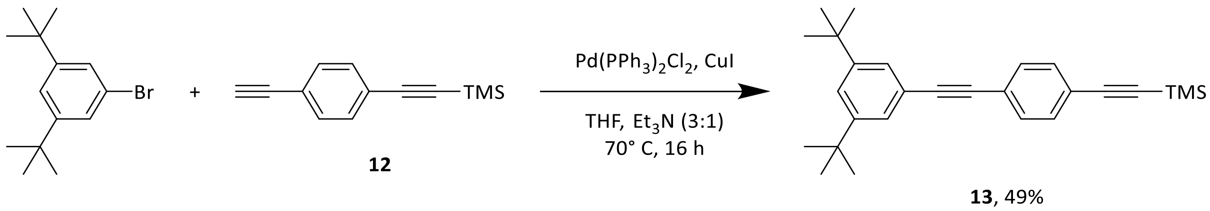

1-Bromo-3,5-di-tert-butylbenzene (489 mg, 1.82 mmol, 1.2 equiv.) and literature known **12**[8] (300 mg, 1.51 mmol, 1.0 equiv.) were placed in to a pressure tube, dissolved in THF (6 mL) and Et_3N (2 mL) and placed under inert argon atmosphere by purging with argon for 10 minutes. Then copper(I) iodide (14.4 mg, 75.6 µmol, 0.05 equiv.) and bis(triphenylphosphine) palladium(II) dichloride (53 mg, 75.6 µmol, 0.05 equiv.) were added and the reaction mixture was purged with argon for 5 more minutes. The reaction mixture was placed into the preheated heating bath at 70°C for 16 h. The crude reaction mixture was filtered over silica gel (SIO_2) and eluted with EtOAc and concentrated *in vacuo*. **13** (289 mg, 747 mmol, 49%) was obtained as a white solid after purification by column chromatography (SiO_2, cyclohexane, 100%).

TLC (SiO_2, cyclohexane, 100%, UV_{254nm}): R_f = 0.23.

^{1}H-NMR (500 MHz, Chloroform-*d*, 298 K, δ/ppm 7.49 – 7.46 (m, 2H), 7.45 – 7.42 (m, 2H), 7.41 (t, $^4J_{HH}$ = 1.9 Hz, 1H), 7.38 (d, $^4J_{HH}$ = 1.9 Hz, 2H), 1.34 (s, 18H), 0.26 (s, 9H).

^{13}C{^{1}H}-NMR (126 MHz, Chloroform-*d*, 298 K, δ/ppm): 151.1, 132.0, 131.5, 126.0, 123.8, 123.2, 122.8, 122.1, 104.9, 96.2, 92.7, 87.9, 35.0, 31.5, 0.1.

HRMS (ESI, +): *m/z* calc. $[M-H]^+$: 493.1475, found: 493.1470.

^{1}H-NMR (500 MHz, Chloroform-*d*, 298 K)

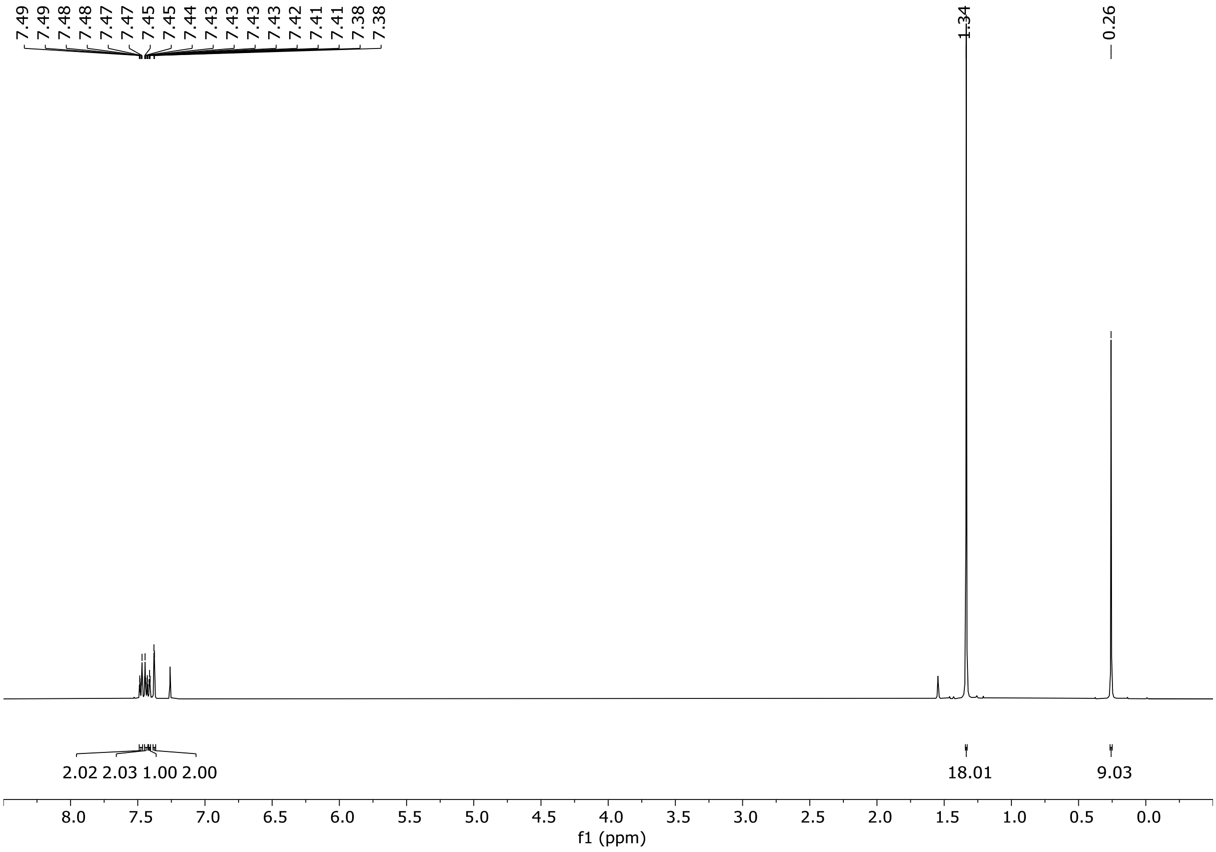

^{13}C{^{1}H}-NMR (126 MHz, Chloroform-*d*, 298 K)

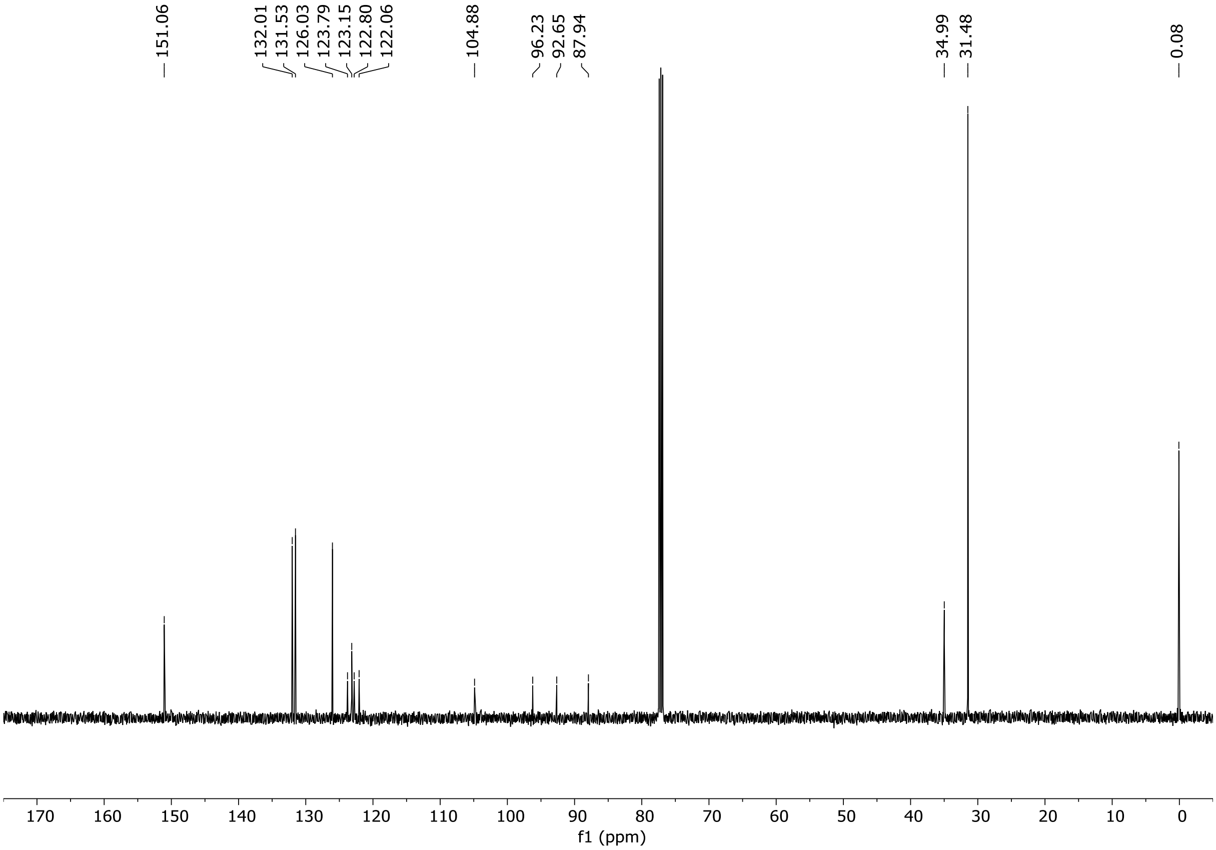

HRMS (ESI, +)

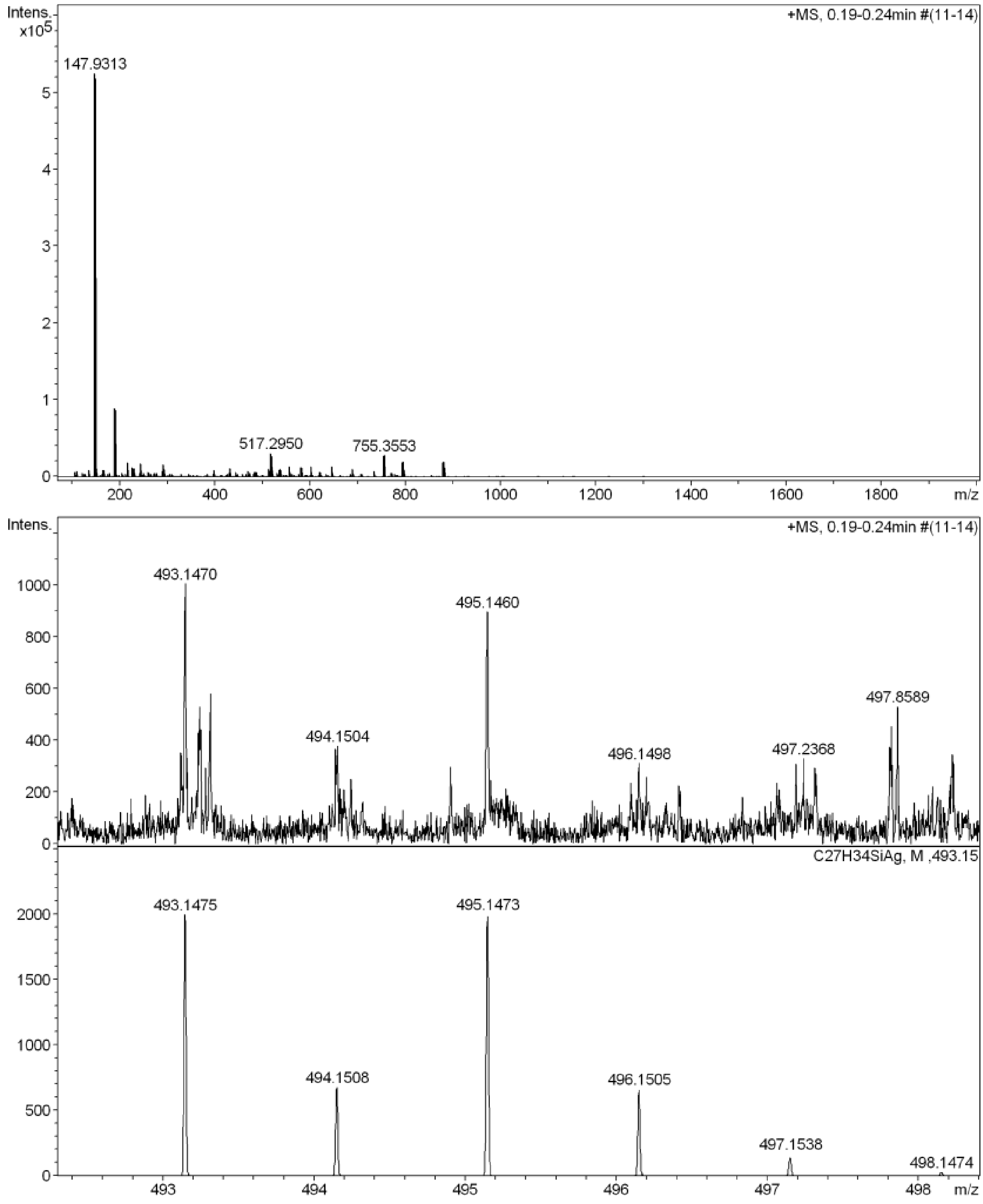

Intens.
x10^5
+MS, 0.19-0.24min #(11-14)
147.9313
517.2950
755.3553
m/z
Intens.
+MS, 0.19-0.24min #(11-14)
493.1470
495.1460
494.1504
496.1498
497.2368
497.8589
C27H34SiAg, M ,493.15
493.1475
495.1473
494.1508
496.1505
497.1538
498.1474
m/z

Synthesis of **2**.

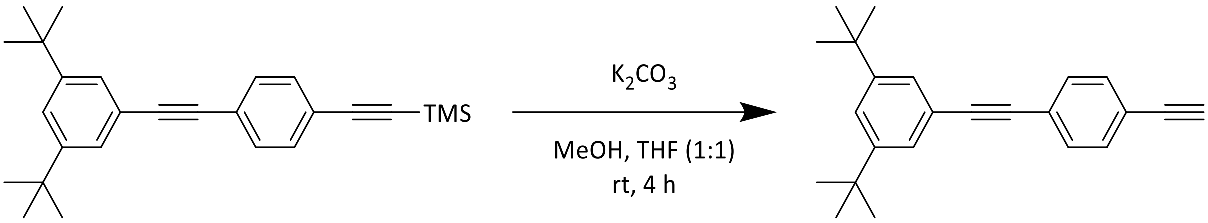

13 **2**, quant.

13 (147 mg, 380 µmol, 1.0 equiv.) was dissolved in THF (5 mL) and MeOH (5 mL) and the solution was degassed by purging with argon for 10 min. Potassium carbonate (210 mg, 1.52 mmol, 4.0 equiv.) was added, the reaction mixture was degassed by purging with argon for 5 more minutes and then stirred for 4 h at rt. Afterwards NH_4Cl (sat.) was added and the mixture was diluted with EtOAc. The organic layer was washed once with NH_4Cl and once with water, dryed over Na_2SO_4 and the solvent was removed *in vacuo* to obtain **2** (102 mg, 388 µmol, quantitative) as a white solid.

TLC (SiO_2, cyclohexane, 100%, UV_{254nm}): R_f = 0.20.

^{1}H-NMR (500 MHz, Chloroform-*d*, 298 K, *δ*/ppm): 7.51 – 7.49 (m, 2H), 7.48 – 7.45 (m, 2H), 7.42 (t, $^4J_{HH}$ =1.9 Hz, 1H), 7.38 (d, $^4J_{HH}$ =1.9 Hz, 2H), 3.17 (s, 1H), 1.34 (s, 18H).

^{13}C{^{1}H}-NMR (126 MHz, Chloroform-*d*, 298 K, *δ*/ppm): 151.1, 132.2, 131.6, 126.1, 124.2, 123.2, 122.0, 121.8, 92.7, 83.5, 78.9, 35.0, 31.5.

HRMS (ESI, +): *m/z* calcd. $[M+Ag]^+$: 421.1080, found: 421.1074.

UV/VIS (CH_2Cl_2, 20 °C) λ_{max} [nm] (ε [L·cm^{-1}·mol^{-1}]) : 320 (32.6·10^3), 301 (33.6·10^3).

Emission (CH_2Cl_2, 20 °C) λ_{max} [nm] (excitation [nm]) : 337 (300), 335 (300).

Quantum Yield ϕ_f (CH_2Cl_2, 20 °C) (excitation [nm]) : 81.4% (300).

^{1}H-NMR (500 MHz, Chloroform-*d*, 298 K)

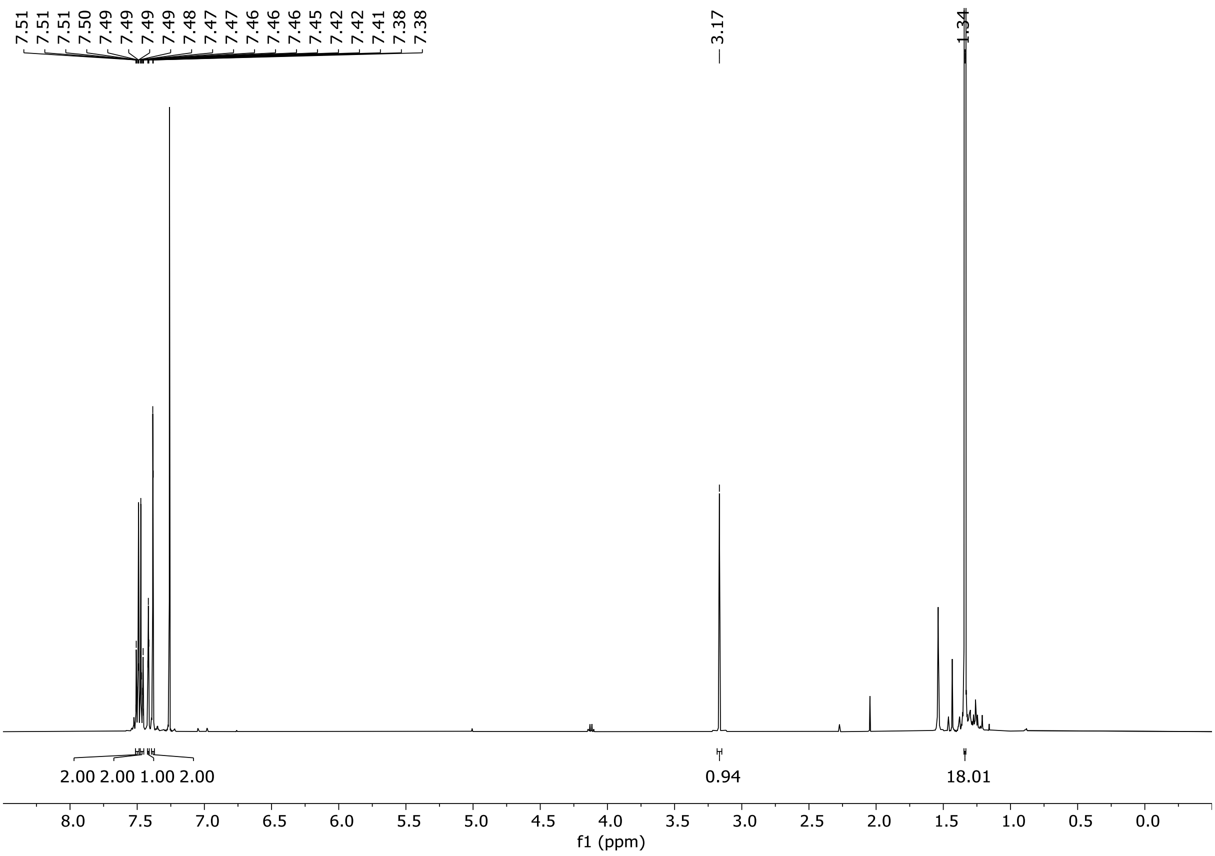

^{13}C{^{1}H}-NMR (126 MHz, Chloroform-*d*, 298 K)

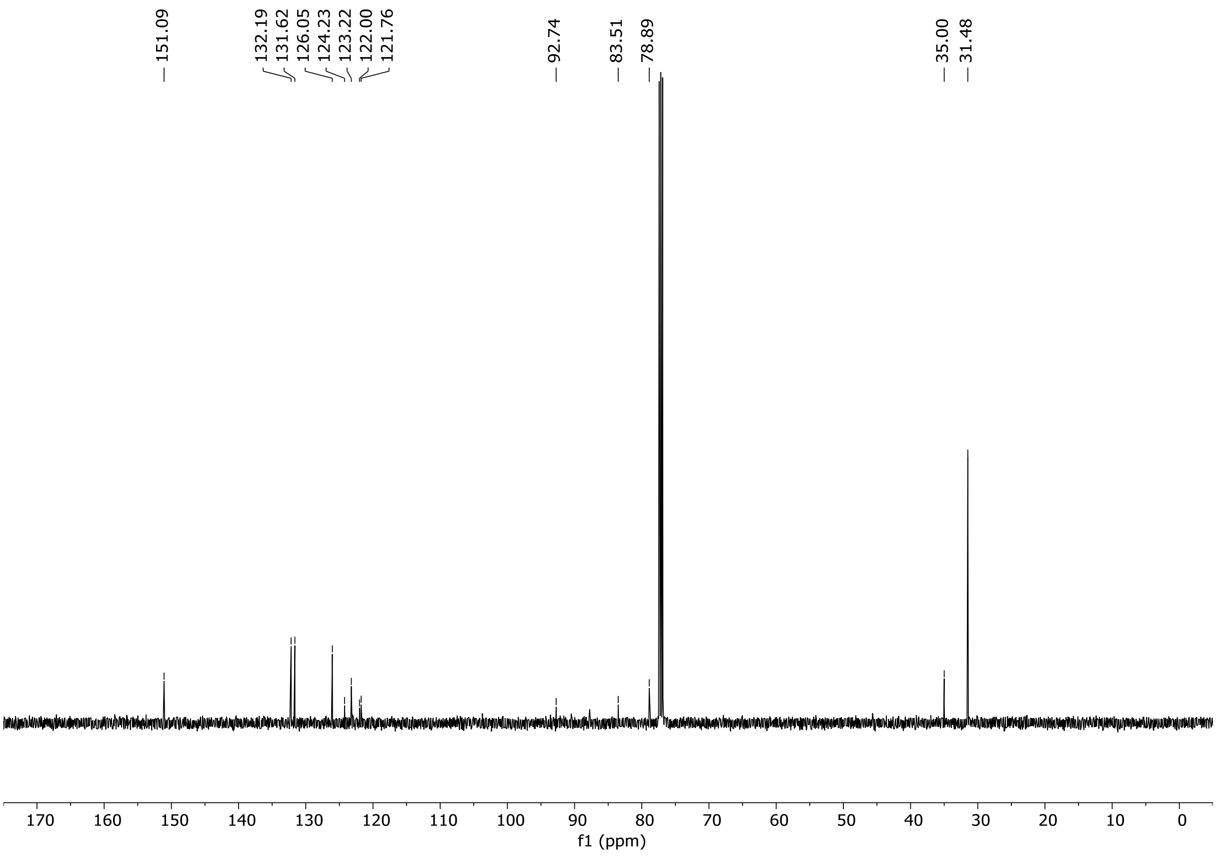

HRMS (ESI, +)

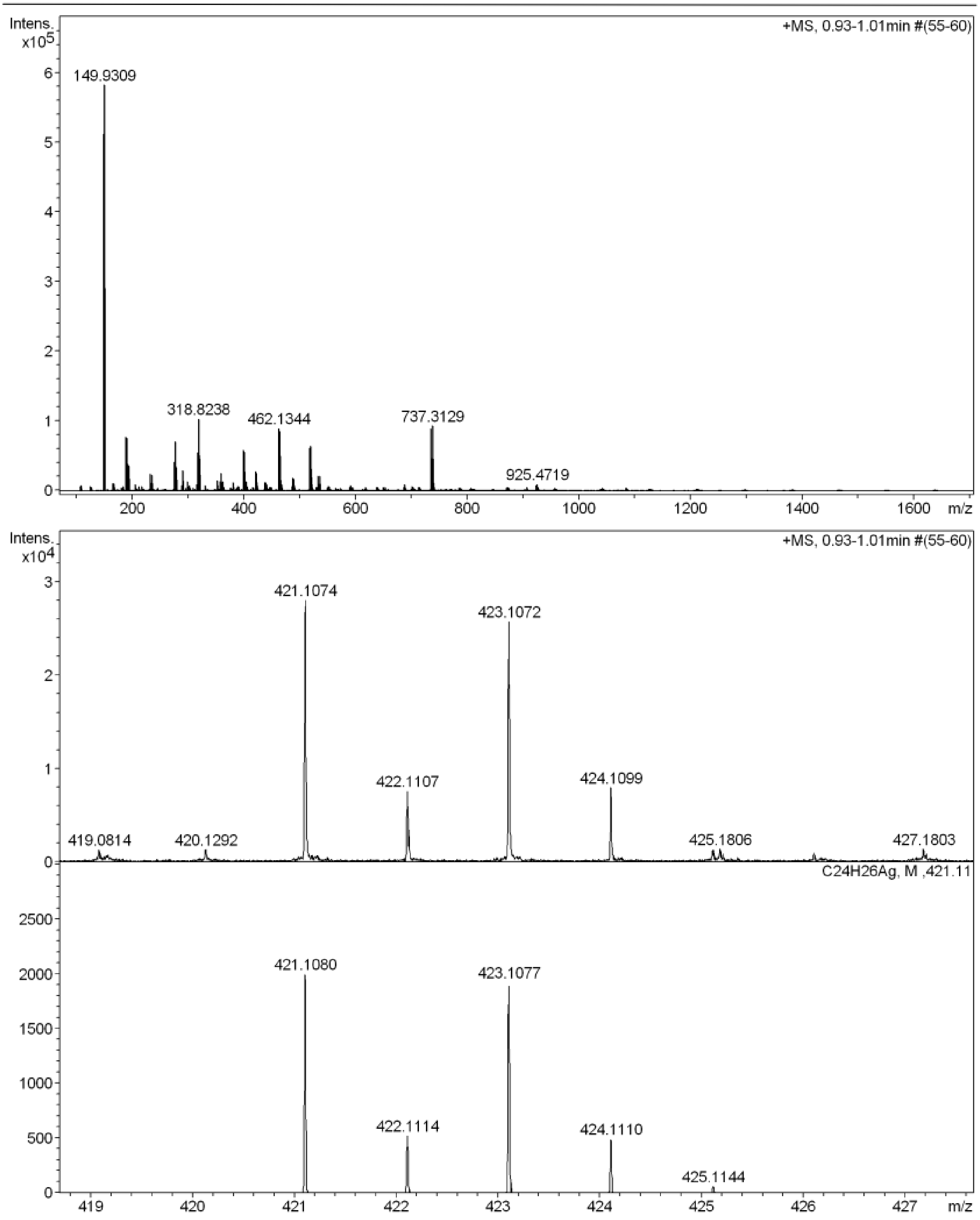

Intens.
x10^5
+MS, 0.93-1.01min #(55-60)
149.9309
318.8238
462.1344
737.3129
925.4719
m/z
Intens.
x10^4
+MS, 0.93-1.01min #(55-60)
421.1074
423.1072
422.1107
424.1099
419.0814
420.1292
425.1806
427.1803
C24H26Ag, M ,421.11
421.1080
423.1077
422.1114
424.1110
425.1144
m/z

UV/VIS and Emission (CH_2Cl_2, 20 °C)

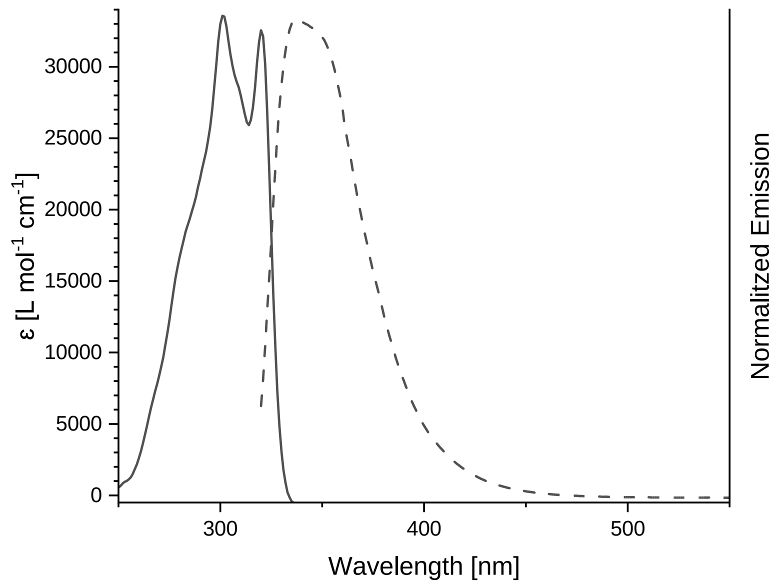

Synthesis of **1** and **4**.

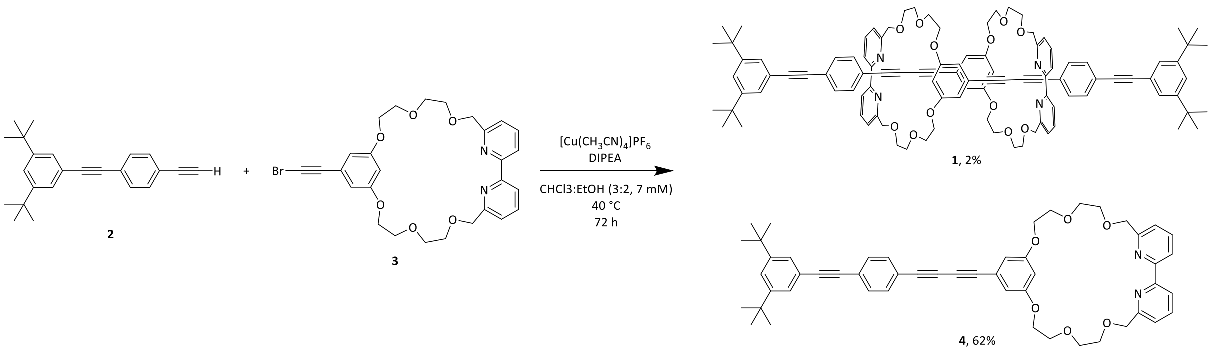

2 (22.1mg, 70.2 µmol, 1.0 equiv), **3** (40.0 mg, 70.2 µmol, 1.0 equiv) and tetrakis(acetonitrile)copper(I) hexafluorophosphatewere (19.6 mg, 52.7 µmol, 0.75 equiv) were placed under inert argon atmosphere. Chloroform (6 mL) and EtOH (4 mL) were separately placed under inert argon atmosphere by purging with argon for 20 minutes and then added to the reaction mixture which was further degassed by purging with argon for 10 more minutes followed by striiring for 30 minutes at room temperature. Then diisopropylethylamine (DIPEA, 13.6 mg, 105 µmol, 0.018 mL, 1.5 equiv), placed under inert argon atmosphere by purging with argon for 20 minutes, was added to the reaction mixture which was sealed and placed into a preheated heating bath at 40 °C for 72 h. The reaction mixture was then diluted with EtOAc and washed once with saturated EDTA-NH_3 solution, once with water and once with brine, dried over Na_2SO_4, filtered and the solvent was removed *in vacuo*. The crude was subject to purification by column chromatography (SiO_2, CH_2Cl_2:Acetone (15:1) + 1% Et_3N) which allowed to isolate two fractions. The first eluting fraction (after the front line) was further purified by precipitation with MeOH from CH_2Cl_2 and allowed to isolate **1** (1.1 mg, 0.7 µmol, 2%) as a white solid. The second fraction was further purified by precipitation with MeOH from CH_2Cl_2 and allowed to isolate **4** (35.0 mg, 43 µmol, 62%) as a white solid.

Characterization of **4**:

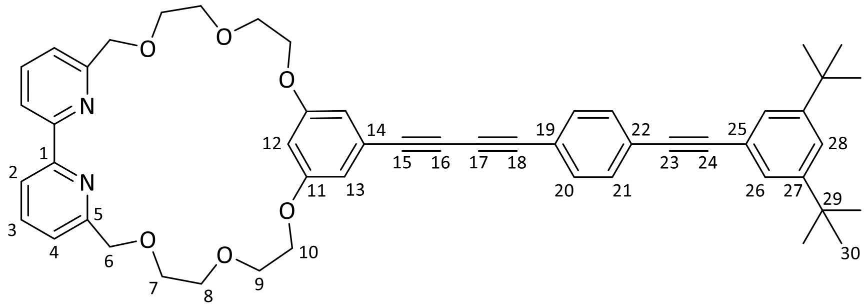

^{1}H-NMR (600 MHz, Chloroform-*d*, 298 K, δ/ppm): 8.29 (dd, $^3J_{HH}$ =7.7, $^4J_{HH}$ =1.1 Hz, 2H, H2), 7.65 (app. t, $^3J_{HH}$ = 7.7 Hz, 2H, H3), 7.52 – 7.48 (m, 4H, H20, H21), 7.42 (t, $^4J_{HH}$ = 1.8 Hz, 1H, H28), 7.40 (dd, $^3J_{HH}$ = 7.7, $^4J_{HH}$ = 1.1 Hz, 2H, H4), 7.38 (d, $^4J_{HH}$ = 1.8 Hz, 2H, H26), 6.61 (d, $^4J_{HH}$ = 2.3 Hz, 2H, H13), 6.07 (t, $^4J_{HH}$ = 2.3 Hz, 1H, H12), 4.79 (s, 4H, H6), 3.88 – 3.86 (m, 4H, H10), 3.85 – 3.83 (m, 4H, H7), 3.76 – 3.73 (m, 8H, H8, H9), 1.34 (s, 18H, H30).

^{13}C{^{1}H}-NMR (151 MHz, Chloroform-*d*, 298 K, δ/ppm): 159.61 (C11), 158.04 (C5), 155.58 (C1), 150.96 (C27), 137.29 (C3), 132.42 (C20 or C21), 131.59 (C20 or C21), 125.93 (C26), 124.49 (C19 or C22), 123.18 (C28), 122.56 (C14), 121.86 (C4), 121.79 (C25), 121.20 (C19 or C22), 119.81 (C2), 110.67 (C13), 104.26 (C12), 93.35 (C24), 87.69 (C18 or C23), 82.54 (C15), 81.24 (C18 or C23), 75.55 (C16 or C17), 74.14 (C6), 73.29 (C16 or C17), 71.07 (C8 or C9), 70.27 (C7), 69.36 (C8 or C9), 67.46 (C10), 34.87 (C29), 31.35 (C30).

HRMS (APCI, +): *m/z* calc. $[M+H]^+$: 803.4055, found: 803.4037.

UV/VIS (CH_2Cl_2, 20 °C) λ_{max} [nm] (ε [L·cm^{-1}·mol^{-1}]) : 361 (34.4·10^3), 339 (51.0·10^3), 293 (27.1·10^3).

Emission (CH_2Cl_2, 20 °C) λ_{max} [nm] (excitation [nm]) : 373 (320), 396(320).

Quantum Yield ϕ_f (CH_2Cl_2, 20 °C) (excitation [nm]) : 10.4% (335).

^{1}H-NMR (600 MHz, Chloroform-*d*, 298 K)

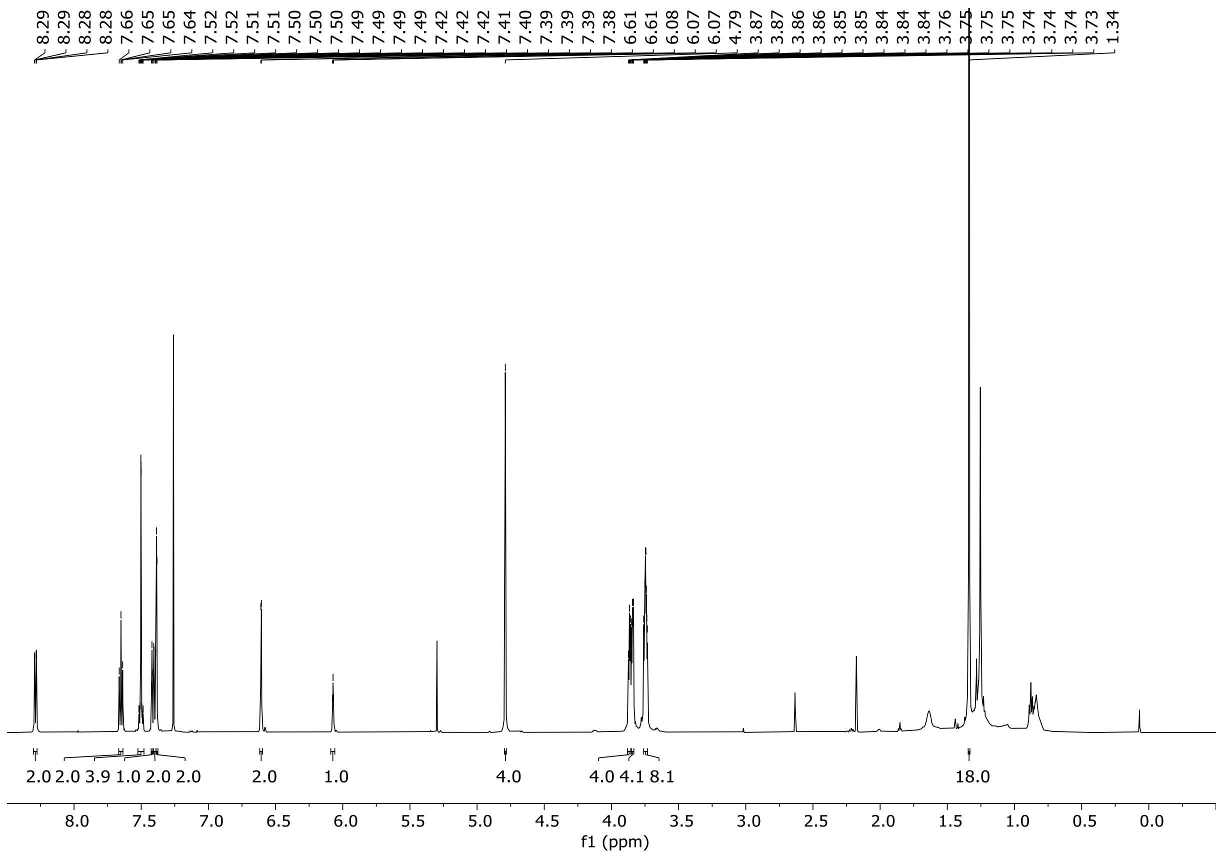

^{13}C{^{1}H}-NMR (151 MHz, Chloroform-*d*, 298 K)

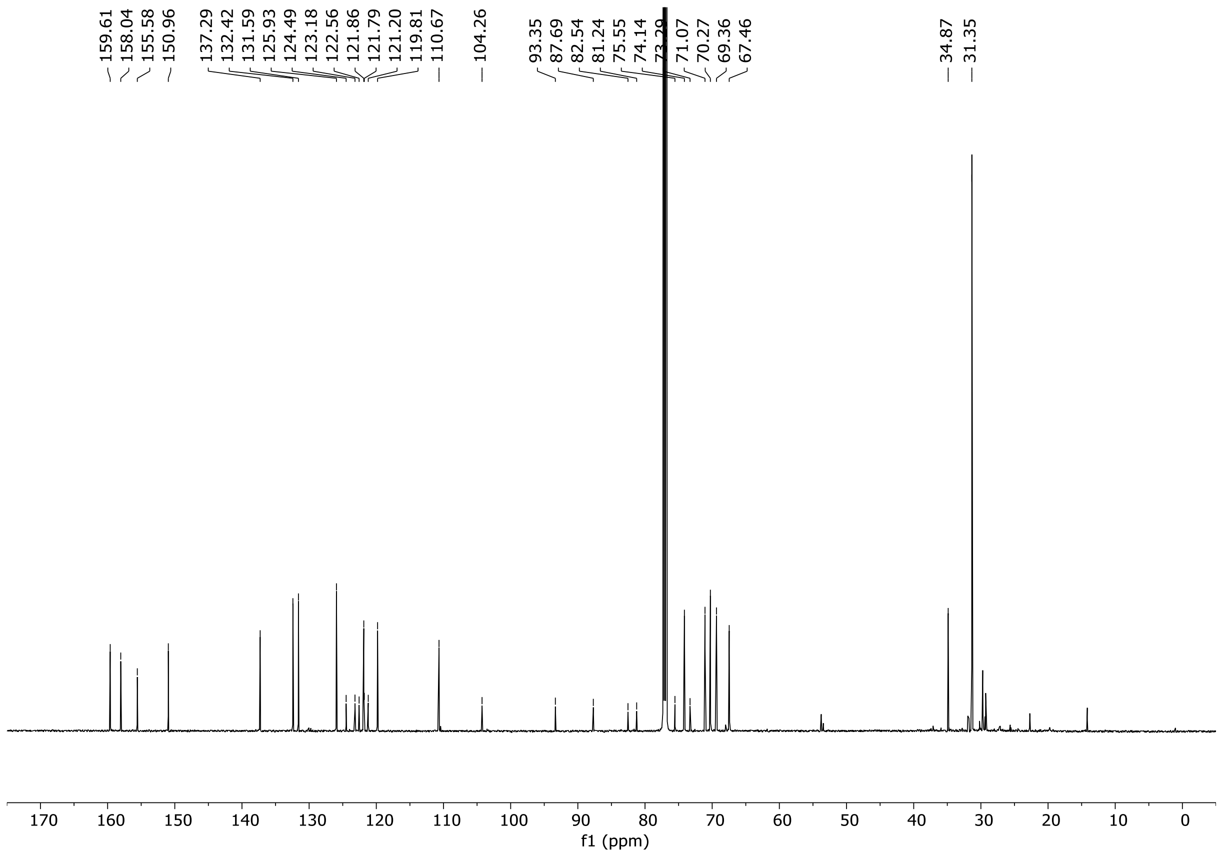

DEPTQ (151 MHz, Chloroform-*d*, 298 K)

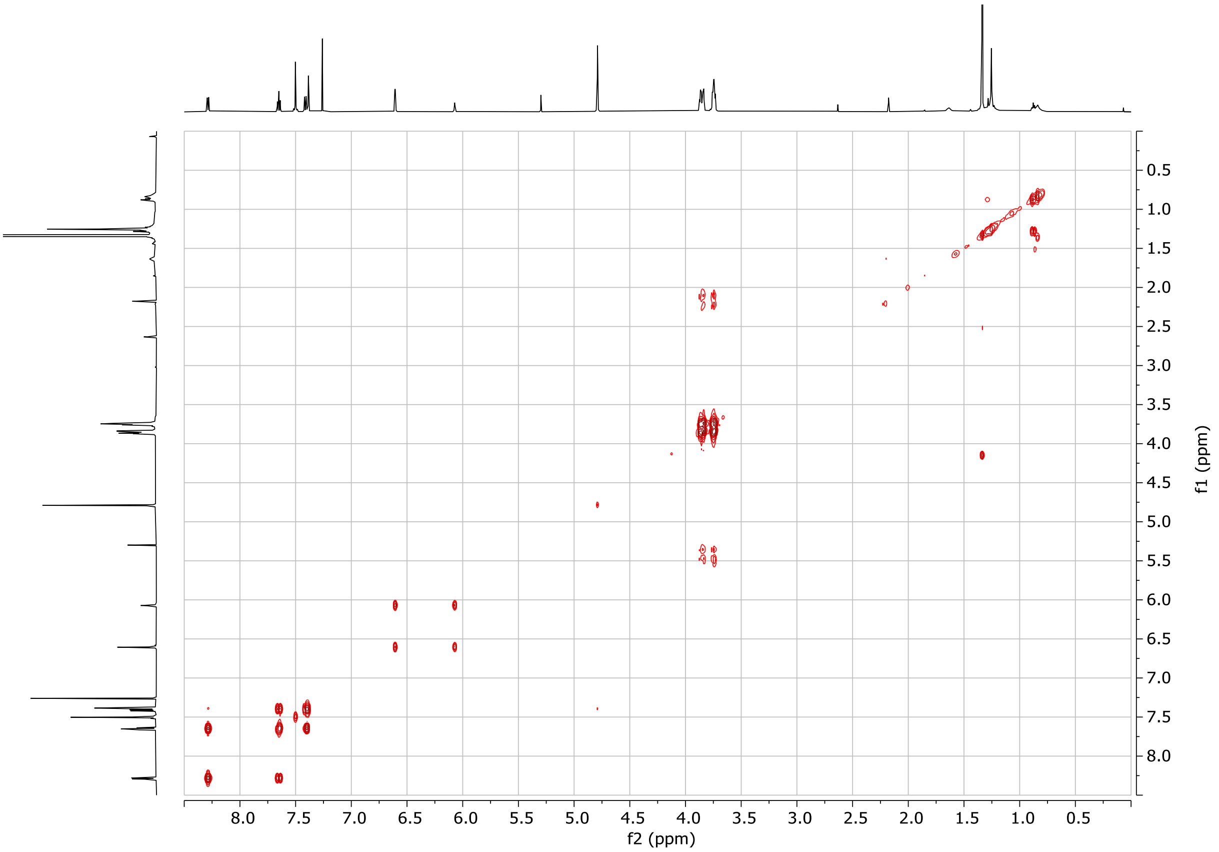

HSQC (600 Hz, Chloroform-*d*, 298 K)

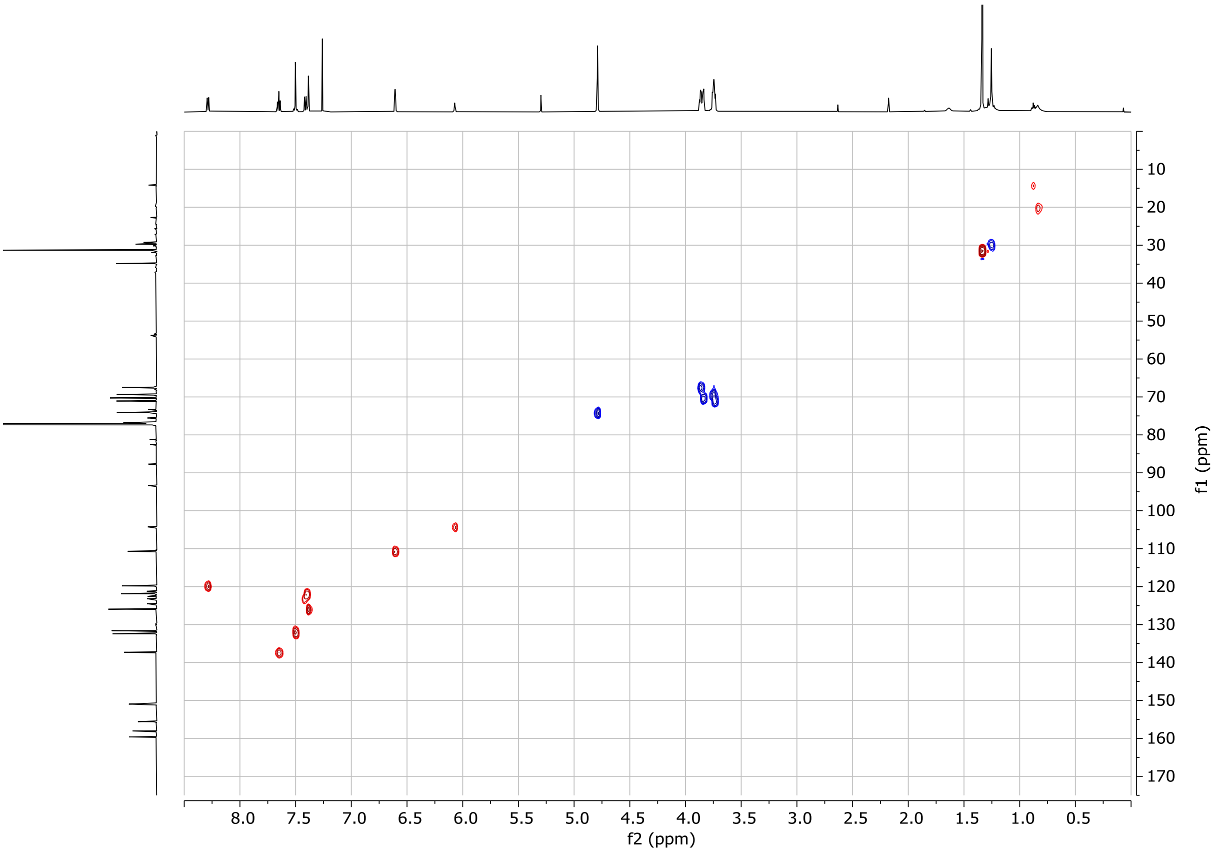

HMBC (600 Hz, Chloroform-*d*, 298 K)

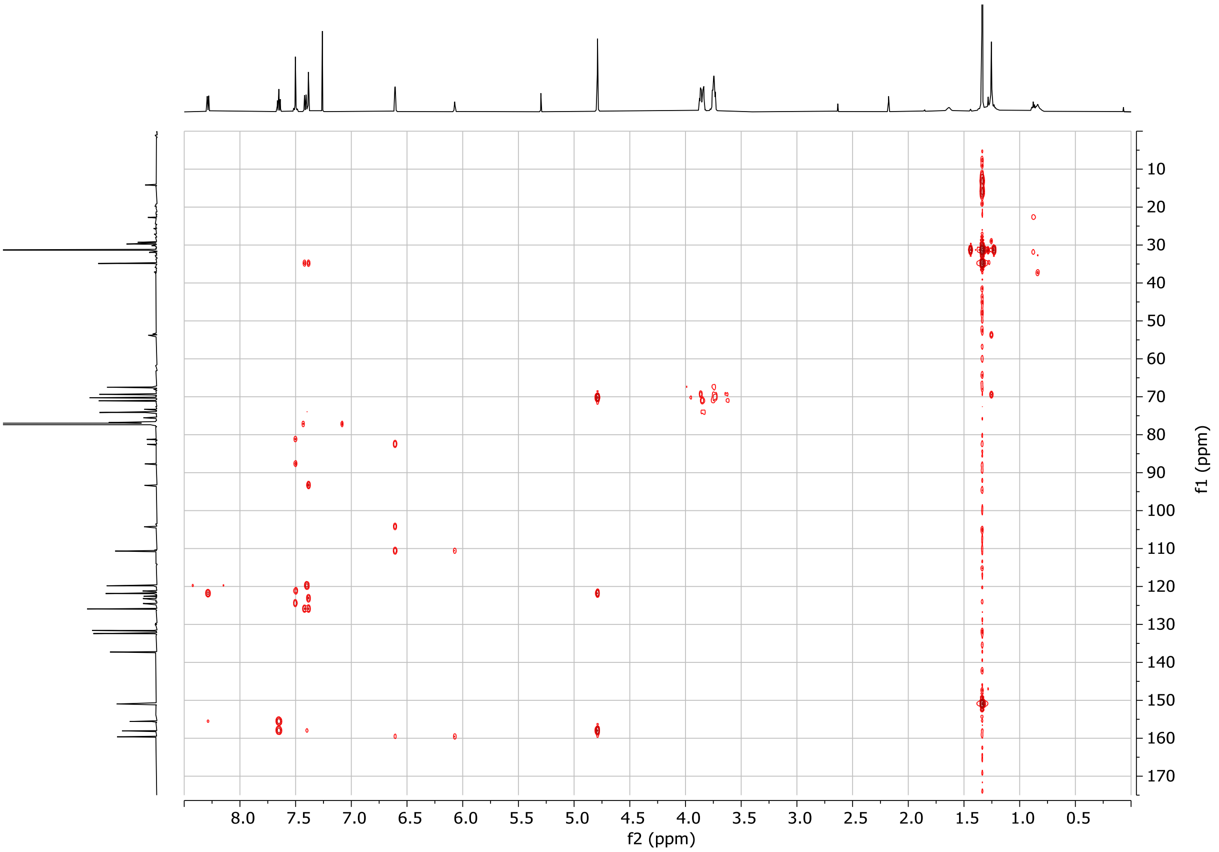

COSY (600 Hz, Chloroform-*d*, 298 K)

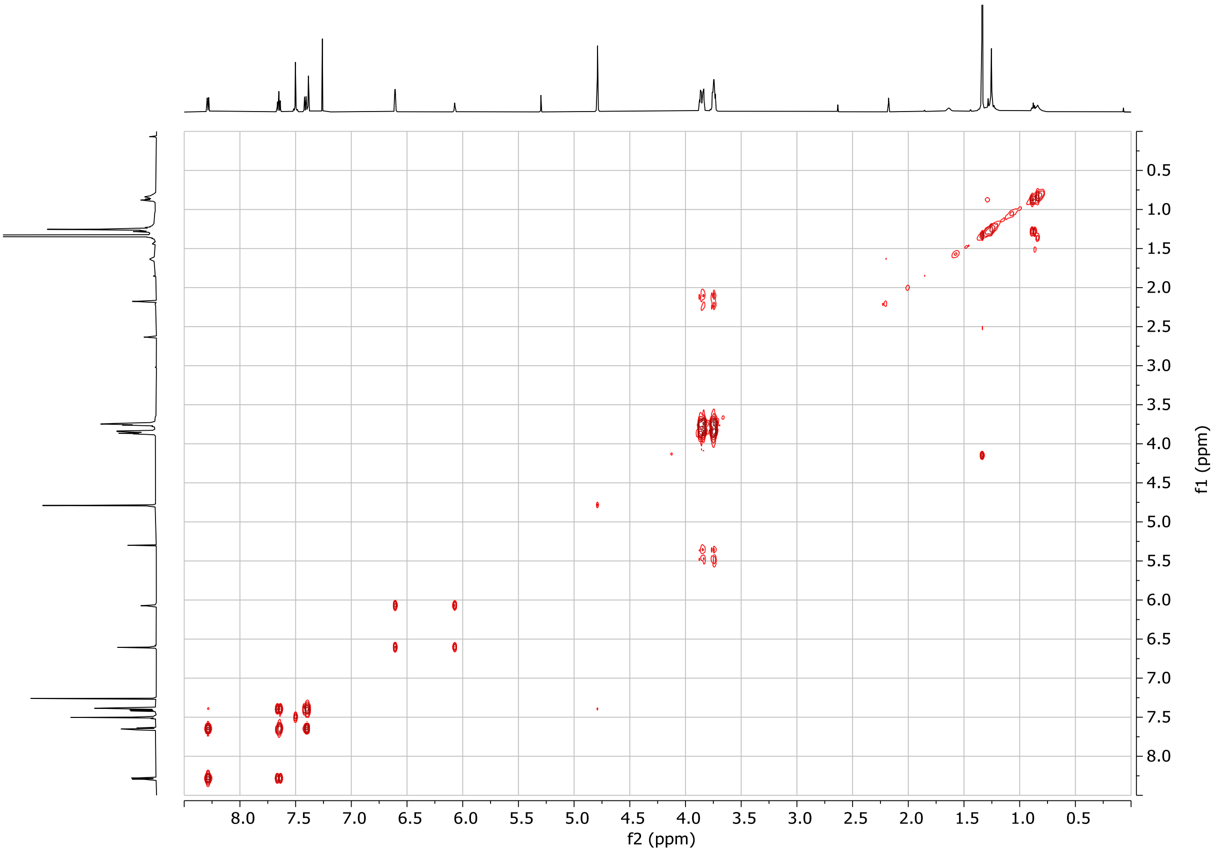

HRMS (APCI, +)

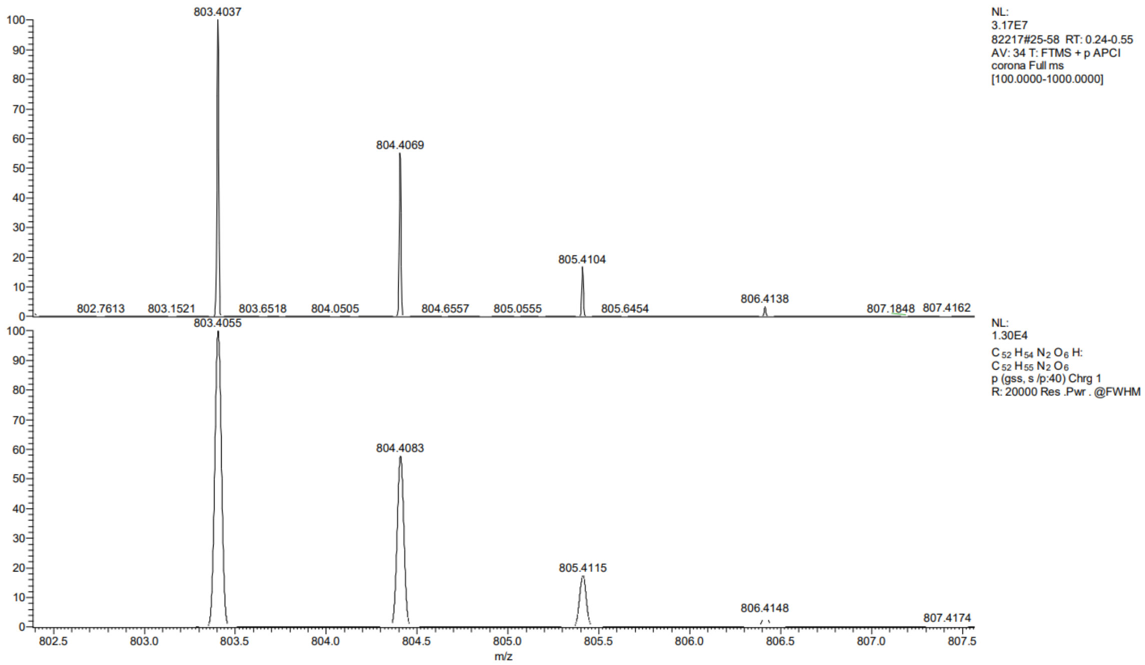

UV/VIS and Emission (CH_2Cl_2, 20 °C)

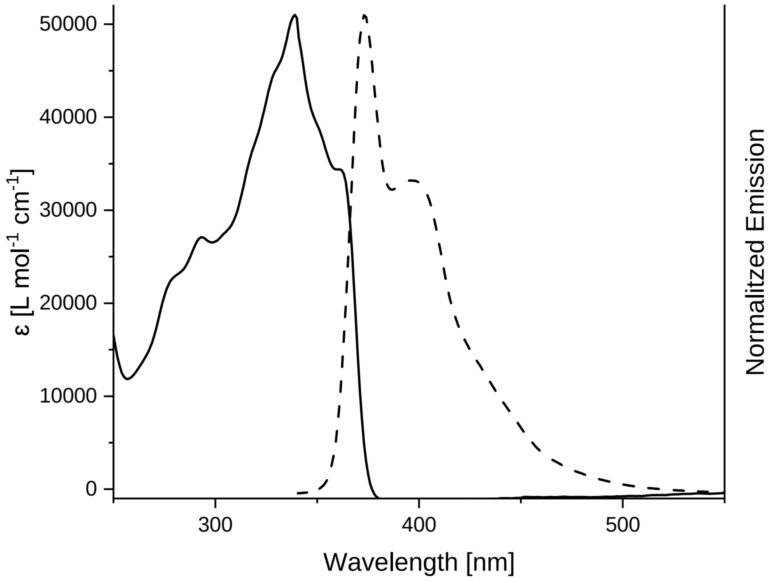

Characterization of **1**:

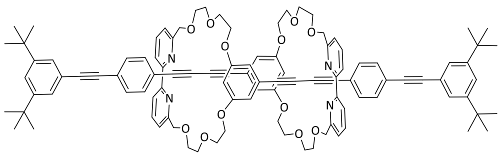

19

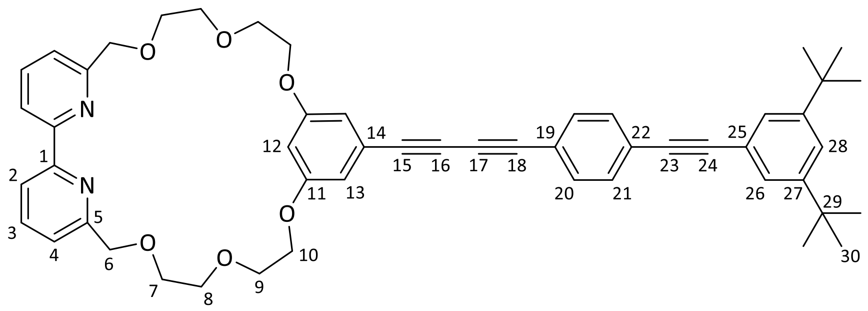

^{1}H-NMR (600 MHz, Chloroform-*d*, 298 K, δ/ppm): 7.90 (d, $^3J_{HH}$ = 7.3, 2H, H2), 7.37 (t, $^4J_{HH}$ = 1.8 Hz, 1H, H28), 7.21 (d, $^4J_{HH}$ = 1.8 Hz, 2H, H26), 7.17 – 7.13 (m, 2H, H3), 7.12 (d, $^3J_{HH}$ = 7.4, 2H, H4), 7.09 – 7.06 (m, 2H, H20), 7.02 – 6.98 (m, 2H, H21), 6.09 (d, $^4J_{HH}$ = 2.4 Hz, 2H, H13), 6.07 (t, $^4J_{HH}$ = 2.4 Hz, 1H, H12), 4.73 (dd, $^2J_{HH}$ = 11.7, $^4J_{HH}$ = 2.3 Hz, 2H, H6), 4.59 (dd, $^2J_{HH}$ = 11.7, $^4J_{HH}$ = 2.3 Hz, 2H, H6), 4.02 – 3.96 (m, 4H, H7, H10), 3.91 – 3.87 (m, 2H, H10), 3.84 – 3.79 (m, 4H, H8, H9), 3.77 – 3.68 (m, 6H, H7, H8, H9), 1.39 (s, 18H, H30).

The integrals are symmetry reduced.

^{13}C{^{1}H}-NMR (151 MHz, Chloroform-*d*, 298 K, δ/ppm): 158.69 (C11), 155.95 (C5), 155.90 (C1), 150.48 (C27), 136.46 (C3), 131.60 (C21), 131.44 (C20), 126.11 (C26), 124.17 (C22), 123.40 (C25), 122.83 (C4), 122.41 (C28), 122.39 (C14), 120.59 (C19), 120.37 (C2), 110.23 (C13), 104.41 (C12), 92.54 (C24), 89.04 (C23), 82.54 (C15), 81.37 (C18), 75.52 (C16 or C17), 75.43 (C6), 73.35 (C16 or C17), 70.79 (C8), 69.93(C9), 68.89(C7), 67.74(C10), 35.01 (C29), 31.62 (C30).

HRMS (APCI, +): *m/z* calc. $[M+H]^+$: 1605.8037, found: 1605.8007.

Tandem HRMS (APCI, +): *m/z* found: 1518, 1249, 983, 803 , 759, 715, 627.

UV/VIS (CH_2Cl_2, 20 °C) λ_{max} [nm]: 294, 324 shoulder, 344, 368 (normalized spectrum).

Emission (CH_2Cl_2, 20 °C) λ_{max} [nm] (excitation [nm]) : 375 (335) shoulder, 410 (335) shoulder, 450 (335) maximun.

Quantum Yield ϕ_f (CH_2Cl_2, 20 °C) (excitation [nm]) : 7.5% (335), 7.7% (340)

^{1}H-NMR (600 MHz, Chloroform-*d*, 298 K)

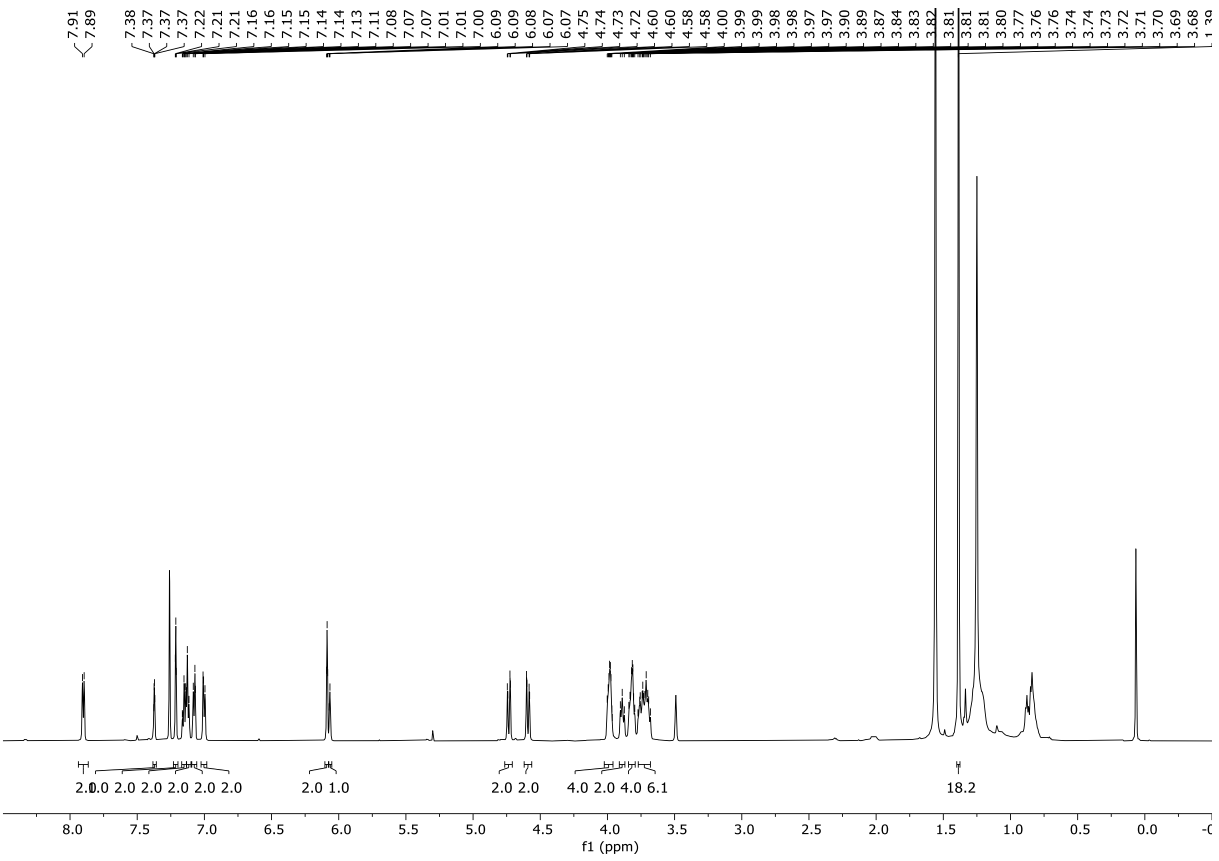

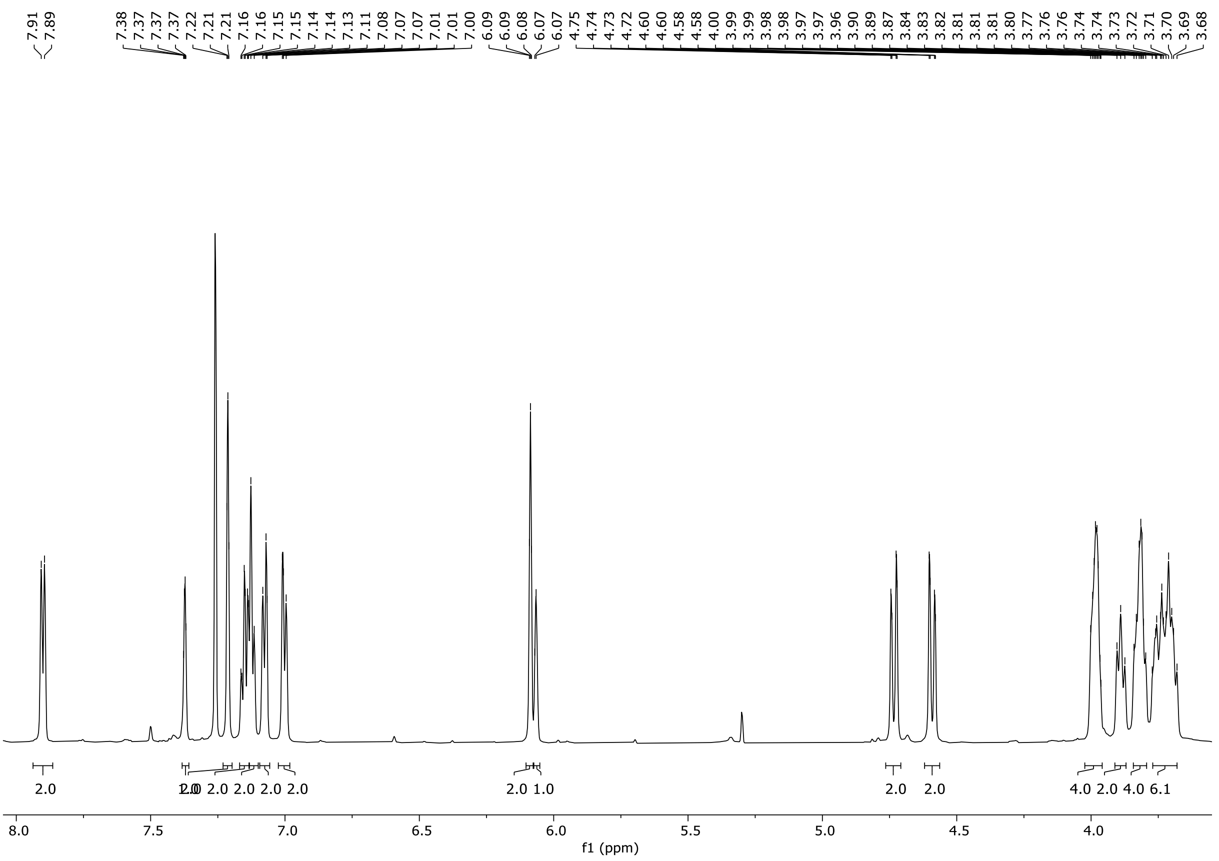

$^{13}C\{^1H\}$-NMR (151 MHz, Chloroform-*d*, 298 K)

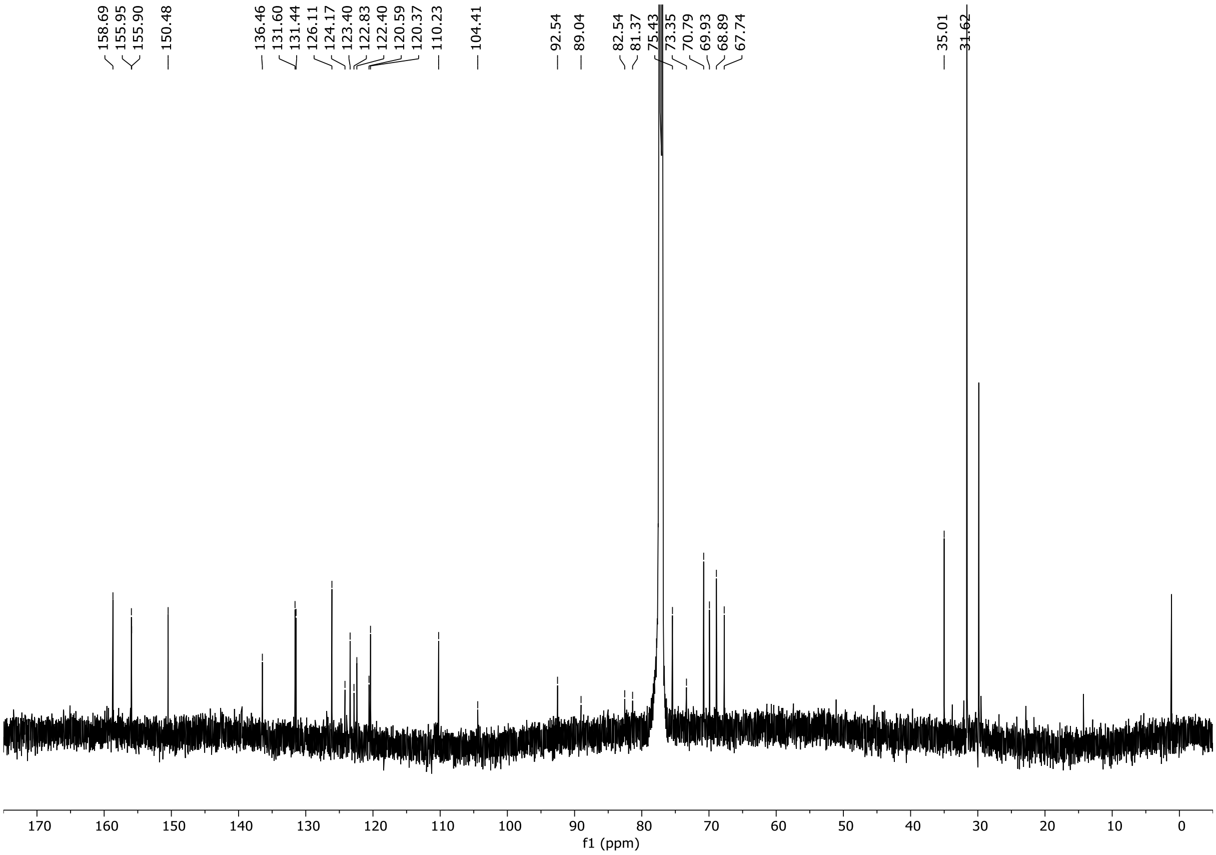

HSQC (600 Hz, Chloroform-*d*, 298 K)

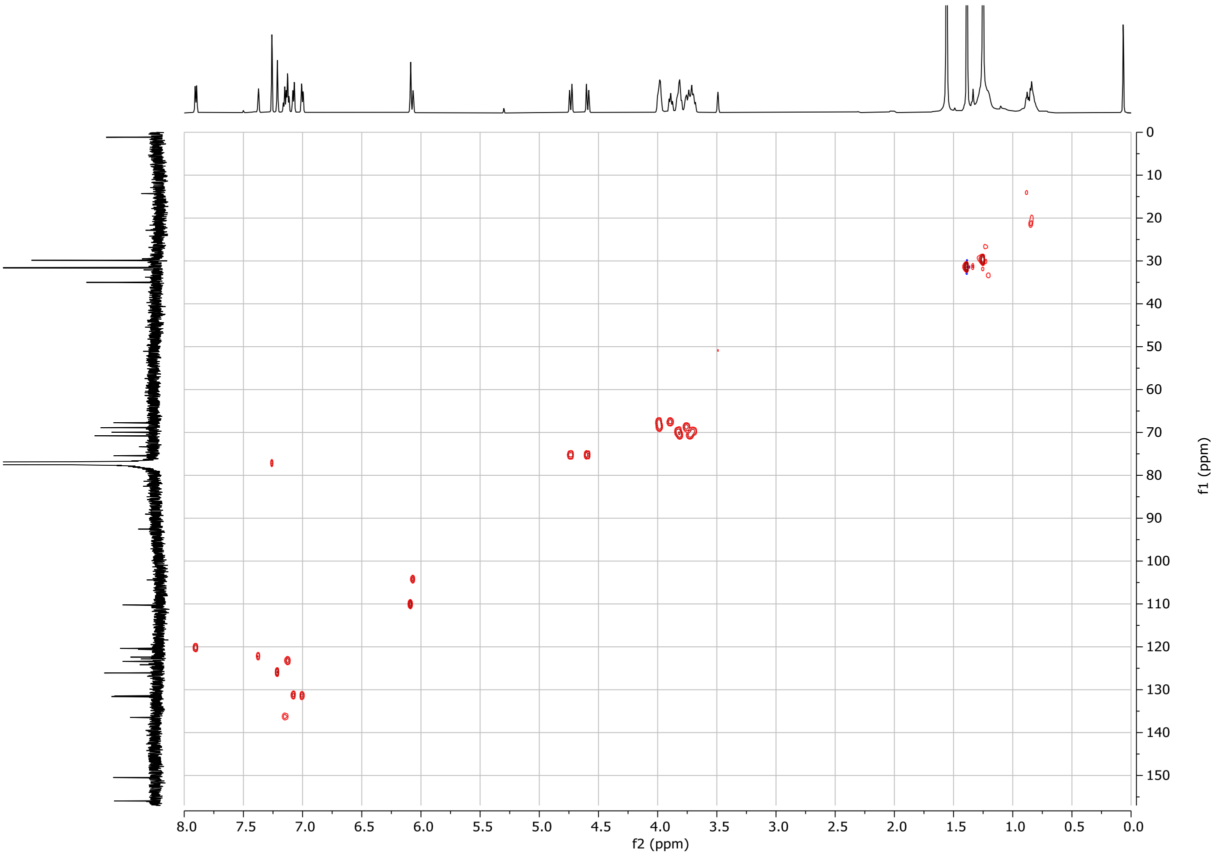

HMBC (600 Hz, Chloroform-*d*, 298 K)

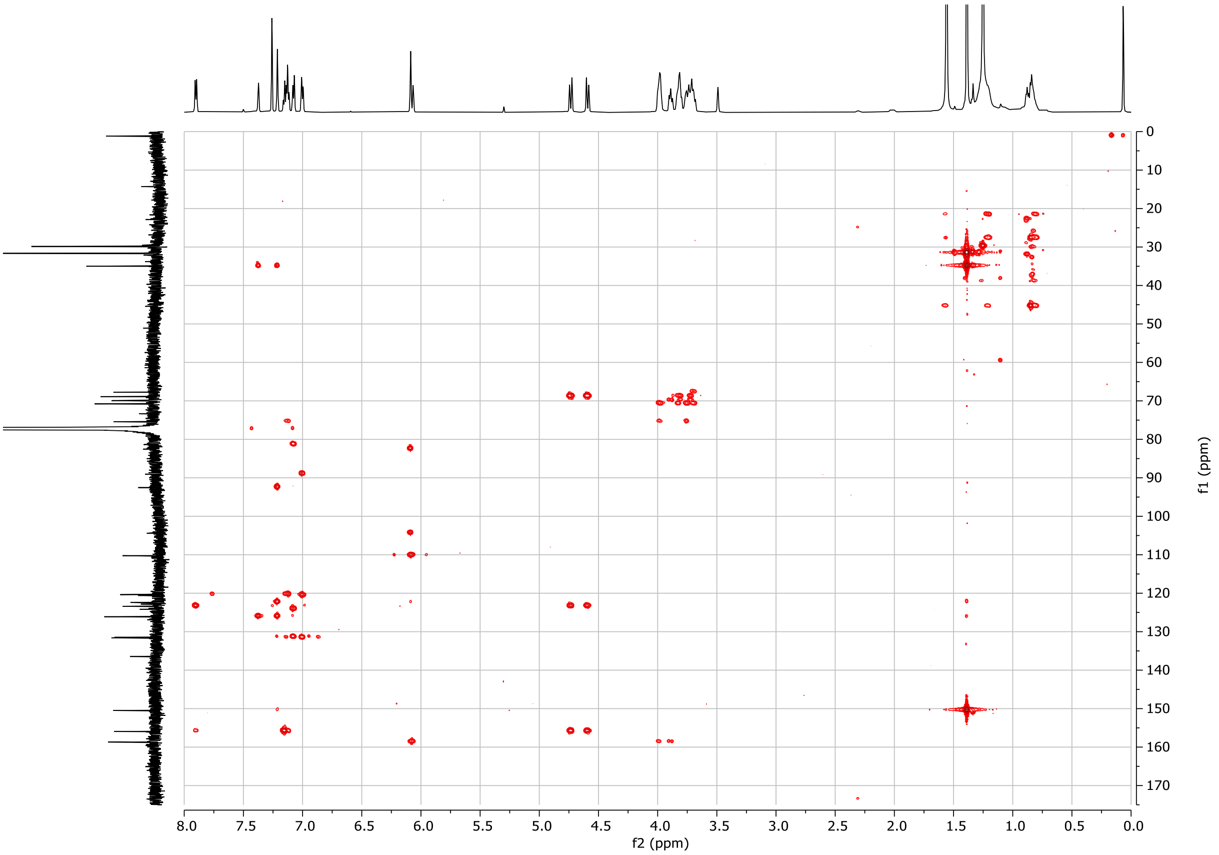

COSY (600 Hz, Chloroform-*d*, 298 K)

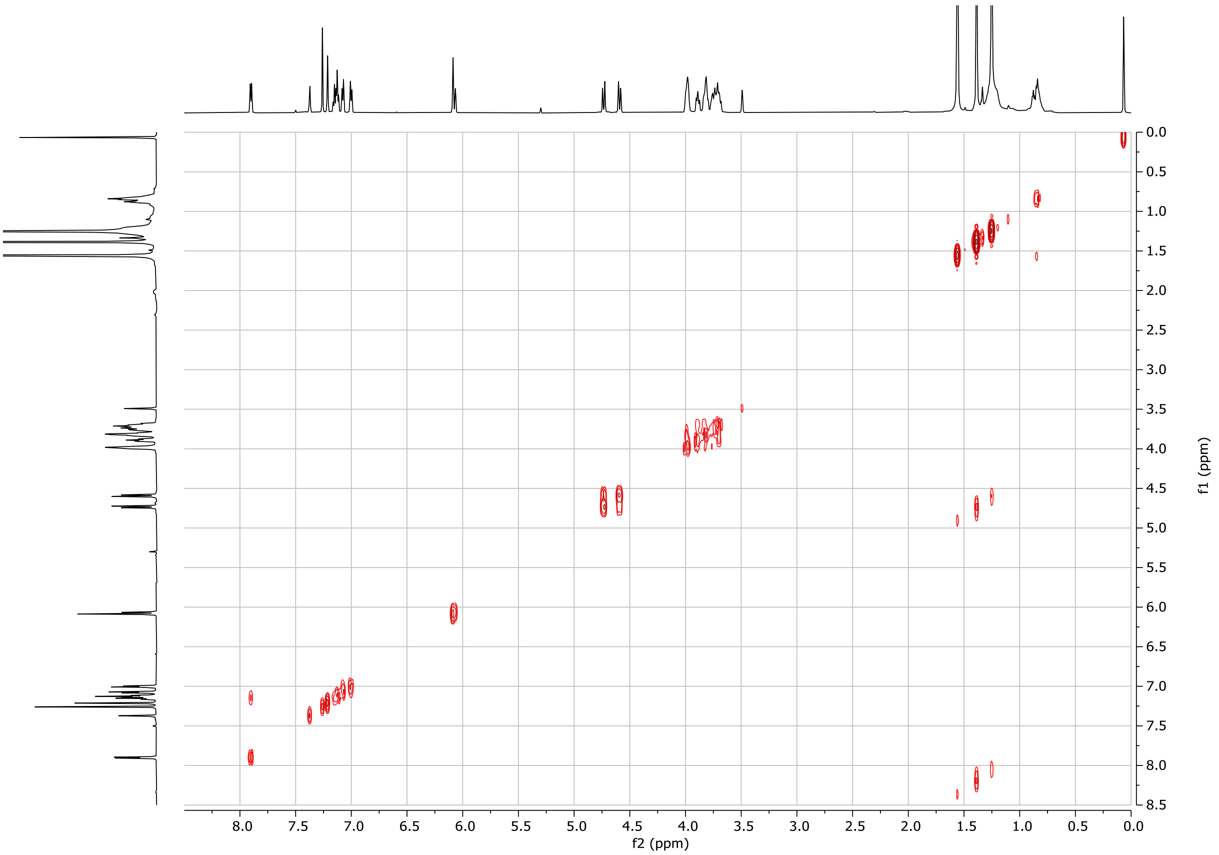

ROESY (600 Hz, Chloroform-*d*, 298 K)

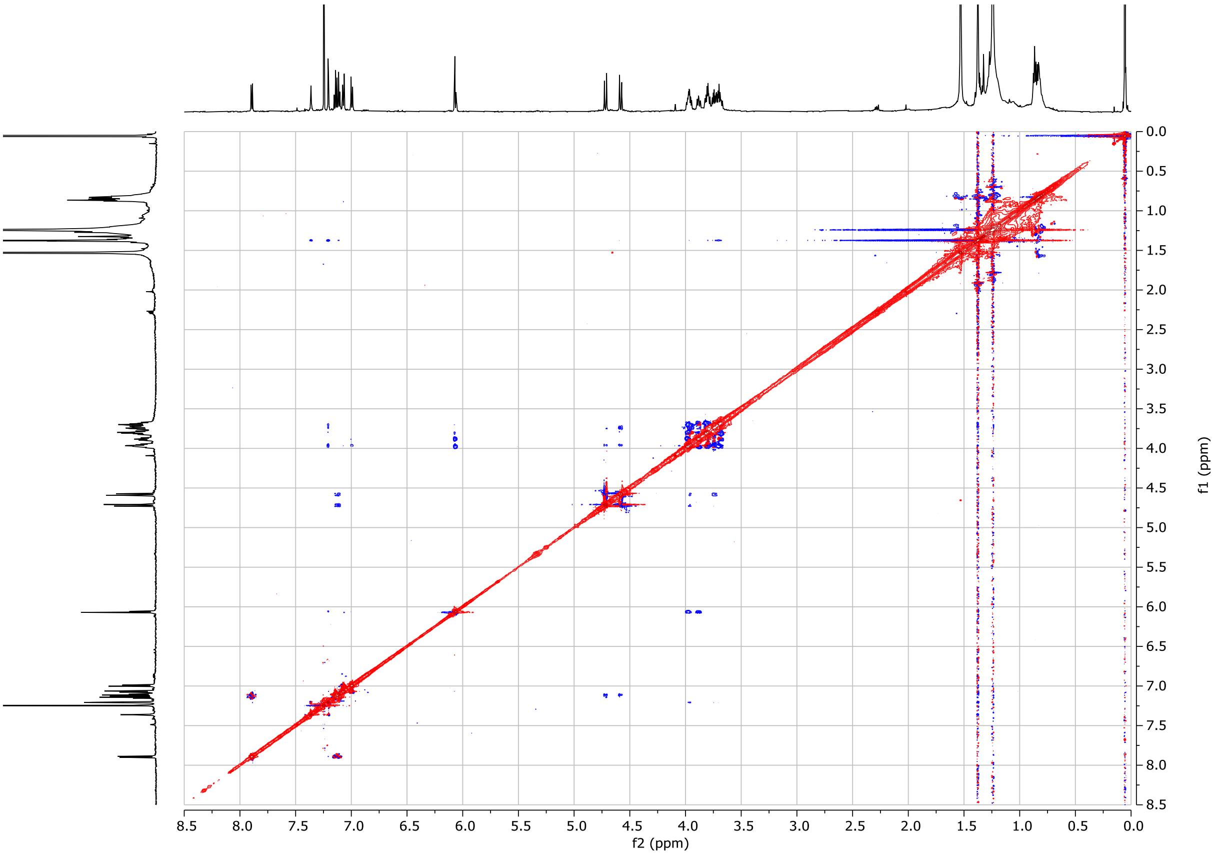

HRMS (APCI, +)

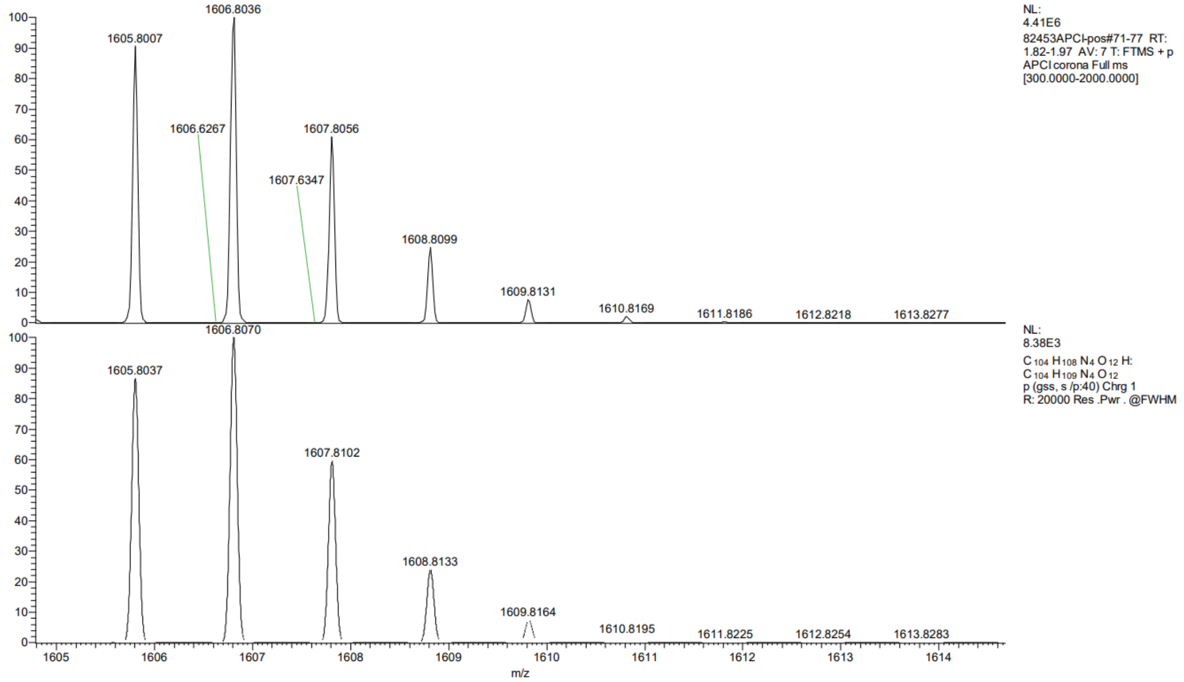

Tandem HRMS (APCI, +):

Using 35 eV collision energy:

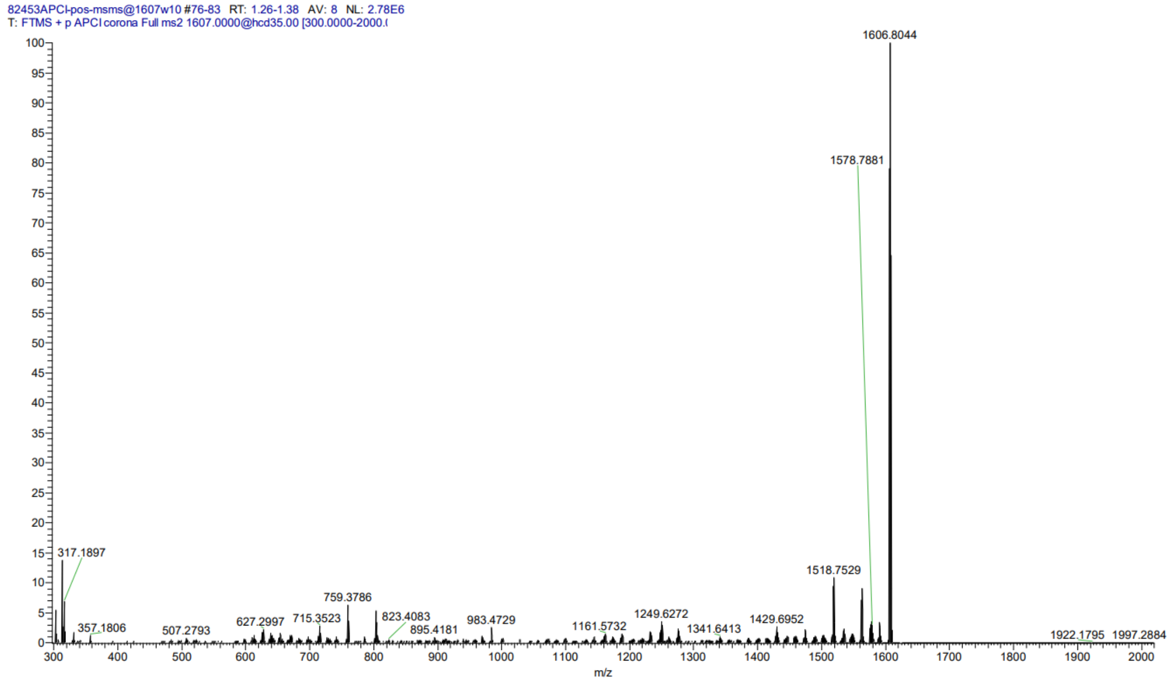

Using 40 eV collision energy:

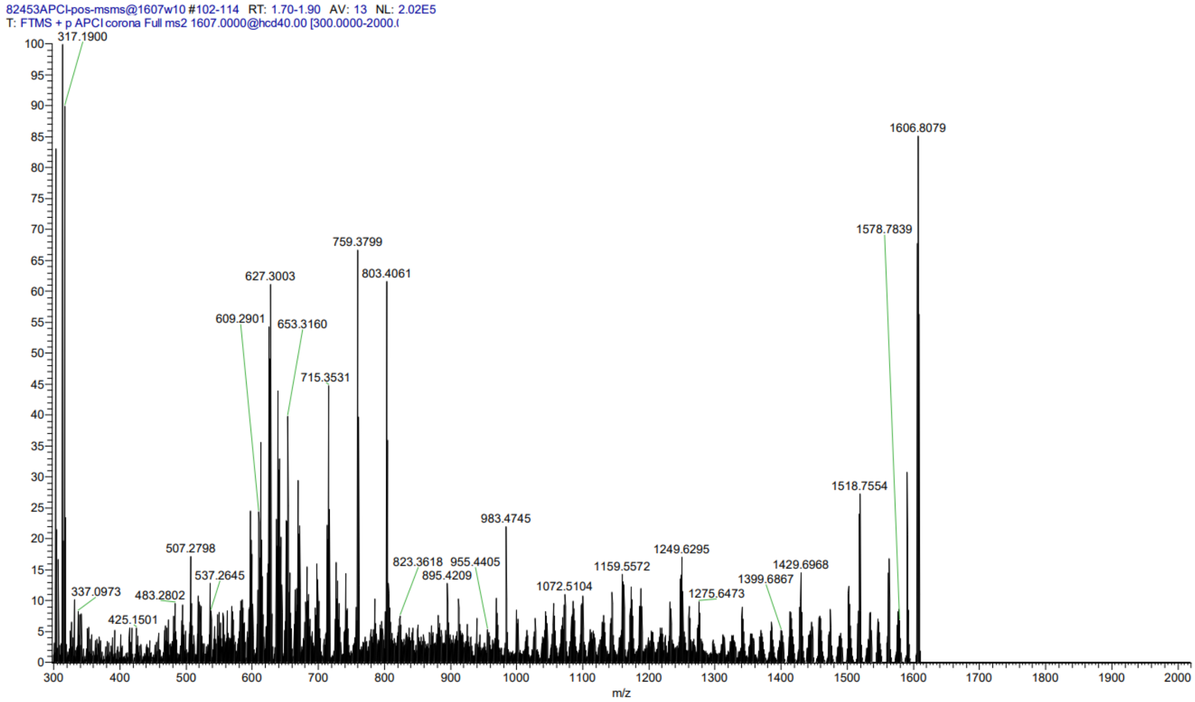

Using 43 eV collision energy:

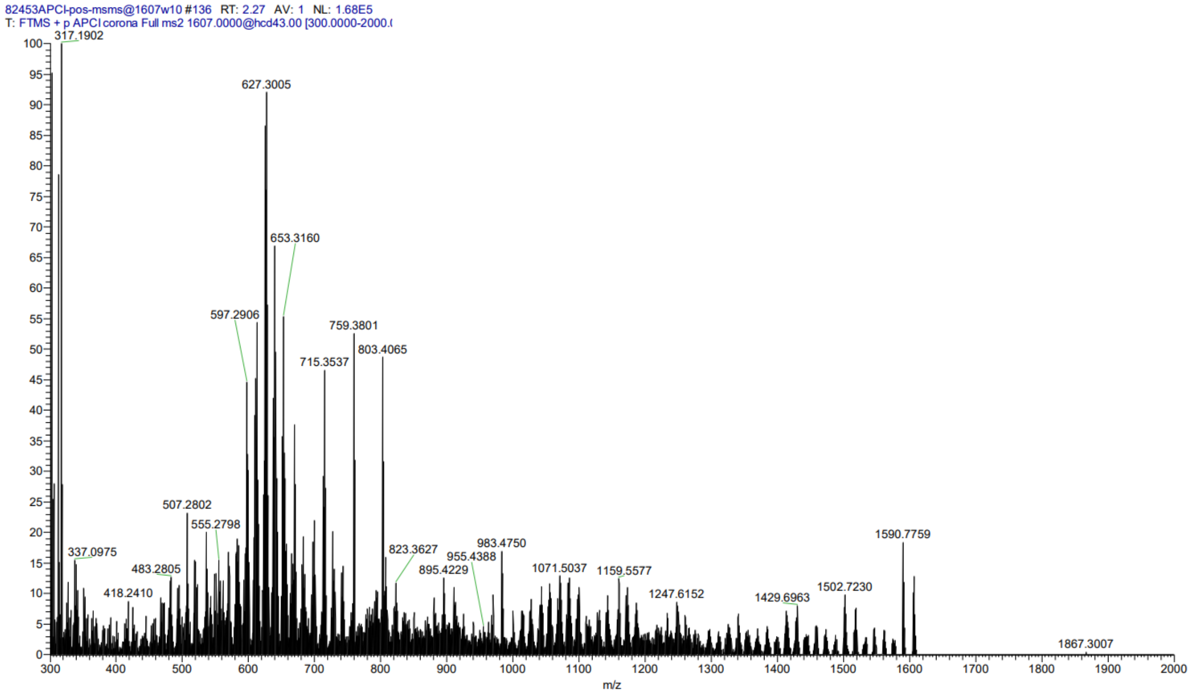

Using 45 eV collision energy:

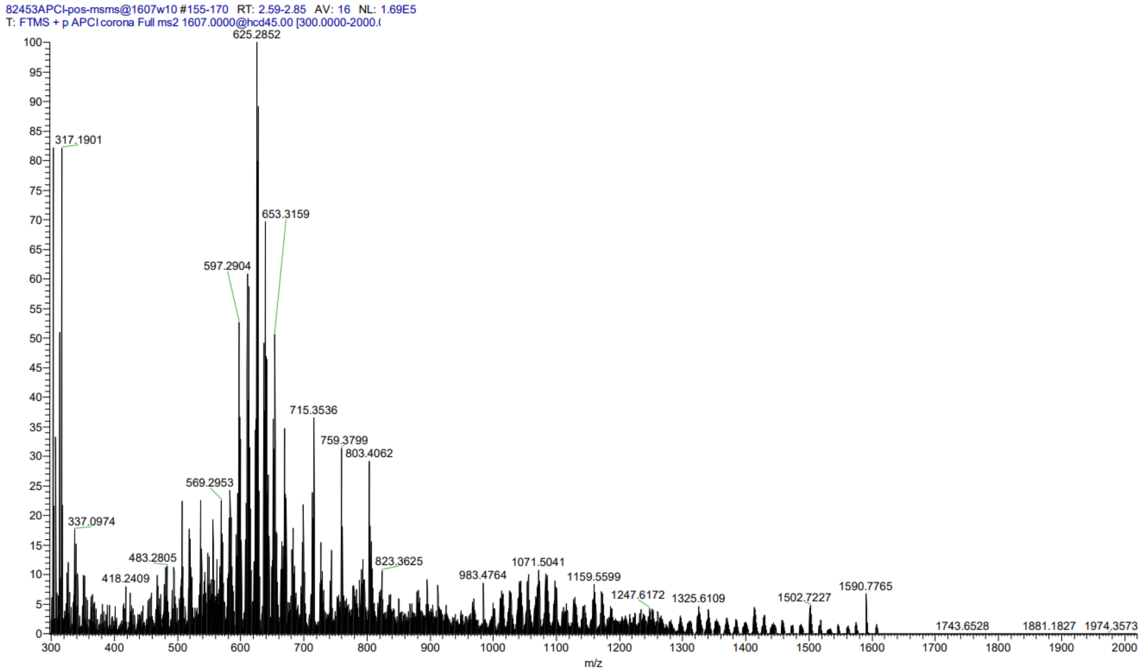

UV/VIS (CH_2Cl_2, 20 °C)

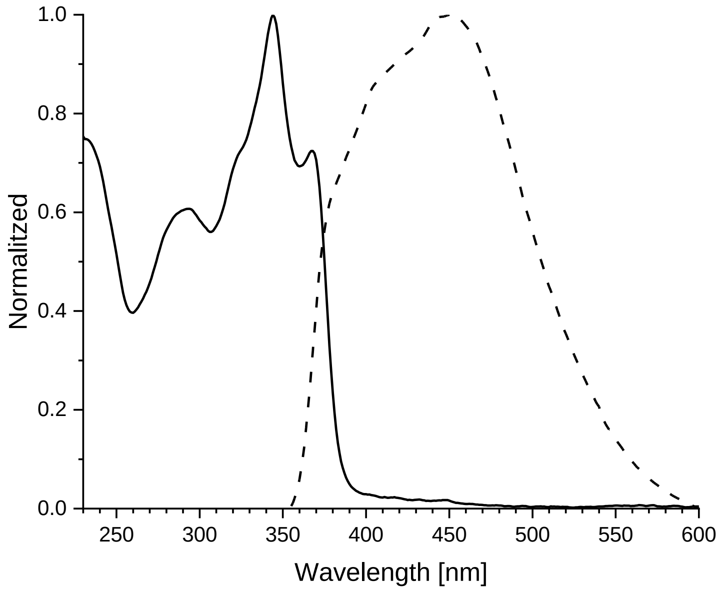

Variable Temperature UV-Vis Study of the Daisy Chain **1**

Variable temperature experiments were performed in 1,2-dichloroethane between 10 °C and 80 °C by recording a spectrum every 10 °C as displayed in the Figure 2 below. All spectra show a similar absorption and poor vibronic resolution, indicating that at all temperatures (also at 10 °C) different geometries can be populated and no thermodynamically most stable state (eg. the contracted or the extended form of the molecular daisy chain) can be identified or isolated. Overall larger changes in relative extinction coefficient can be observed for the spectral region attributed to the rigid backbone. This indicates, that the thermal energy mainly influences the relative populations of different geometries and positions of the OPE axle.

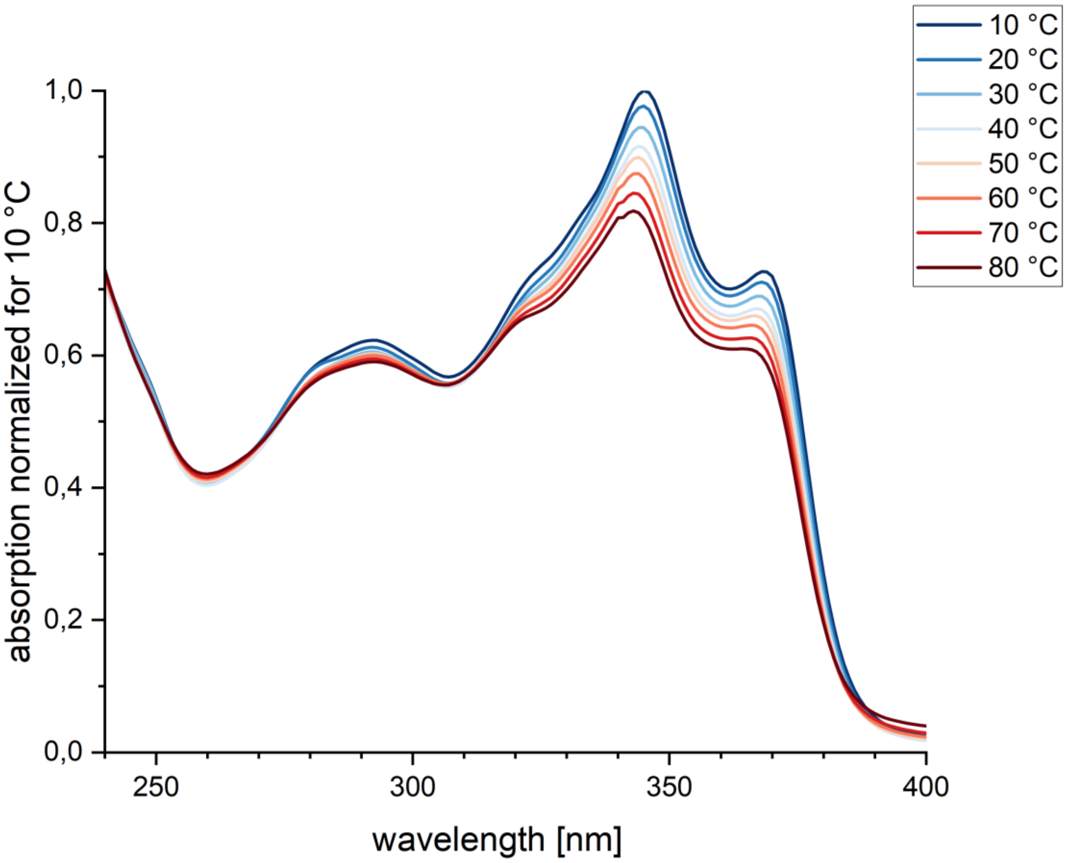

Figure 2 Absorption of the [c2] daisy chain **1** in 1,2-dichloroethane at different temperatures. The spectra are normalized to the first measurement at 10 °C.

Aggregation Studies with the Daisy Chain **1**

To investigate the influence of protic polar solvents and aggregation or precipitation towards **1**, absorption spectra with different dichloromethane to methanol ratios (as reported in the main text for **4**) were recorded and are reported in Figure 3 bellow. The experiment showed only minor changes in the absorption when increasing the volume ratio of methanol. The blue shift for the spectral region attributed to the OPE axle of **1** was observed in similarity to the monomer **4**. Overall, the experiment revealed no influence of the solvent polarity towards the distribution of daisy chain conformations.

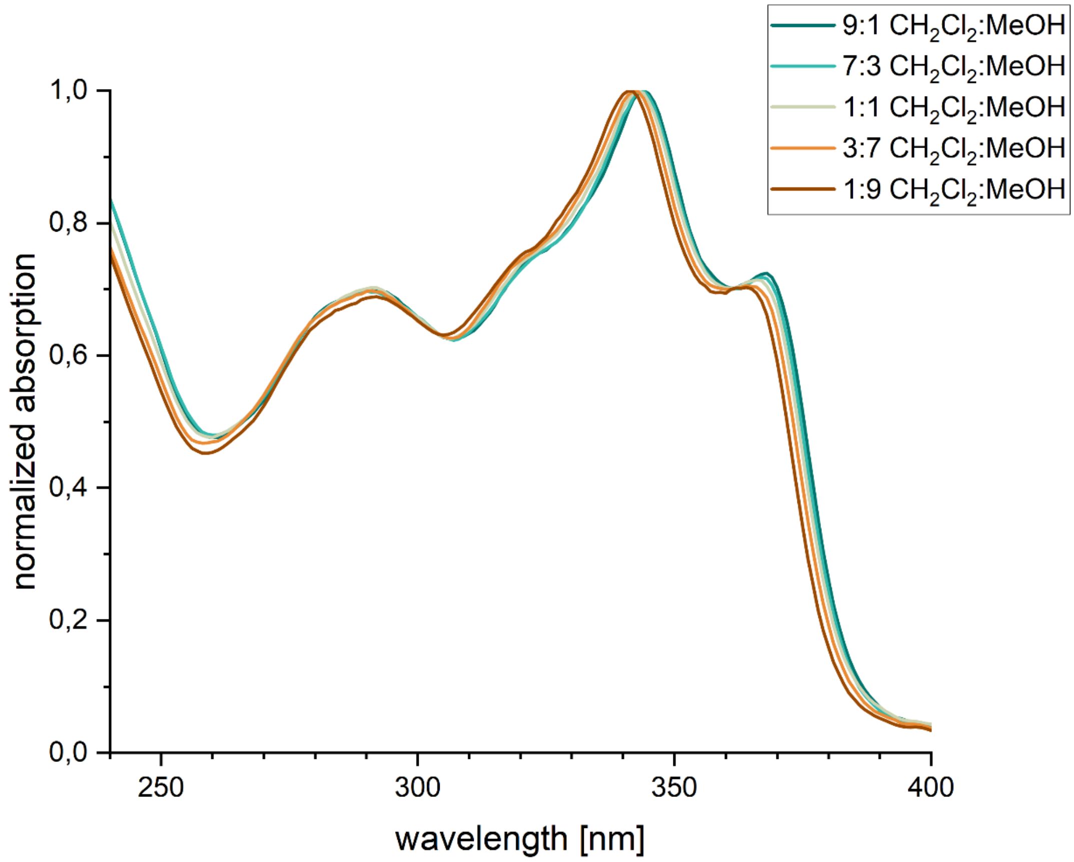

Figure 3 Absorption of the [c2] daisy chain **1** in different dichloromethane to methanol ratios measured at 20 °C. The spectra are normalized.

References

[28] W. Li, J.-H. Xie, M.-L. Yuan, Q.-L. Zhou, *Green Chem.* **2014**, *16*, 4081–4085.

[27] J. E. M. Lewis, R. J. Bordoli, M. Denis, C. J. Fletcher, M. Galli, E. A. Neal, E. M. Rochette, S. M. Goldup, *Chem. Sci.* **2016**, *7*, 3154–3161.

[8] J. Rotzler, S. Drayss, O. Hampe, D. Häussinger, M. Mayor, *Chem. Eur. J.* **2013**, *19*, 2089–2101.
